# Tailoring the natural rare sugars D-tagatose and L-sorbose to produce novel functional carbohydrates

**DOI:** 10.1038/s41538-024-00320-8

**Published:** 2024-10-04

**Authors:** Oswaldo Hernandez-Hernandez, Carlos Sabater, Inés Calvete-Torre, Elisa G. Doyagüez, Ana M. Muñoz-Labrador, Cristina Julio-Gonzalez, Blanca de las Rivas, Rosario Muñoz, Lorena Ruiz, Abelardo Margolles, José M. Mancheño, F. Javier Moreno

**Affiliations:** 1 Institute of Food Science Research, CIAL (CSIC-UAM), Nicolas Cabrera 9, 28049 Madrid, Spain; 2 Dairy Research Institute of Asturias (IPLA-CSIC), Paseo Río Linares s/n, 3300 Villaviciosa, Asturias Spain; 3 Health Research Institute of Asturias (ISPA), Avenida Hospital Universitario s/n, 33011 Oviedo, Asturias Spain; 4 Centro de Química Orgánica “Lora Tamayo” (CSIC), Juan de la Cierva 3, 28006 Madrid, Spain; 5 Institute of Food Science, Technology and Nutrition, ICTAN (CSIC), Juan de la Cierva 3, 28006 Madrid, Spain; 6 Institute of Physical Chemistry ‘Blas Cabrera’ (IQF-CSIC), Serrano 119, 28006 Madrid, Spain

**Keywords:** Dietary carbohydrates, Hydrolases

## Abstract

This multidisciplinary study details the biosynthesis of novel non-digestible oligosaccharides derived from rare sugars, achieved through transfructosylation of D-tagatose and L-sorbose by levansucrase from *Bacillus subtilis* CECT 39 (SacB). The characterization of these carbohydrates using NMR and molecular docking was instrumental in elucidating the catalytic mechanism and substrate preference of SacB. Tagatose-based oligosaccharides were higher in abundance than L-sorbose-based oligosaccharides, with the most representative structures being: β-D-Fru-(2→6)-β-D-Fru-(2→1)-D-Tag and β-D-Fru-(2→1)-D-Tag. In vitro studies demonstrated the resistance of tagatose-based oligosaccharides to intestinal digestion and their prebiotic properties, providing insights into their structure-function relationship. β-D-Fru-(2→1)-D-Tag was the most resistant structure to small-intestinal digestion after three hours (99.8% remained unaltered). This disaccharide and the commercial FOS clustered in similar branches, indicating comparable modulatory properties on human fecal microbiota, and exerted a higher bifidogenic effect than unmodified tagatose. The bioconversion of selected rare sugars into β-fructosylated species with a higher degree of polymerization emerges as an efficient strategy to enhance the bioavailability of these carbohydrates and promote their interaction with the gut microbiota. These findings open up new opportunities for tailoring natural rare sugars, like D-tagatose and L-sorbose, to produce novel biosynthesized carbohydrates with functional and structural properties desirable for use as emerging prebiotics and low-calorie sweeteners.

## Introduction

According to the International Society of Rare Sugars (ISRS, https://www.isrs.kagawa-u.ac.jp/), rare sugars are monosaccharides and their derivatives that are scarce in nature^1^. Main groups of rare sugars include ketohexoses such as tagatose, sorbose, and psicose (in both D- and L-configurations), polyols like xylitol, and deoxygenated monosaccharides such as L-ribose. Despite their low natural abundance, rare sugars have significant potential for practical applications in the food, pharmaceutical, and nutrition industries due to their biological functions^2–4^. The Izumoring strategy, developed by Prof. Izumori at the Rare Sugar Research and Education Center of Kagawa University in Japan, has been a leading approach for synthesizing rare sugars for twenty years. This method relies on the principle that monosaccharides can be cyclically converted through enzymatic processes, including epimerization, isomerization, and oxidation-reduction^5,6^. However, recently, several non-Izumoring enzymatic techniques have emerged, based on different principles such as aldose epimerization, enzymatic condensation, phosphorylation-dephosphorylation cascade reactions, dicarboxylic reactions, and the enzymatic synthesis of novel disaccharides using rare sugars as acceptors^7^. Notably, only a few papers have addressed the latter approach, mainly focusing on the xylosylation of D-psicose (also known as D-allulose)^6^, or the glucosylation of D-galactose, D-ribose, L-arabinose, or D-psicose catalyzed by bacterial sucrose phosphorylases^8,9^. Fujita et al. ^10^ also described the transfructosylation of L-sorbose to form a single disaccharide with the glycosidic linkage β-(2 → 2) catalyzed by a β-fructofuranosidase from *Microbacterium saccharophilum* K-1 (formerly known as *Arthrobacter* sp. K-1). These studies suggest that carbohydrates containing rare sugars could have interesting physiological activities and potentially higher functionality than the rare sugars themselves. However, as Zhang et al. ^11^ highlighted, there is a lack of literature on the specific physiological functions of oligosaccharides containing rare sugars, making this a key area of future research in rare sugar studies.

D-Tagatose shares physical properties with sucrose in terms of color, texture, and sweetness, but appears to have a much lower glycemic index and caloric value due to its low bioavailability^12^. As a result, its properties as a low-calorie sweetener make D-tagatose the most demanded and commercially important rare sugar. The U.S. Food and Drug Administration (FDA) categorized D-tagatose as a Generally Recognized as Safe (GRAS) substance two decades ago^11^. Subsequently, it was authorized as a novel food in Australia and New Zealand^13^, and in the European Union, based on an opinion from the UK Advisory Committee for Novel Foods and Processes (ACNFP)^14^. The use of D-tagatose in food products and beverages is also accepted in other countries, including South Africa and South Korea^15,16^. Moreover, tagatose has shown potential beneficial effects and therapeutic properties in humans and has been proposed for treating conditions like “type 2” diabetes, hyperglycemia, anemia, and hemophilia, as well as for promoting gut health through its prebiotic properties^11,17^. However, the role of tagatose as a prebiotic ingredient could be undermined by studies showing that D-tagatose is absorbed at least to some extent in the small intestine in rats^18^ and to a greater extent in humans^19^. A study in humans indicated a mean absorption rate of 80% (range 69–88%) in the small intestine and a urinary excretion of either 1% or 5%, so only the remaining 20% of D-tagatose could be fermented in the colon. These values were accepted and used by the European Food Safety Authority (EFSA) to estimate the energy gain from absorbed and metabolized D-tagatose^20^. Based on these findings, the use of enzymes with transglycosidase activity to tailor tagatose through selective elongation could yield novel carbohydrates with higher functionality than tagatose itself. Specifically, the enzymatic production of a disaccharide resulting from tagatose fructosylation could not only mimic the physical properties of sucrose but also offer significant refinement. This is because such a disaccharide would likely resist gastrointestinal digestion and pass through the small intestine, becoming available for consumption by the colonic microbiota to a greater extent than tagatose, and with different selectivity due to key structural modifications.

Therefore, this article reports the biosynthesis of novel non-digestible tagatose-based oligosaccharides through a transfructosylation reaction catalyzed by a levansucrase from *Bacillus subtilis* CECT 39 (SacB). Additionally, other rare sugars such as L-sorbose and D-psicose were tested as potential acceptors for producing novel carbohydrates, while molecular docking further verified the feasibility of these substrates and enzyme binding. A comprehensive structural characterization of the biosynthesized D-tagatose- and L-sorbose-oligosaccharides was accomplished by NMR. In vitro studies demonstrating the resistance to intestinal digestion and prebiotic properties of the main tagatose-based disaccharide provided insights into its structure-function relationship.

## Results and Discussion

### Acceptor specificity of SacB

Levansucrases (also known as sucrose:2,6-β-D-fructan 6-β-D-fructosyltransferases or beta-2,6-fructosyltransferases, EC 2.4.1.10) catalyze both sucrose hydrolysis and the β-(2,6)-linked levan polymerization. Likewise, in the presence of appropriate substrate acceptors other than water levansucrases are among the most used and effective microbial fructansucrases in oligosaccharide synthesis from sucrose^21,22^. The ‘carbohydrate-active enzymes’ database (CAZY, http://www.cazy.org/)^23^ groups bacterial levansucrases into glycoside hydrolase (GH) family 68. The levansucrase SacB from *Bacillus subtilis* (https://www.uniprot.org/uniprot/P05655; https://www.ncbi.nlm.nih.gov/protein/P05655.1) is likely one of the most studied fructansucrases. It consists of a 53 kDa (including the signal peptide) single-domain protein with a five-bladed propeller fold that encloses a substrate-binding central cavity (*i.e*., active site) located in a deep pocket surrounded by several semi-conserved amino acid residues^24^. The catalytic mechanism of SacB is based on the double-displacement reaction mechanism with retention of the anomeric configuration of the transferred fructosyl moieties coordinated by three catalytic residues (Asp86, Asp247, and Glu342)^24,25^. Briefly, the catalytic mechanism involves the protonation of the glycosidic oxygen by a carboxylate acting as a general acid catalyst, most probably Glu342^24^, concurrently with cleavage of the glycosidic bond in a fructosyl donor (usually a sucrose molecule). This process takes place between the −1 and +1 subsites, according to the nomenclature proposed by Davies et al. ^26^, within the active site of SacB. This first step results in the formation of a fructosyl-SacB covalent intermediate, with Asp86 acting as the nucleophile, and the release of the glucose moiety. In the second step, the covalently-bound fructosyl moiety is transferred from Asp86 to an acceptor molecule with retention of the anomeric configuration, leading to the elongation of the substrate acceptor in one fructosyl unit^27^. The acceptor specificity of SacB has been explored for a long time by using a range of carbohydrates and/or derivatives as fructosyl acceptors in reactions using sucrose as donor substrate^25,28–33^. However, to the best of our knowledge, the acceptor specificity of SacB on rare sugars such as D-tagatose, L-sorbose, or D-psicose has never been tackled.

**Fig. 1:**
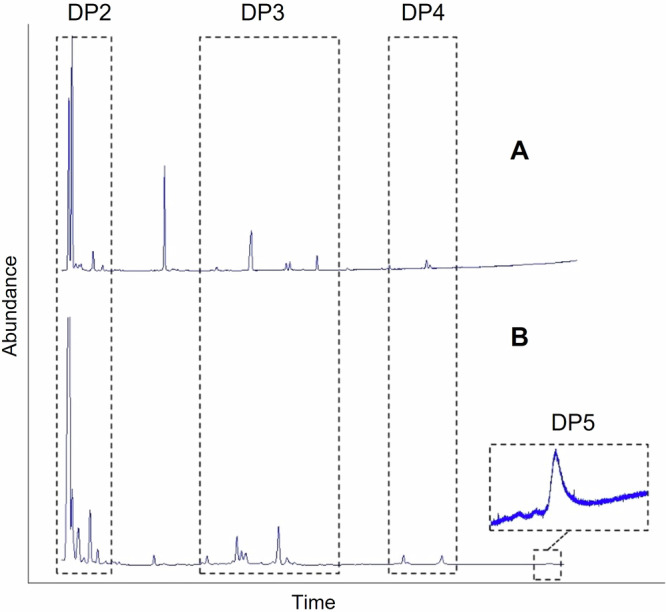
GC-FID profiles of the reaction products catalyzed by the levansucrase SacB from *Bacillus subtilis* CECT 39. Starting substrates: (**A**) D-Sucrose:D-Tagatose, (**B**) D-Sucrose:L-Sorbose both at a concentration ratio of 200: 200 g L^−1^. DP: degree of polymerization. DP2: Disaccharide fraction; DP3: Trisaccharide fraction; DP4: Tetrasaccharide fraction; DP5: Pentasaccharide fraction.

Figure 1 shows the GC-FID profiles resulting from the enzymatic reaction mixtures catalyzed by SacB using as starting substrates D-sucrose:D-tagatose and D-sucrose:L-sorbose at a concentration ratio of 200: 200 g L^−1^ (mole ratio of D-sucrose to L-sorbose or D-tagatose is 1 : 1.9). Both D-tagatose and L-sorbose seemed to be good acceptors since new products having elution times compatible with the presence of carbohydrates with a degree of polymerization from 2 to 5 could be detected (Fig. 1A, B), whereas the presence of D-psicose did not lead to the formation of detectable new products (data not shown). D-Tagatose and L-sorbose vary in two positions at C-4 and C-5 sites for the placement of the -OH group, whereas D-tagatose and D-psicose have these variations at C-3 and C-4 sites, and L-sorbose and D-psicose at C-3 and C-5 sites (Fig. S1). According to the catalytic mechanism of SacB explained above, we would expect the formation of disaccharides resulting from the transfer of a fructosyl residue from sucrose to any of the hydroxyl groups available of D-tagatose and L-sorbose. Likewise, the new products having a degree of polymerization above 2 could be derived from further transfructosylation reactions, resulting in chain elongation and oligomer formation.

In the next subsection, the comprehensive structural characterization accomplished by NMR of the biosynthesized D-tagatose- and L-sorbose-carbohydrates is described. Furthermore, in silico molecular docking experiments to further investigate the precise mechanism of ordered-substrate binding to SacB were carried out to verify the feasibility of these structural complexes of SacB with the tested rare sugars and to gain knowledge on its substrate specificity.

### Structural elucidation of D-tagatose and L-sorbose-fructosylated carbohydrates

NMR characterization was accomplished by the combined use of 1D and 2D [^1^H, ^1^H] and [^1^H, ^13^C] NMR experiments (gCOSY, TOCSY, multiplicity-edited gHSQC, gHMBC and hybrid experiment gHSQC-TOCSY). ^1^H and ^13^C NMR chemical shifts observed are summarized in Tables S1–S6. A full set of spectra is also available in the Supporting Information (Figures S2–S29).

*D-tagatose Derivatives:* D-tagatose derivatives was divided among three purified fractions, labeled T1, T2, and T3.

**Fig. 2:**
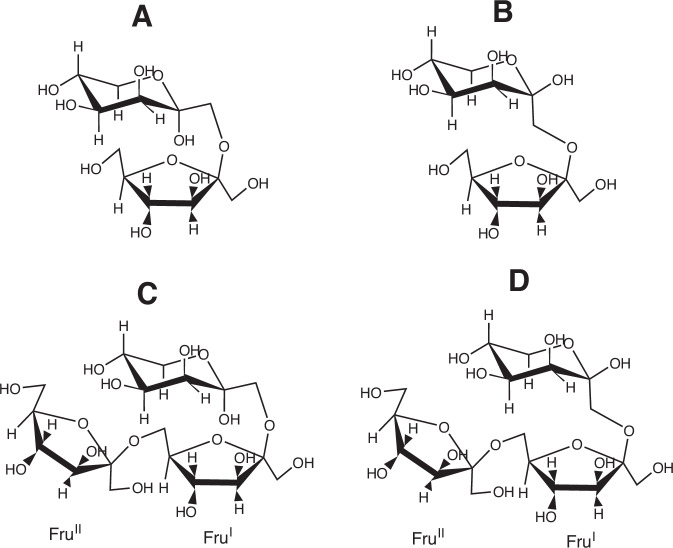
Structures accomplished by NMR of di- and trisaccharides obtained by tagatose fructosylation catalyzed by the levansucrase SacB from *Bacillus subtilis* CECT 39. **A** β-D-fructofuranosyl-(2→1)-α-D-tagatopyranose. **B** β-D-fructofuranosyl-(2→1)-β-D-tagatopyranose. **C** β-D-fructofuranosyl-(2 → 6)-β-D-fructofuranosyl-(2 → 1)-α-D-tagatopyranose. **D** β-D-fructofuranosyl-(2 → 6)-β-D-fructofuranosyl-(2 → 1)-β-D-tagatopyranose.

Purified Fraction T1: Analysis of purified fraction T1 revealed the presence of two anomers of a disaccharide structure, identified as β-D-fructofuranosyl-(2 → 1)-D-tagatopyranose, existing in a 6:1 α:β ratio (Fig. 2A, B). Notably, the 1D ^1^H NMR spectrum for this compound displayed an absence of signals in the anomeric region, attributed to the lack of anomeric protons within the fructose and tagatose units. Furthermore, the 1D ^13^C NMR spectrum revealed signals for 24 carbons, encompassing four anomeric carbons, whose chemical shifts were instrumental in establishing the pyranose and furanose forms of tagatose and fructose, respectively^34^. A detailed analysis using multiplicity-edited gHSQC spectrum facilitated the linkage of carbon signals to their corresponding proton resonances, thereby reinforcing the structural identification. The glycosidic linkages were further analyzed and confirmed through gHMBC spectrum observations, thereby affirming the (2 → 1) linkage between the constituent sugars. The assignment of α- and β- anomers for each compound was supported by ^1^H and ^13^C NMR shifts (Table S1)^34^.

Purified Fraction T2: Fraction T2 presented a divergent disaccharide structure, devoid of tagatose, identified as β-D-fructofuranosyl-(2 → 6)-D-glucopyranose. This structure was elucidated through a similar comprehensive suite of NMR analyses, including 1D and 2D spectra, which supported the presence of β- and α-glucose alongside two β-fructose units. The assignment of glycosidic linkages was corroborated through detailed gHMBC spectral analysis, confirming the (2 → 6) linkage. These results are in accord with other data from Sato et al. ^35^.

Purified Fraction T3: In fraction T3, three trisaccharides were identified, with the major compound being a complex β-D-fructofuranosyl-(2 → 6)-β-D-fructofuranosyl-(2 → 1)-D-tagatopyranose structure (Fig. 2C, D), alongside another trisaccharide in lesser proportion and a very minor signal indicative of a potential third structure, assigned as β-D-fructofuranosyl-(2 → 6)-α-D-glucopyranosyl-(1 → 2)-β-D-fructofuranoside. The NMR data, including 1D ^13^C NMR and multiplicity-edited gHSQC spectra, provided conclusive evidence for the proposed structures, with glycosidic linkages precisely determined through gHMBC spectral analysis, and in accord with other data regarding (2 → 6) linked fructoses from Jakob et al., ^36^ and Tajima et al. ^37^.

*L-sorbose Derivatives*. Four distinct fractions were analyzed, revealing different compounds within each fraction.

Purified Fraction S1: Fraction S1 offered a compelling mixture, with sucrose identified as the major component alongside α- and β-glucopyranose. Notably, this fraction featured significant compounds containing L-sorbose: the disaccharide β-D-fructofuranosyl-(2 → 5)-α-L-sorbopyranose and the trisaccharide β-D-fructofuranosyl-(2 → 6)-β-D-fructofuranosyl-(2 → 5)-α-L-sorbopyranose, as depicted in Figure S14 (compounds 6 and 7, respectively). The disaccharide, hereinafter referred to as β-D-Fru-(2 → 5)-α-L-Sor, showcased through the 1D ^13^C NMR spectrum, exhibited signals for 12 carbons, highlighting the presence of only the α-anomer of sorbose. This included two anomeric carbons indicating the pyranose form of α-sorbose and the β-furanose form of fructose^38,39^, a structure supported by multiplicity-edited gHSQC spectrum that linked carbon signals to corresponding proton resonances. Various NMR experiments, including COSY, TOCSY, and gHSQC-TOCSY, corroborated these findings, with gHMBC spectrum analysis further confirming the (2 → 5) linkage. ^1^H and ^13^C chemical shifts of C-1 and H-4 of β-Fruf, and C-5 and H-5 of sorbose also supported the proposed structures by comparison with other data described in literature^35,36,40,41^.

Fraction S2: Advancing to fraction S2, the investigation identified the same β-D-Fru-(2 → 5)-α-L-Sor as a major component, alongside new trisaccharides. Notably, one trisaccharide mirrored the structure of the major compound from fraction S1, β-D-Fru-(2 → 6)-β-D-Fru-(2 → 5)-α-L-Sor, outlined in Fig. S18 (compound 7). Additional compounds in this fraction included β-D-fructofuranosyl-(2 → 6)-α-D-glucopyranosyl-(1 → 2)-β-D-fructofuranoside (the same as found in fraction T3, depicted in Fig. S10, compound 4) and β-D-fructofuranosyl-(2 → 3)-α-D-glucopyranosyl-(1 → 2)-β-D-fructofuranoside (Figure S18, compound 8).

Fractions S3 and S4: In fraction S3, the narrative extends with the identification of two tetrasaccharides. One, denoted as compound 9 (Fig. S22), serves as an extension of the trisaccharide structure from fraction S1 by including an additional β-fructofuranose unit, illustrating the continued (2 → 6) linkage. The other, compound 10 (Fig. S22), follows a similar extension pattern but replaces L-sorbose with glucose. Fraction S4 unveils a pentasaccharide. This structure, depicted in Fig. S26 (compound 11), represents a further elongation of tetrasaccharide 9 with an additional β-fructofuranose unit, maintaining the consistent (2 → 6) linkage pattern.

### Molecular docking studies

Molecular docking were carried out using the structure of SacB (PDB entry: 1PT2) and the structures of the rare sugars D-tagatose, L-sorbose, and D-psicose. These sugar structures were retrieved from the PubChem database and verified using eLBOW from the Phenix package^42^, which allows for the geometry optimization of ligands. As indicated above, these three sugars differ in the absolute configuration of two carbon atoms in pairwise comparisons (Fig. S1). To proceed with the molecular docking with AutoDock Vina and since our interest resides in the simulation of the fructosyl transfer step to the rare sugars D-tagatopyranose, L-sorbopyranose and D-psicopyranose as acceptors, the receptor of the molecular docking with AutoDock Vina was a covalent fructosyl-enzyme intermediate where the Asp86 side chain of SacB is bound to the C2 atom of the fructosyl unit. The configuration of this latter C2 atom is the opposite of the one present in the sucrose molecule in agreement with the retention of the configuration catalytic mechanism of SacB^24^, in which the Asp86 side chain acts as a nucleophile^25,43^. Since no further changes were made regarding charges of the fructosyl atoms of the putative covalent fructosyl-enzyme intermediate, these in silico results should be mainly interpreted in qualitative terms. Nonetheless, we believe that the obtained poses of the rare sugars within the active site of SacB thus prepared provide a solid guide for interpreting in molecular terms the synthesis of the disaccharides β-D-fructofuranosyl-(2→1)-D-tagatopyranose and β-D-fructofuranosyl-(2-5)-α-L-sorbopyranose and the absence of fructosyl transfer to D-psicopyranose by SacB.

**Fig. 3:**
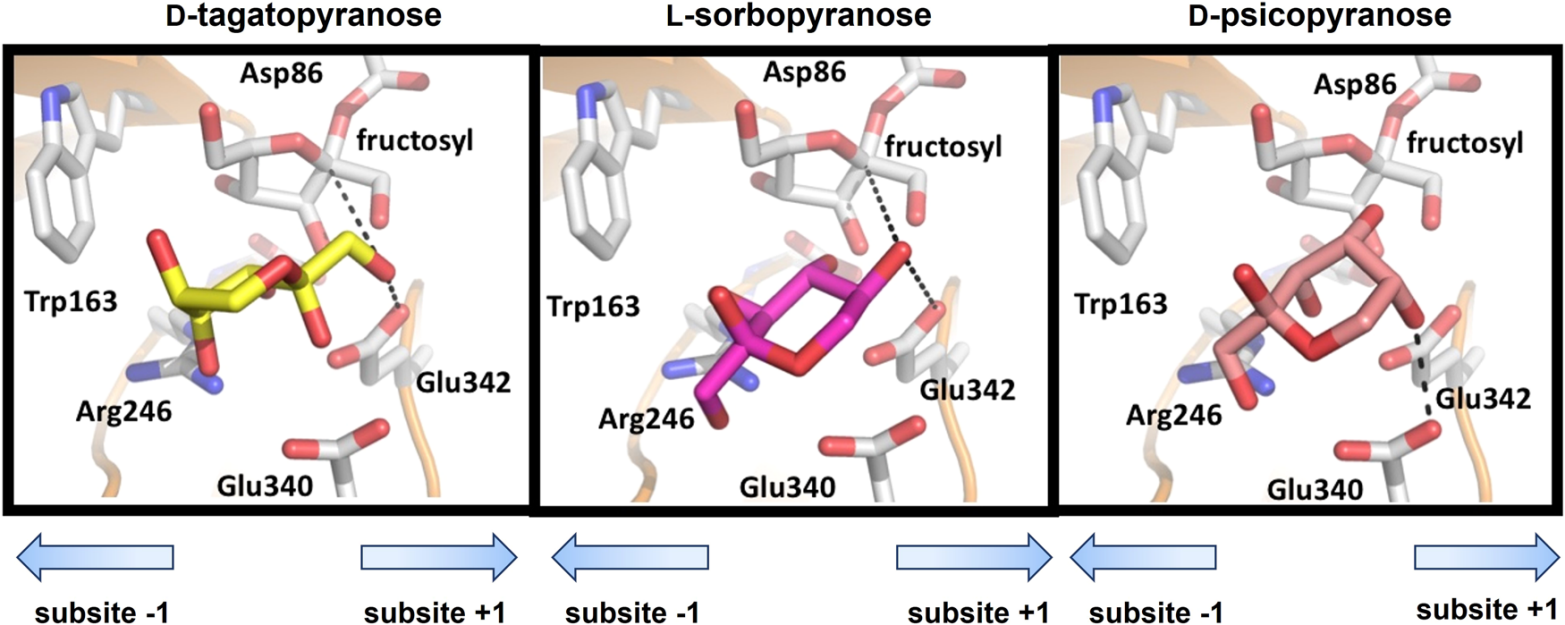
Molecular docking of the binding modes of the rare sugars tested in this study (i.e., D-tagatopyranose, L-sorbopyranose, and D-psicopyranose) to the putative covalent fructosyl intermediate of levansucrase from *Bacillus subtilis* CECT 39 (SacB). . Both the sugars and representative amino acid side chains from the SacB active site are shown as sticks model; potential H bonds are indicated by black dashed lines and putative interactions of -OH1 from D-tagatopyranose or -OH5 from L-sorbopyranose are shown as cyan dashed lines. The distinct stereochemistry of C5 of D-psicopyranose does not permit the fructosylation reaction to proceed (see the text for further details).

Figure 3 illustrates the binding features of the three rare sugars D-tagatopyranose, L-sorbopyranose, and D-psicopyranose with the receptor SacB. First, three main considerations can be made before describing the detailed sugar-protein interactions. Firstly, there is only one pose for D-tagatopyranose and for L-sorbopyranose compatible with the formation of the disaccharides β-D-fructofuranosyl-(2-1)-D-tagatopyranose and β-D-fructofuranosyl(2-5)-α-L-sorbopyranose reported in this work; secondly, these two poses agree with the catalytic mechanism proposed for the fructosyl transfer reaction by SacB^25,43^; thirdly, there is one pose for D-psicopyranose which is almost superimposable with L-sorbopyranose. Remarkably, the stereochemistry of the C5 atom of D-psicopyranose, which differs from that of L-sorbopyranose, is not compatible with the progress of the catalysis of the fructosyl transfer.

Regarding the binding mode of D-tagatopyranose (Fig. 3), possible H bonds are formed between the hydroxyl group -OH2 and the OE1 and OE2 atoms of Glu340, between the -OH3 group and the NH2 atom of Arg246 and OE2 atom of Glu342, and between the -OH4 group and the NH2 atom of Arg246. The -OH1 hydroxyl group is at H bond distance to the NE atom of Arg360 and importantly to the OE1 and OE2 atoms of the general acid-base catalytic residue Glu342. Most probably, these latter interactions properly orientate this hydroxyl group towards the C2 atom of the fructosyl unit covalently bound to Asp86 for the productive nucleophilic attack, which would explain the formation of β-D-fructofuranosyl(2-1)-β-D-tagatopyranose.

Conversely, binding of L-sorbopyranose (Fig. 3) would involve the interaction between the -OH1 with the NH2 atom of Arg246, with the ND2 and OD1 atoms from Asn242, and with the OE2 atom from Glu340, between the -OH3 hydroxyl group with the NH2 atom of Arg246 and finally, between the -OH4 hydroxyl group with the OE2 from Glu342 and with the NH2 atom of Arg246. Similar to the -OH1 group of D-tagatopyranose, the -OH5 group of L-sorbopyranose interacts with the NE atom of Arg360 and with the OE1 and OE2 atoms of Glu342, properly orienting this group towards the C2 atom of the fructosyl unit. When the poses of these two sugars are compared, it can be observed that there are pairs of equivalent -OH groups regarding their interactions with SacB: the -OH1, -OH3, and -OH4 hydroxyl groups from D-tagatopyranose would be equivalent to the -OH5, -OH4 and -OH3 from L-sorbopyranose, respectively. Most probably the hydroxyl groups -OH5 from D-tagatopyranose and -OH2 from L-sorbopyranose would interact with protein ligands through solvent molecules.

Finally, as indicated above, the binding mode of D-psicopyranose is very similar to that of L-sorbopyranose with the hydroxyl groups -OH1, -OH2, and -OH3 occupying equivalent positions as the -OH1, -OH2, and -OH4 groups from L-sorbopyranose (Fig. 3). Remarkably, the orientation (and precise position) of the -OH5 group of D-psicopyranose clearly differs from the -OH5 from L-sorbopyranose due to their different stereochemistry: axial for D-psicopyranose and equatorial for L-sorbopyranose. Now, the -OH5 group from D-psicopyranose points towards the acidic groups Glu340 and Glu342, whereas the -OH5 group from L-sorbopyranose (or -OH1 from D-tagatopyranose), in close proximity to Glu342, is directed towards the fructosyl unit. Altogether these in silico results provide a structural basis for the formation of the disaccharides β-D-fructofuranosyl-(2→1)-D-tagatopyranose and β-D-fructofuranosyl-(2-5)-α-L-sorbopyranose and also provide a plausible explanation for the lack of fructosyl transfer to D-psicopyranose.

### Enzymatic synthesis optimization of D-tagatose-fructosylated carbohydrates

Based on the widespread use and significance of D-tagatose as a low-calorie sweetener with potential prebiotic properties, an optimization was conducted focusing on the enzymatic reactions using D-tagatose as the starting substrate. This optimization took into account the SacB enzyme concentration and the donor:acceptor concentration ratios.

Initially, the concentration of SacB was optimized in reaction mixtures with initial concentrations set at 200:200 g L^−1^ of D-sucrose:D-tagatose. Among the three tested SacB concentrations (0.6, 3.1, and 6.2 U mL^−1^), the intermediate concentration of 3.1 U mL^−1^ led to the highest content of the main acceptor product, β-D-Fru-(2 → 1)-D-Tag, with a 5-fold and 13-fold increase in concentration compared to 6.2 and 0.6 U mL^−1^, respectively (data not shown).

**Fig. 4:**
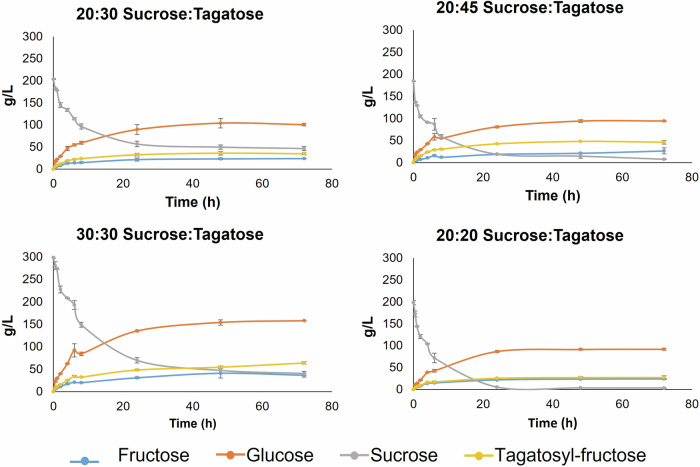
Evolution of fructose, glucose, sucrose and β-D-Fru-(2 → 1)-D-Tag (labelled as tagatosyl-fructose) following the reaction catalyzed by the levansucrase SacB from *Bacillus subtilis* CECT 39 at four different starting sucrose-to-tagatose concentration ratios (labelled as g per 100 mL of reaction). The biosynthesis of the main reaction product (i.e., β-D-Fru-(2 → 1)-D-Tag) reached a maximum at 24 h and, then, was stable for the whole reaction period (72 hours) regardless of the starting substrate concentration ratio; however, the starting substrate concentration ratio had an influence on the yield of the main reaction product.

After setting the enzyme concentration at 3.1 U/mL, the initial D-sucrose:D-tagatose concentration ratios were optimized using the ratios 200:200, 200:300, 200:450, and 300:300 g/L. Figure 4 displays the evolution of fructose, glucose, sucrose, and β-D-Fru-(2 → 1)-D-Tag in the reaction catalyzed by SacB (3.1 U mL^−1^) over 72 hours at the four different starting concentrations of D-sucrose:D-tagatose mixtures. In every instance, β-D-Fru-(2 → 1)-D-Tag synthesis commenced within the first hour of reaction, reaching maximum formation at 24 h and maintaining stability for the duration of the reaction period, signifying the high stability of the main product formed. The various D-sucrose:D-tagatose concentration ratios resulted in different maximum concentrations of β-D-Fru-(2 → 1)-D-Tag, ranging from 27 g L^−1^ (with a 200:200 g L^−1^ ratio) to 64 g L^−1^ (with a 300:300 g L^−1^ ratio). These results corroborate that an increase in the concentration of the dissolved substrates leads to greater efficiency in acceptor reactions catalyzed by glycoside hydrolases due to the reduction of water concentration^22,33^.

In terms of conversion percentages, the 300:300 and 200:450 g L^−1^ concentration ratios tested yielded the highest percentages of 21.5% and 26.0%, respectively. However, the ratios where D-tagatose was in excess relative to sucrose resulted in lower yields of β-D-Fru-(2 → 1)-D-Tag, indicating a less efficient system due to the lower conversion of D-tagatose into the product. This aligns with previous studies using this enzyme for the synthesis of the trisaccharide erlose (α-D-Glc-(1 → 4)-α-D-Glc-(1 → 2)-β-D-Fru) from sucrose/maltose reaction mixtures^33^.

To sum up, the optimal D-sucrose:D-tagatose concentration ratio was 300:300 g/L, as it produced the highest concentration of β-D-Fru-(2 → 1)-D-Tag (64.04 g L^−1^) and yield (21.3%), along with a conversion percentage (21.5%) compared to the other tested concentration ratios.

### In vitro digestibility of tagatose-based fructosylated carbohydrates by pig BBMV disaccharidases

Human data indicate that D-tagatose is absorbed in the small intestine with an average absorption rate of 80%^19,20^. Therefore, the bioconversion of D-tagatose into fructosylated species with a higher degree of polymerization could be an effective strategy to increase the bioavailability of tagatose and enhance its interaction with the gut microbiota. This would only be possible if the fructosylated tagatose remains largely unaffected by the hydrolytic activity of the mucosal disaccharidases in the mammalian small intestinal brush border membrane vesicles (BBMV), a key step in the digestion process of starchy and non-starchy carbohydrates^44^. Moreover, resistance to intestinal digestion is a critical requirement for the potential use of β-D-Fru-(2 → 1)-D-Tag and its derivatives as novel, low-calorie functional ingredients.

As expected, sucrose, which was used as a positive control in the in vitro digestion system using pig BBMV, was readily digested and was no longer detectable after 1 h (Table 1). The degrees of resistance of β-D-Fru-(2 → 1)-D-Tag and the trisaccharide β-D-Fru-(2 → 6)-β-D-Fru-(2 → 1)-D-Tag were determined following the in vitro digestion of their purified forms isolated from the enzymatic reaction mixture (included in T1 and T3 fractions). Our data suggest that these novel tagatose-based fructosylated carbohydrates are highly resistant to the hydrolytic action of the mucosal disaccharidases in pig small intestinal BBMV throughout the entire 3-hour digestion period, supporting their potential as low-calorie ingredients (Table 1). Inulin-type fructooligosaccharides (FOS), known for their β-(2 → 1) glycosidic linkages between fructose residues, have previously demonstrated similar resistance to mammalian digestive enzymes^45,46^.

**Table 1:** Determination of sucrose, β-D-fructofuranosyl-(2→1)-D-tagatopyranose and β-D-fructofuranosyl-(2→6)-β-D-fructofuranosyl-(2→1)-D-tagatopyranose during in vitro small intestinal digestion

Digestion time (h)	Sucrose	β-D-Fru-(2→1)-D-Tag	β-D-Fru-(2→6)-β-D-Fru-(2→1)-D-Tag
0	100.0 (3.5)	100.0 (17.5)	100.0 (0.5)
1	Not detected	110.6 (17.3)	88.3 (5.6)
2	Not detected	104.8 (11.8)	86.0 (4.6)
3	Not detected	99.8 (17.7)	78.0 (13.3)

Data are expressed as the mean with the SD in brackets (*n* = 3) and represent the percentage of carbohydrate determined against its initial concentration.

Therefore, the low digestibility or indigestibility of the biosynthesized tagatose-based carbohydrates calls for further studies on their fermentability and potential to selectively modulate the gut microbiota.

### In vitro fermentation properties of β-D-Fru-(2→1)-D-Tag using human fecal samples

The modulatory effect of the main product derived from the transfructosylation of tagatose, *i.e*. β-D-Fru-(2→1)-D-Tag on the human fecal microbiota, was compared to those of commercial (unmodified) tagatose and FOS. For this purpose, batch fecal in vitro fermentations using fecal pool inoculums from healthy donors were carried out. Aliquots were collected at different fermentation times (0, 8, 24 h) and microbiome composition profiles were determined by 16S rRNA sequencing. Alpha diversity coefficients were first calculated to assess the variability of genera within fecal homogenates (fecal pool inoculums corresponding to the initial fermentation time, 0 h, n = 2): Chao1 (78.50 ± 7.78), Shannon (3.53 ± 0.18), Simpson (0.94 ± 0.02) and Inverse Simpson (18.86 ± 7.63). These coefficients showed a low amount of variability between fecal inocula and reflected similar patterns in the microbiota composition of homogenates.

Then, beta diversity analysis of samples subjected to in vitro fecal fermentation was performed to assess differences in microbial diversity among substrates (FOS and unmodified tagatose controls, and β-D-Fru-(2→1)-D-Tag). For this purpose, Bray-Curtis dissimilarity coefficients were calculated (Figure S30) showing no significant differences (*p* > 0.05) among substrates. Beta diversity distances were also used to generate a dendrogram where samples corresponding to the same fecal pool inoculum clustered together (Figure S31). In addition, samples were clustered according to the fermentation time (0, 8 or 24 h). These results highlight the effect of in vitro fermentation time and variability in the microbiota composition attributed to different fecal inocula. β-D-Fru-(2→1)-D-Tag and commercial FOS were clustered in the same branches revealing similar modulatory properties of microbial communities of these two substrates compared to unmodified tagatose control.

A Principal Coordinates Analysis (PCoA) (Figure S32) was computed to better characterize sample distribution. Samples corresponding to each fecal pool inoculum were completely discriminated. In general, samples were also classified based on the fermentation time (0, 8 and 24 h). The microbiota composition barplot revealed characteristic bacterial genera profiles for each substrate (Figure S33). In this regard, fecal fermentation of commercial FOS and β-D-Fru-(2→1)-D-Tag led to high abundances of *Mitsuokella* and *Bifidobacterium* while fecal fermentation of unmodified tagatose control led to a high *Collinsella* abundance. These results emphasize differences in the microbiota modulatory properties between original and modified tagatose substrates that could be attributed to their structural features.

**Fig. 5:**
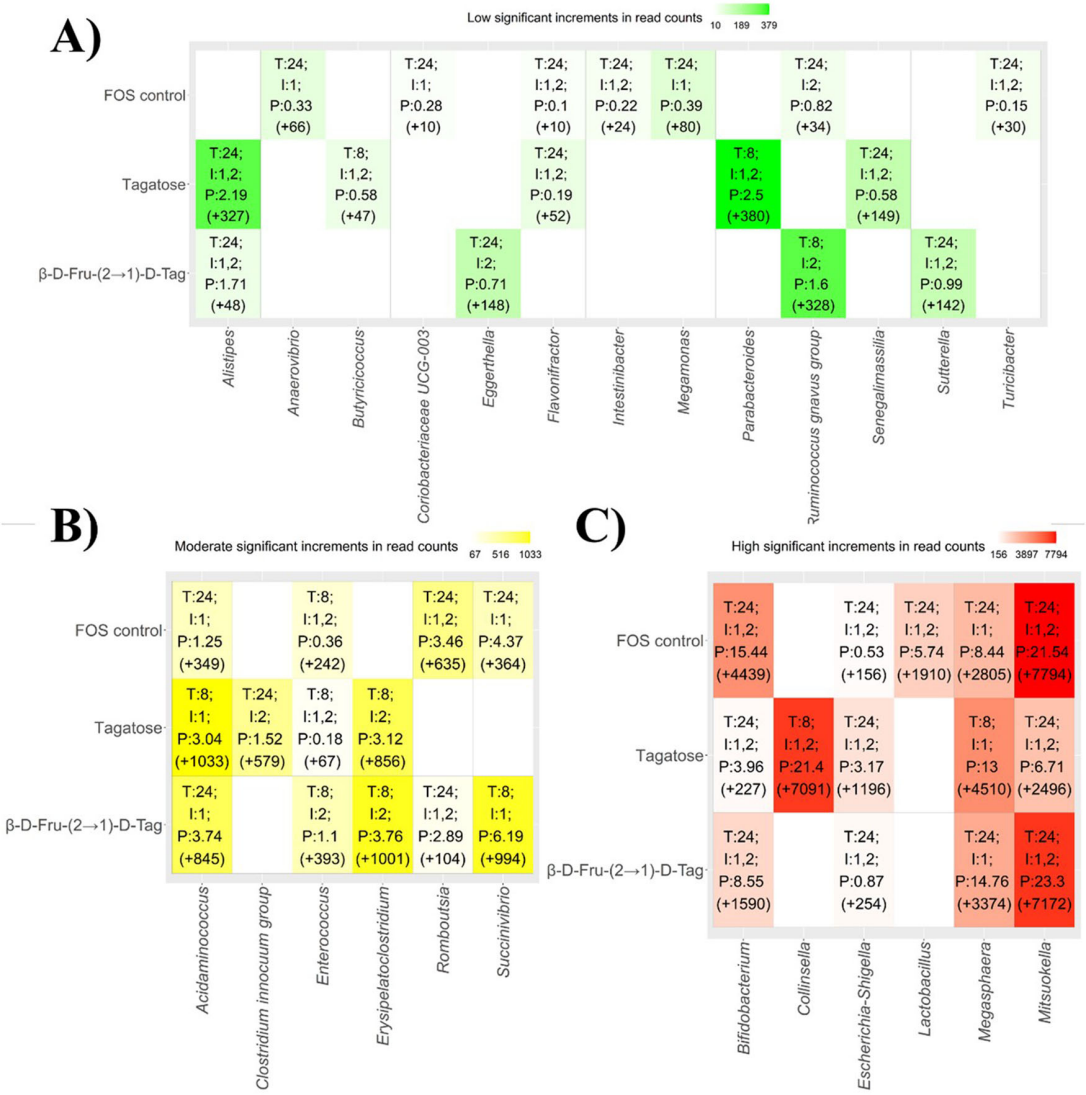
Statistically significant (adjusted p-values (padj) < 0.05) increments in bacterial genera after fecal fermentations of different substrates: fructo-oligosaccharides (FOS control), unmodified tagatose controls (tagatose), and fructosylated tagatose (β-D-Fru-(2 → 1)-D-Tag). These increments were classified into low (**A**), moderate (**B**), and high (**C**) increments compared to the initial fermentation time. Low increment values were comprised between zero and the second quartile. Similarly, moderate increment values were comprised between the second and the third quartiles. Finally, high increment values were comprised between the third and the fourth quartiles. T: fermentation time at which maximum increment of a specific genus was observed. I: individuals (donors) showing the maximum increment of a genus in their microbiota (it should be noted that most significant differences were observed in all individuals). P: abundance percentage of a specific genus showing a maximum increment at a given time. Bacterial counts increments are shown in parentheses.

To gain a better understanding of microbial taxa changes induced by each substrate (FOS and unmodified tagatose controls, and β-D-Fru-(2→1)-D-Tag), several differential abundance methods developed for microbiome studies (ANCOM, LEfSe, and metagenomeSeq) were computed. A total of 25 genera showed statistically significant increments (p < 0.05) in their read counts and abundances after 8 or 24 h of in vitro fecal fermentation. Microbial increments in read counts were classified as low (values between zero and the second quartile), moderate (values between the second and the third quartiles), and high (values between the third and the fourth quartiles) for comparative purposes (Fig. 5).

Concerning microbial genera showing low increments, commercial FOS selectively promoted the growth of *Anaerovibrio, Coriobacteriaceae* UCG-003, *Intestinibacter, Megamonas* and *Turicibacter* (Fig. 5A). Original tagatose control led to higher increments of *Alistipes, Butyricicoccus, Flavonifractor, Parabacteroides*, and *Senegalimassilia* compared to β-D-Fru-(2→1)-D-Tag. In contrast, β-D-Fru-(2→1)-D-Tag selectively stimulated *Eggerthella, Ruminococcus gnavus* group, and *Sutterella*.

The three substrates under study led to moderate increments of *Acidaminococcus* and *Enterococcus* while commercial FOS and β-D-Fru-(2→1)-D-Tag promoted the growth of *Romboutsia* and *Succinivibrio* (Fig. 5B). Similarly, FOS control and β-D-Fru-(2→1)-D-Tag led to higher increments of *Bifidobacterium* and *Mitsuokella* compared to the original tagatose control (Fig. 5C). In contrast, original tagatose exerted a higher modulatory effect on *Collinsella* and *Megasphaera* than the rest of substrates (Fig. 5C). Finally, FOS control selectively stimulated the growth of *Lactobacillus*. These findings underscore the similarities in the fermentative properties of commercial FOS mixtures and β-D-Fru-(2→1)-D-Tag.

**Fig. 6:**
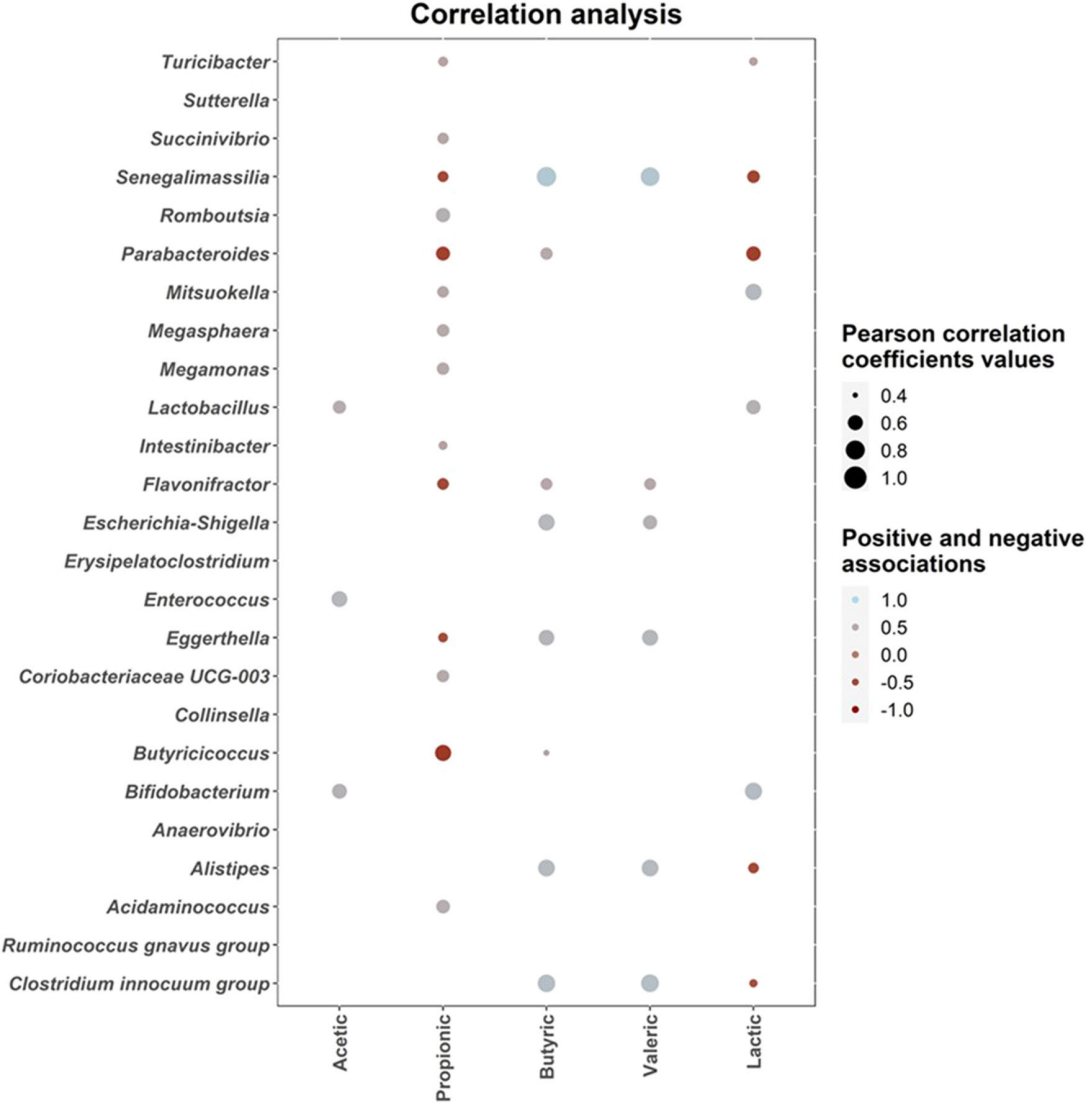
Correlation heatmaps showing the associations between short-chain fatty acids (SFCAs) concentrations (mM) and microbial genera exhibiting statistically significant increments during fecal fermentation of different substrates: fructo-oligosaccharides (FOS control), unmodified tagatose controls (tagatose), and fructosylated tagatose (β-D-Fru-(2 → 1)-D-Tag). Blue and red dots indicate positive and negative correlations expressed as Pearson correlation coefficients. Color intensity is in proportion to magnitude. Data represented correspond to all fermentation times (8 and 24 h). SCFAs determined include acetic, butyric, formic, isovaleric, lactic, propanoic, and valeric acids. Total SCFAs concentration is also represented.

Complementary correlation network analysis revealed a positive association between *Alistipes* and *Parabacteroides*, two genera showing higher increments after fecal fermentation of tagatose control compared to β-D-Fru-(2→1)-D-Tag. Similarly, negative associations between *Bifidobacterium*, showing high increments after FOS and β-D-Fru-(2→1)-D-Tag fermentation, and *Collinsella*, selectively promoted by original tagatose control, were found. Then, statistical associations between microbiota composition and lactate and SCFAs levels after fecal fermentation were determined (Fig. 6 and Figure S34).

Lactate and SCFAs determined in fecal fermentation samples include acetic acid (45.22 ± 31.19 mM, range: 0.56 – 109.04 mM), butyric acid (12.67 ± 14.48 mM, range: 0.16 – 54.46 mM), propionic acid (14.89 ± 12.52 mM, range: 0.28 – 42.83 mM), valeric acid (1.51 ± 3.07 mM, range 0.00 – 11.08 mM) and lactate (3.10 ± 2.53 mM, range 0.00 – 8.16 mM) (Figure S34). Correlation analysis revealed positive associations between *Alistipes, Clostridium inocuuum* group, *Eggerthella, Flavonifractor, Parabacteroides* and *Senegalimassilia*, and butyric and valeric acids levels (Fig. 6). Most of these taxa and *Succinivibrio* showed positive correlations with propionic acid levels. In addition, positive correlations between *Bifidobacterium, Lactobacillus*, and *Enterococcus* with acetic acid levels were found (Fig. 6). Similarly, *Bifidobacterium, Lactobacillus, Mitsuokella*, and *Turicibacter* abundances were positively correlated with lactate levels (Fig. 6). In general, genera showing significant (*p* < 0.05) correlations with SCFAs levels involve main SCFAs producers from human gut microbiota^47^. In addition, positive associations between *Lactobacillus, Turicibacter*, and *Succinivibrio* with several SCFAs including acetic, propanoic, and butyric acids have been reported^48^, in agreement with our results.

Several genera promoted by these substrates were also stimulated by other functional carbohydrate fractions in previous studies. In this regard, in vitro fermentation of bagasse fractions led to an increment in *Collinsella, Parabacteroides*, and *Senegalimassilia* while *Bifidobacterium* was selectively promoted by alkali-soluble arabinoxylo-oligosaccharide fractions^49^. Similarly, in vitro fermentation of apple pomace and pectin fractions resulted in high abundances of *Turicibacter*, a bacterium with possible anti-inflammatory effects^50^, *Lactobacillus* and *Succinivibrio*^48^.

It should be noted that β-D-Fru-(2→1)-D-Tag exerts a higher bifidogenic effect than unmodified tagatose, similar to the one observed for commercial FOS mixtures. *Bifidobacterium* species are widely recognized as probiotics that exert numerous benefits on human health including the production of SCFAs^51,52^, in agreement with our results. These differences in gut bacterial selectivity could be attributed to the fructosylation of tagatose via the glycosidic linkage β-(2-1) which is associated with well-described prebiotic properties. Structural modification of tagatose also resulted in a higher capacity to promote SCFAs-producing *Mitsuokella*^53^ compared to the original tagatose.

The ability of *Bifidobacterium* species to use tagatose as substrate has been investigated^54^. It has been reported that tagatose fermentation is common among lactic acid bacteria, although several *Bifidobacterium* may not be able to degrade tagatose^55^. Results here presented suggest that tagatose fructosylation may enhance its fermentative properties leading to a higher bifidogenic effect.

In conclusion, SacB, a levansucrase from *Bacillus subtilis* CECT 39, specifically transfers fructose moieties from sucrose to either C-1 of D-tagatose or C-5 of L-sorbose, forming the main disaccharides β-D-fructofuranosyl-(2 → 1)-D-tagatopyranose and β-D-fructofuranosyl-(2 → 5)-L-sorbopyranose, respectively. Molecular docking has provided a structural basis for understanding the catalytic mechanism of SacB and its preference for these rare sugars over other potential substrates like D-psicose. Additionally, the formation of rare sugar-based oligosaccharides with a higher degree of polymerization was demonstrated through the transfer of additional fructose residues to C-6 of either the β-2,1-linked fructose in D-tagatose or the β-2,5-linked fructose in L-sorbose, as determined by 2D-NMR. In vitro studies have shown the high resistance of the novel tagatose-based oligosaccharides to the hydrolytic action of the mucosal disaccharidases embedded in the pig small intestinal BBMV throughout the whole digestion time, thus supporting their potential role as low calorie ingredients. The lack of digestibility of the biosynthesized tagatose-based carbohydrates warranted further studies dealing with their fermentable properties and potential ability to selectively modulate the gut microbiota. Thus, β-D-fructofuranosyl-(2 → 1)-D-tagatopyranose exhibited distinct modulatory properties on the human fecal microbiota compared to unmodified tagatose, such as a higher bifidogenic effect, similar to that observed for well-known prebiotics like inulin-type FOS. Interestingly, the fructosylation of tagatose through the glycosidic linkage β-(2 → 1) selectively stimulated the growth of *Eggerthella*, *Ruminococcus gnavus* group, *Romboutsia, Succinivibrio*, *Sutterella* or SCFAs-producing *Mitsuokella* as compared to the original tagatose. Therefore, the bioconversion of D-tagatose into β-fructosylated species with a higher degree of polymerization is an efficient approach to increase the bioavailability of tagatose and promote its interaction with the gut microbiota. The approach described in this work opens new opportunities for tailoring natural rare sugars, such as D-tagatose and L-sorbose, to produce novel biosynthesized carbohydrates with functional and structural properties that are appealing for their use as emerging prebiotics and low-caloric sweeteners.

## Methods

### Chemicals, reagents, and carbohydrates

All chemicals and reagents used were of analytical grade and were purchased from Sigma-Aldrich (St. Louis, MO, USA), VWR (Barcelona, Spain), and Merck (Darmstadt, Germany). D-Tagatose was obtained from Carbosynth (Compton, UK). Ultrapure water, produced in-house using a laboratory water purification system (Milli-Q Synthesis A10, Millipore, Billerica, MA, U.S.A.), was used throughout the experiments.

### Production, purification, and activity assay of recombinant levansucrase SacB enzyme

Levansucrase SacB (EC 2.4.1.10) from *Bacillus subtilis* CECT 39 (ATCC 6051) was overproduced in *Escherichia coli* and purified following the method previously described by Díez-Municio, et al. ^30^. The total activity of SacB was determined by measuring the amount of free glucose released, while the hydrolytic (fructosidase) activity was assessed by quantifying the amount of formed fructose.

The transfructosylation activity (transferred fructose) was defined as the difference between the amounts of released glucose and fructose. Consequently, SacB exhibited a total specific activity of 20.77 units per milligram (U mg^−1^), where 1 unit is defined as the amount of enzyme that releases 1 μmol of glucose per minute at 37 °C in a sucrose concentration of 100 g L^−1^ at pH 6.0, using a 50 mM potassium phosphate buffer. The specific fructosidase activity was determined to be 10.79 U mg^−1^, where 1 unit is defined as the amount of enzyme that releases 1 μmol of fructose per minute under the same conditions. Finally, the transfructosylation activity was calculated to be 9.98 U mg^−1^, defined as the amount of enzyme required to transfer 1 μmol of fructose per minute to other molecules under the tested conditions^32^.

### Enzymatic synthesis of rare sugars-based oligosaccharides

The production of tagatose-based carbohydrates, using sucrose as the fructosyl donor and D-tagatose as the acceptor, was carried out through a transfructosylation reaction catalyzed by SacB. This reaction followed the conditions (i.e., pH 6.0 in 50 mM potassium phosphate buffer at 37 °C) previously optimized by Díez-Municio et al. ^30^ for synthesizing lactose-based fructosylated oligosaccharides. In this study, the SacB concentration was optimized by testing three different enzyme levels: 0.6, 3.1, and 6.2 U (transfructosylation activity) of SacB per mL of the reaction mixture. Samples were incubated in 2.0 mL individual tubes on an orbital shaker (Eppendorf Thermomixer Comfort, Hauppauge, NY, U.S.A.) at 1000 rpm. The reactions were allowed to proceed for up to 72 h, with aliquots taken at suitable time intervals (0.5, 1, 2, 4, 6, 8, 24, 48, and 72 h). The enzyme was inactivated by heating at 100 °C for 5 min, and the inactivated samples were then stored at −20 °C until analysis. Once the enzyme concentration was optimized, the production of tagatose-based oligosaccharides was studied at various sucrose to D-tagatose concentration ratios, i.e., 200:300, 300:300, 200:450, 200:200 g L^−1^. The primary parameters used to evaluate the efficiency of enzymatic synthesis for novel tagatose-based carbohydrates were: **i**) concentration (expressed as grams per liter of reaction); **ii**) yield (expressed as grams per 100 g of starting tagatose added); and **iii**) conversion (%) (expressed as the percentage of product concentration per concentration of starting sucrose).

Likewise, additional reactions based on D-sucrose:L-sorbose and D-sucrose:D-psicose were carried out under the same optimized conditions of pH, temperature, and levansucrase concentration, and at a concentration ratio of 200:200 g L^−1^. All synthesis reactions were performed in duplicate.

### Purification and isolation of D-tagatose- and L-sorbose-based oligosaccharides

Considering the lack of commercially available standards for rare sugar-based oligosaccharides, the main oligosaccharides synthesized after 24 h of the transfructosylation reaction based on D-sucrose:D-tagatose and D-sucrose:L-sorbose mixtures, catalyzed by levansucrase under the optimized conditions described earlier, were isolated and purified based on their degree of polymerization. This was achieved by preparative LC-RID on an Agilent Technologies 1260 Infinity LC System (Boeblingen, Germany), using a Zorbax NH_2_ PrepHT preparative column (250 × 21.2 mm, 7 μm particle size) (Agilent Technologies, Madrid, Spain). Two mL of reaction mixtures (200 mg of total carbohydrates) were eluted with acetonitrile:water (65:35, v:v) as the mobile phase at a flow rate of 21.0 mL/min for 30 min. The separated di- and trisaccharides were collected using an Agilent Technologies 1260 Infinity preparative-scale fraction collector (Boeblingen, Germany), and the fractions were pooled, evaporated in a rotary evaporator R-200 (Büchi Labortechnik AG, Flawil, Switzerland) below 25 °C, and freeze-dried. Three isolated fractions, labeled as T1, T2, and T3, and four fractions, labeled as S1, S2, S3, and S4, containing the purified fructosylated D-tagatose- and L-sorbose-based oligosaccharides, respectively, were prepared for subsequent NMR characterization. Likewise, purified T1 and T3 fractions were subjected to in vitro gastrointestinal digestibility studies, and the purified T1 fraction was also tested for its ability to modulate the human fecal microbiota in vitro.

### Structural characterization of biosynthesized rare sugars based-oligosaccharides by Nuclear Magnetic Resonance (NMR)

Structure elucidation of the purified fractions (T1-T3, S1-S4) was accomplished using Nuclear Magnetic Resonance spectroscopy (NMR). NMR spectra were recorded at 298 K, with D2O as the solvent, on an Agilent SYSTEM 500 NMR spectrometer (^1^H at 500 MHz, ^13^C at 125 MHz) equipped with a 5 mm HCN cold probe (25 K). Chemical shifts for ^1^H (δH) in parts per million were determined relative to solvent residual peak (HDO, δH 4.79) and for ^13^C (δC) were determined relative to external standard of 1,4-dioxane in D_2_O (δC 67.40). One-dimensional (1D) NMR experiments (^1^H and ^13^C{^1^H}) were performed using standard pulse sequences. ^1^H experiments were also acquired using PRESAT sequence for water peak suppression. Two-dimensional (2D) [^1^H, ^1^H] NMR experiments, including gradient correlation spectroscopy (gCOSY) and total correlation spectroscopy (TOCSY), were carried out with the following parameters: a delay time of 1 s, a spectral width of 2800 Hz in both dimensions, 2048 complex points in t2, 4 transients for each of 128 (200 for TOCSY) time increments, and linear prediction to 512. The data were zero-filled to 2048 × 2048 real points. 2D [^1^H − ^13^C] NMR experiments [gradient heteronuclear single-quantum coherence (gHSQC), hybrid experiment gHSQC-TOCSY, and gradient heteronuclear multiple bond correlation (gHMBC)] utilized the same ^1^H spectral window and a ^13^C spectral window of 7541.5 Hz, 1 s of relaxation delay, 1024 data points, and 128- or 200-time increments, with linear prediction to 256. The data were zero-filled to 2048 × 2048 real points. Typical numbers of transients per increment were 4 and 16. A mixing time of 80 ms was used for the gHSQC-TOCSY experiment.

### Quantitation of tagatose-based oligosaccharides in enzymatic mixtures by Gas Chromatography-Flame Ionization Detector (GC –FID)

The carbohydrates present in the reaction mixtures and their purified forms were analyzed by GC-FID as trimethylsilylated oximes (TMSO), prepared following the method reported by Brobst (1972)^56^. To form carbohydrate oximes, 250 μL of hydroxylamine chloride in pyridine (2.5% w/v) was added to dried samples, and the mixture was heated at 70 °C for 30 minutes. The resultant oximes were silylated using 250 μL of hexamethyldisilazane and 25 μL of trifluoroacetic acid at 50 °C for 30 min. The reaction mixtures were then centrifuged at 6700 × g for 2 min at room temperature. The supernatants were either injected into the GC-FID or stored at 4 °C prior to analysis. Carbohydrate separation was carried out using an Agilent Technologies gas chromatograph (Mod 7890 A) equipped with a flame ionization detector (FID) and a fused silica capillary column DB-5HT (5%-phenyl-methylpolysiloxane; 30 m × 0.25 mm × 0.10 μm) (Agilent), following the method described by Cardelle-Cobas, et al. ^57^. The oven temperature was initially set at 150 °C and then programmed to increase to 380 °C at a rate of 3 °C/min. The injector and detector temperatures were set at 280 °C and 385 °C, respectively. Injections were performed in split mode (1:20) using nitrogen at 1 mL min^−^^1^ as the carrier gas. Data acquisition and integration were managed using Agilent ChemStation software. Quantification was conducted using the internal standard method with phenyl-β-glucoside (0.5 mg mL^−1^) and the corresponding response factors from solutions containing glucose, fructose, sucrose, tagatose, sorbose, and raffinose (used as a trisaccharide standard). Carbohydrate concentration was expressed in grams per liter of reaction (g L^−1^). All analyses were carried out in duplicate, and the data were expressed as means ± standard deviation (SD).

### In silico molecular docking

Molecular docking was performed using the docking program AutoDock Vina^58^, with the crystal structure of *Bacillus subtilis* CECT 39 levansucrase SacB (PDB code: 1PT2) and the monosaccharides D-tagatose, L-sorbose, and D-psicose. The SacB structure was cleaned by removing water molecules and the ligand sucrose, and it was further prepared for docking by adding polar hydrogens and charges. Atomic coordinates for D-tagatose, L-sorbose, and D-psicose were sourced from the PubChem database, and their stereochemistry was revised using eLBOW from the Phenix package^42^. All PDB files were converted to the pdbqt format using AutoDockTools^59^. To verify the docking procedure in this particular system, the sucrose previously removed from the SacB structure was redocked using blind docking. A grid box search space was defined with dimensions of x = 30 Å, y = 30 Å, and z = 30 Å, and a search exhaustiveness of 32 was applied. The docked sucrose assumed the same pose as the crystallographic ligand, revealing the same set of molecular interactions, thus confirming the adequacy of the procedure. The molecular docking was conducted with the following parameters: a grid box search space with dimensions of x = 25 Å, y = 25 Å, and z = 25 Å, a search exhaustiveness of 32, and the ligand centered at x = 41.5 Å, y = 36.6 Å, and z = 13.0 Å. Evaluation of the best docked poses was carried out in consideration of the reported catalytic mechanism of SacB, particularly noting that the strictly conserved residues Asp86, Asp247, and Glu342 are required for catalysis^24^. PyMOL^60^ was utilized for the visualization of the structures, for analyzing the interactions, and for preparing figures.

### In vitro digestion of the main tagatose-based di- and trisaccharides using isolated pig small intestinal brush border membrane vesicles (BBMV)

Carbohydrates (4 mg) (purified fractions T1 and T3) in 1.5 mL of small intestinal fluid (SIF), the composition of which has been previously described by Brodkorb et al. ^61^. Briefly, SIF contains bile salts (10 mM), pancreatin (100 U/mL trypsin activity), calcium chloride (0.6 mM), and other salts. To accurately mimic the small intestinal digestive system, brush-border membrane vesicles (BBMV) from pig small intestine were isolated using the method described by Julio-Gonzalez et al. ^62^. Sixty milligrams of BBMV were added to the sample and incubated at 37 °C and 900 rpm in a Thermomixer^®^ (Eppendorf, Hamburg-Germany). Sample aliquots were taken after 1, 2, and 3 hours of hydrolysis and analyzed by GC-FID.

The main enzymatic activities of BBMV were estimated by individually dissolving lactose, maltose, sucrose, isomaltose, isomaltulose, and trehalose in PBS (0.2% w/v). One milliliter of each solution was transferred, in triplicate, to a 2 mL microtube containing 10 mg of BBMV. The mixture was then incubated at 37 °C and 900 rpm in a Thermomixer^®^ (Eppendorf, Hamburg-Germany). Fifty-microliter aliquots were taken every 30 min, up to 3 h of incubation. Each aliquot was subsequently incubated for 5 min in a water bath at 95 °C to inactivate the enzymatic mixture, and then centrifuged at 8000 × g (Mini Spin Eppendorf centrifuge, Hamburg, Germany). The supernatant was analyzed by GC-FID as previously described. The specific enzymatic activity (U g^−^^1^) was expressed as μmol min^−^^1^ g^−^^1^ of total protein, where one unit is defined as the amount of total protein present in BBMV required to hydrolyze 1 μmol of the corresponding disaccharide in one minute. The enzymatic activities were determined to be: β-galactosidase: 0.025 U mg^−^^1^; maltase: 0.448 U mg^−^^1^; sucrase: 0.035 U mg^−^^1^; trehalase: 0.033 U mg^−^^1^; isomaltase: 0.024 U mg^−^^1^; palatinase/isomaltulase: 0.024 U mg^−^^1^.

### In vitro fermentation properties of the main tagatose-based disaccharide

Ethics approval for fecal samples collection was obtained from the Bioethics Committee of CSIC (Consejo Superior de Investigaciones Científicas; no. 213/2020) and from the Regional Ethics Committee for Clinical Research (Servicio de Salud del Principado de Asturias; no. 2020.278). All donors provided written informed consent for their fecal matter to be used for the experiments.

Batch fecal fermentations of the purified tagatose-fructosylated disaccharide (T1 fraction), control (unmodified) tagatose, and commercial fructooligosaccharides (FOS) were carried out using two different fecal pools from healthy donors (n = 3 donors per pool). These pools were prepared by weighing equal amounts of fecal samples collected on the same day, which were then homogenized and used to inoculate the fermentations according to Calvete-Torre et al.^48^. The fecal batch fermentations were conducted in an MG500 anaerobic hood (Don Whitley Scientific, West Yorkshire, UK; atmosphere of 10% (v/v) H_2_, 10% CO_2_, and 80% N_2_) using the medium described by Sánchez-Patán, et al. ^63^. This medium was supplemented with the substrates to be tested (FOS, unmodified tagatose control, the tagatose-fructosylated disaccharide, and a control without carbon source) at a final concentration of 0.5% (w/v) as the sole carbon sources. All materials were pre-reduced for at least 24 h inside an anaerobic chamber before use. The fermentations were maintained in the anaerobic chamber with stirring at 37 °C, and samples were collected at 0, 8, and 24 h for DNA extraction and analysis of short-chain fatty acids (SCFAs).

### DNA extraction and high-throughput sequencing of 16S rRNA

DNA extraction was carried out using the Power Soil ProKit (Qiagen) with a few modifications, as previously described by Calvete-Torre et al. ^48^. Subsequently, partial 16S rRNA sequencing was performed on 12 samples, corresponding to fermentations with three different substrates (fructo-oligosaccharides (FOS), unmodified tagatose controls, and the tagatose-fructosylated disaccharide) and two different fecal pool inoculum, collected at various time points (0, 8, and 24 h). The sequencing and data analysis procedures were performed as previously described by Calvete-Torre et al. ^48^. Briefly, the V3–V4 region was sequenced using the primers 16S-ProV3V4-forward (CCTACGGGNBGCASCAG) and 16S-ProV3V4-reverse (GACTACNVGGGTATCTAATCC) on an Illumina MiSeq instrument at the Sequencing Facilities of the Instituto de Parasitología y Biomedicina “López Neyra”. The QIIME2 v.2021.8 suite^64^ was used for quality filtering of paired-end reads and taxonomic classification of Amplicon Variant Sequences (ASVs) using the SILVA 138 release reference database. The raw sequencing reads were deposited in the Sequence Read Archive (SRA), accession number PRJNA1075036.

Statistical analysis of the sequencing results was performed using R (v.4.2.2.) with packages specifically developed for microbiome studies. Alpha and beta-diversity estimators were calculated using the Phyloseq^65^ and Microbiome R packages^66^. Differential abundance analysis of samples (FOS, unmodified tagatose controls, and tagatose-fructosylated disaccharide) at different fermentation times (0, 8, and 24 h) was performed at the genus level using ANCOM, LEfSe, and metagenomeSeq methods implemented in the microbiomeMarker R package^67–71^. A complementary correlation network analysis was conducted using the ccrepe and qgraph R packages^72,73^ to investigate the associations of bacterial genera incremented after fecal fermentation. Similarly, Pearson correlation coefficients between genera promoted by each substrate and SCFAs levels were calculated using base R (v.4.2.2.) functions. Additional plots, including principal coordinate analysis (PCoA) and microbiome composition barplots, were computed using the Microbiome R package^66^ to provide a graphical representation of the sample distribution.

### Short-chain fatty acid (SCFA) analysis

SCFA concentrations during fecal fermentations were determined by GC-FID. To each 500 µL fecal sample, 25 µL of internal standard (IS) 2-ethyl butyric acid (0.1 M) was added. Organic acids were extracted following the method described by Muñoz-Labrador et al. ^74^, which involves adding 250 µL of concentrated HCl and 1 mL of diethyl ether, followed by mixing for 1 min. The samples were then centrifuged for 10 min at 2000 g. The organic phase was collected into a GC-capped vial, and 50 µL of N-(tert-butyldimethysilyl)-N-methyltrifluoroacetamine (MTBSTFA) was added for immediate derivatization at room temperature. The SCFA analyses were performed using an Agilent Technologies 7890 A gas chromatograph (Agilent Technologies, Wilmington, DE, USA) equipped with a flame ionization detector (FID) and an HTC column (30 m x 250 µm x 0.1 µm). Samples were injected in split mode (25:1). The temperatures of the injector and detector were set at 250 °C, and the oven thermal ramps were performed according to the method described by Ribeiro et al. ^75^.

## Supplementary information

Supplementary material

## Data Availability

We declare that all data related to this study are included in this paper and its supplementary information which includes additional NMR experimental details, NMR spectra for all compounds identified and data associated to microbiota modulation.
