# Supplementary material for: Tailoring the natural rare sugars D-tagatose and L-sorbose to produce novel functional carbohydrates
Source: NPJ Sci Food. 2024 Oct 4;8:74. doi: 10.1038/s41538-024-00320-8 (PMC11452612; doi:10.1038/s41538-024-00320-8)
Supplement: Supplementary file 1 — Supplementary material [file 41538_2024_320_MOESM1_ESM.pdf]

Supporting Information

Tailoring the natural rare sugars D-tagatose and L-sorbose to produce novel functional carbohydrates

Oswaldo Hernandez-Hernandez[1], Carlos Sabater[2,3], Inés Calvete-Torre[2,3], Elisa G. Doyagüez[4], Ana M. Muñoz-Labrador[1], Cristina Julio-Gonzalez[1], Blanca de las Rivas[5], Rosario Muñoz[5], Lorena Ruiz[2,3], Abelardo Margolles[2,3], José M. Mancheño[6], F. Javier Moreno[1*]

[1] Institute of Food Science Research, CIAL (CSIC-UAM), Nicolas Cabrera 9, 28049 Madrid, Spain

[2] Dairy Research Institute of Asturias (IPLA-CSIC), Paseo Río Linares s/n, 3300, Villaviciosa, Asturias, Spain

[3] Health Research Institute of Asturias (ISPA), Avenida Hospital Universitario s/n, 33011, Oviedo, Asturias, Spain

[4] Centro de Química Orgánica "Lora Tamayo" (CSIC), Juan de la Cierva 3, 28006, Madrid, Spain

[5] Institute of Food Science, Technology and Nutrition, ICTAN (CSIC), Juan de la Cierva 3, 28006 Madrid, Spain

[6] Institute of Physical Chemistry 'Blas Cabrera' (IQF-CSIC), Serrano 119, 28006 Madrid, Spain

* Corresponding author: javier.moreno@csic.es

EXPERIMENTAL DETAILS.

NMR Experiments

β-D-fructofuranosyl-(2→1)-α-D-tagatopyranose (1α):
^{1}H-NMR (D_2O, 500 MHz) δ 3.60 (H6A), 3.66 (H1A, H1B, H1'A), 3.69 (H6'A), 3.73 (H1'B, H6B), 3.79 (H6'B), 3.83 (H4, H5), 3.87 (H3, H5'), 4.11 (H4'), 4.18 (H3').
^{13}C{^{1}H}-NMR (D_2O, 100 MHz) δ: 60.61 (C1'), 62.92 (C6, C6'), 64.63 (C1), 66.95 (C5), 70.83 (C3), 71.36 (C4), 74.96 (C4'), 77.61 (C3'), 81.82 (C5'), 98.53 (C2), 104.19 (C2').

β-D-fructofuranosyl-(2→1)-β-D-tagatopyranose (1β):
^{1}H-NMR (D_2O, 500 MHz) δ 3.57 (H1A), 3.65 (H1'A, H6A, H6B), 3.67 (H6'A), 3.73 (H1'B), 3.80 (H6'B), 3.86 (H5'), 3.89 (H5), 3.90 (H3), 3.99 (H4), 4.10 (H4'), 4.17 (H1B), 4.18 (H3').
^{13}C{^{1}H}-NMR (D_2O, 100 MHz) δ: 60.78 (C1), 60.80 (C1'), 62.68 (C6'), 64.08 (C6), 64.63 (C5), 69.86 (C3), 71.45 (C4), 74.85 (C4'), 77.61 (C3'), 81.80 (C5'), 98.75 (C2), 104.25 (C2').

β-D-fructofuranosyl-(2→6)-α-D-glucopyranose (2α):
^{1}H-NMR (D_2O, 500 MHz) δ 3.42 (H4), 3.52 (H2), 3.67 (H1'A, H6'A), 3.69 (H3), 3.70 (H6A), 3.74 (H1'B), 3.80 (H6'B), 3.85 (H5'), 3.90 (H5), 3.97 (H6B), 4.10 (H4'), 4.16 (H3'), 5.21 (1H, d, ^3J = 3.9 Hz, H1)
^{13}C{^{1}H}-NMR (D_2O, 100 MHz) δ: 60.78 (C1'), 61.39 (C6), 63.01 (C6'), 70.35 (C4), 71.31 (C5), 72.08 (C2), 73.29 (C3), 75.09 (C4'), 77.45 (C3'), 81.79 (C5'), 92.76 (C1), 104.34 (C2').

β-D-fructofuranosyl-(2→6)-β-D-glucopyranose (2β):
^{1}H-NMR (D_2O, 500 MHz) δ 3.24 (H2), 3.42 (H4), 3.46 (H3), 3.53 (H5), 3.67 (H1'A, H6'A), 3.70 (H6A), 3.74 (H1'B), 3.80 (H6'B), 3.85 (H5'), 4.00 (H6B), 4.10 (H4'), 4.16 (H3'), 4.63 (1H, d, ^3J = 8.0 Hz, H1)
^{13}C{^{1}H}-NMR (D_2O, 100 MHz) δ: 60.75 (C1'), 61.41 (C6), 63.13 (C6'), 70.29 (C4), 74.70 (C2), 75.24 (C4'), 75.61 (C5), 76.26 (C3), 77.55 (C3'), 81.84 (C5'), 96.61 (C1), 104.37 (C2').

β-D-fructofuranosyl-(2→6)-β-D-fructofuranosyl-(2→1)-α-D-tagatopyranose (3α):

^{1}H-NMR (D_2O, 500 MHz) δ 3.62 (H6A), 3.63 (H6''A, H6''B), 3.66 (H1A, H1'A, H1''A, H1B), 3.69 (H6'A), 3.75 (H1'B), 3.77 (H6B), 3.78 (H1''B), 3.84 (H4), 3.85 (H5), 3.86 (H5''), 3.89 (H3), 3.92 (H6'B), 3.94 (H5'), 4.10 (H4'), 4.11 (H4''), 4.17 (H3', H3'').
^{13}C{^{1}H}-NMR (D_2O, 100 MHz) δ: 60.61 (C1'), 60.76 (C1''), 62.94 (C6), 63.06 (C6''), 63.58 (C6'), 64.71 (C1), 66.96 (C5), 70.73 (C3), 71.38 (C4), 75.21 (C4''), 75.76 (C4'), 77.26 (C3''), 77.43 (C3'), 80.79 (C5'), 81.77 (C5''), 98.59 (C2), 104.36 (C2''), 104.51 (C2').

β-D-fructofuranosyl-(2→6)-β-D-fructofuranosyl-(2→1)-β-D-tagatopyranose (3β):

^{1}H-NMR (D_2O, 500 MHz) δ 3.59 (H1A), 3.63 (H6''A, H6''B), 3.65 (H6A, H6B), 3.66 (H1''A), 3.69 (H6'A), 3.78 (H1''B), 3.86 (H5''), 3.92 (H3, H5, H6'B), 3.94 (H5'), 4.01 (H4), 4.08 (H4'), 4.11 (H4''), 4.17 (H3''), 4.20 (H1B).
^{13}C{^{1}H}-NMR (D_2O, 100 MHz) δ: 60.48 (C1'), 60.70 (C1), 60.76 (C1''), 63.06 (C6''), 63.53 (C6'), 63.98 (C6), 64.47 (C5), 69.92 (C3), 71.52 (C4), 75.21 (C4''), 75.64 (C4'), 77.26 (C3''), 77.32 (C3'), 80.86 (C5'), 81.77 (C5''), 98.82 (C2), 104.36 (C2''), 104.53 (C2').

β-D-fructofuranosyl-(2→6)-α-D-glucopyranosyl-(1→2)-β-D-fructofuranoside (4):

^{1}H-NMR (D$_2$O, 500 MHz) δ 3.46 (H4’), 3.53 (H2’), 3.66 (H1’’$_A$), 3.67 (H6’’$_A$), 3.71 (H1$_A$), 3.74 (H3’), 3.75 (H1’’$_B$), 3.80 (H6$_A$, H6$_B$, H6’’$_B$), 3.81 (H1$_B$), 3.83 (H5’), 3.86 (H5, H5’’), 3.92 (H6’$_A$), 4.02 (H6’$_B$), 4.04 (H4), 4.08 (H4’’), 4.18 (H3’’), 4.27 (H3), 5.43 (1H, d, ^{3}J = 3.9 Hz, H1’)
^{13}C{^{1}H}-NMR (D$_2$O, 100 MHz) δ: 60.70 (C6’), 61.04 (C1’’), 61.53 (C1), 62.82 (C6), 63.09 (C6’’), 69.85 (C4’), 71.77 (C2’), 73.06 (C5’), 73.22 (C3’), 74.48 (C4), 75.10 (C4’’), 77.23 (C3), 77.47 (C3’’), 81.77 (C5’’), 81.85 (C5), 93.13 (C1’), 103.89 (C2), 104.44 (C2’’).

β-D-fructofuranosyl-(2→5)-α-L-sorbopyranose (6):

^{1}H-NMR (D$_2$O, 500 MHz) δ 3.51 (H1$_A$), 3.54 (H3), 3.67 (H6’$_A$, H6’$_B$), 3.69 (H1$_B$), 3.73 (H4), 3.77 (H1’$_A$, H1’$_B$), 3.82 (H5, H5’), 3.92 (H6$_A$, H6$_B$), 4.00 (H4’), 4.17 (H3’).
^{13}C{^{1}H}-NMR (D$_2$O, 100 MHz) δ: 61.48 (C1’), 62.18 (C6), 63.13 (C6’), 64.04 (C1), 70.77 (C3), 71.41 (C5), 72.95 (C4), 74.97 (C4’), 77.58 (C3’), 81.42 (C5’), 98.10 (C2), 104.15 (C2’).

β-D-fructofuranosyl-(2→6)-β-D-fructofuranosyl-(2→5)-α-L-sorbopyranose (7):

^{1}H-NMR (D$_2$O, 500 MHz) δ 3.51 (H1$_A$), 3.54 (H3), 3.61 (H6’$_A$), 3.66 (H6$_A$, H6’’$_A$), 3.67 (H1’’$_A$), 3.69 (H1$_B$), 3.74 (H4), 3.76 (H1’$_A$, H1’$_B$), 3.79 (H1’’$_B$, H5), 3.82 (H6’’$_B$), 3.86 (H5’’), 3.88 (H6$_B$), 3.91 (H5’), 3.97 (H6’$_B$), 4.02 (H4’), 4.10 (H4’’), 4.17 (H3’, H3’’).
^{13}C{^{1}H}-NMR (D$_2$O, 100 MHz) δ: 60.79 (C1’’), 61.22 (C1’), 62.20 (C6), 63.02 (C6’’), 63.71 (C6’), 64.04 (C1), 70.73 (C3), 71.64 (C5), 72.89 (C4), 75.17 (C4’’), 75.49 (C4’), 77.14 (C3’’), 77.48 (C3’), 80.62 (C5’), 81.73 (C5’’), 98.11 (C2), 104.47 (C2’’), 104.53 (C2’).

β-D-fructofuranosyl-(2→3)-α-D-glucopyranosyl-(1→2)-β-D-fructofuranoside (8):

^{1}H-NMR (D$_2$O, 500 MHz) δ 3.47 (H4’), 3.55 (H2’), 3.80 (H6’’$_A$, H6’’$_B$), 3.83 (H5’), 3.86 (H5), 3.88 (H5’’), 3.94 (H3’), 3.96 (H1$_A$), 4.04 (H4’’), 4.19 (H3’’), 5.40 (1H, d, ^{3}J = 3.9 Hz, H1’)
^{13}C{^{1}H}-NMR (D$_2$O, 100 MHz) δ: 60.72 (C6’), 60.96 (C1), 69.81 (C4’), 71.64 (C2’), 72.17 (C3’), 73.06 (C5’), 81.77 (C5), 81.98 (C5’’), 92.64 (C1’), 104.28 (C2 or C2’’), 104.36 (C2 or C2’’).

β-D-fructofuranosyl-(2→6)-β-D-fructofuranosyl-(2→6)-β-D-fructofuranosyl-(2→5)-α-L-sorbopyranose (9):
^{1}H-NMR (D$_2$O, 500 MHz) δ 3.51 (H1$_A$), 3.54 (H3), 3.56 (H6’’$_A$), 3.62 (H6’$_A$), 3.64 (H6’’’$_A$), 3.65 (H1’’’$_A$), 3.66 (H6$_A$), 3.69 (H1$_B$), 3.74 (H4), 3.77 (H1’$_A$, H1’$_B$, H1’’$_A$, H1’’$_B$), 3.79 (H5), 3.81 (H1’’’$_B$, H6’’’$_B$), 3.86 (H5’’’, H6$_B$), 3.91 (H5’, H6’$_B$), 3.93 (H6’’$_B$), 3.94 (H5’’), 4.02 (H4’), 4.10 (H4’’), 4.11 (H4’’’), 4.17 (H3’’’), 4.18 (H3’, H3’’).
^{13}C{^{1}H}-NMR (D$_2$O, 100 MHz) δ: 60.68 (C1’’’), 61.23 (C1’, C1’’), 62.22 (C6), 63.10 (C6’’’), 63.74 (C6’), 63.95 (C6’’), 64.05 (C1), 70.75 (C3), 71.63 (C5), 72.89 (C4), 75.21 (C4’’’), 75.44 (C4’), 75.95 (C4’’), 77.07 (C3’’), 77.08 (C3’’’), 77.41 (C3’), 80.56 (C5’), 80.86 (C5’’), 81.80 (C5’’’), 98.10 (C2), 104.45 (C2’’’), 104.53 (C2’), 104.84 (C2’’).

β-D-fructofuranosyl-(2→6)-β-D-fructofuranosyl-(2→6)-α-D-glucopyranosyl-(1→2)-β-D-fructofuranoside (10):

^{1}H-NMR (D_2O, 500 MHz) δ 3.47 (H4’), 3.54 (H2’), 3.64 (H6’’$_A$, H6’’’$_A$), 3.66 (H1’’$_A$), 3.67 (H1$_A$, H1’’’$_A$, H6’$_A$), 3.70 (H6’$_B$), 3.74 (H3’), 3.76 (H1’’$_B$), 3.77 (H1$_B$, H1’’’$_B$), 3.79 (H6$_A$), 3.81 (H6’’$_B$, H6’’’$_B$), 3.83 (H6$_B$, H5’), 3.86 (H5’’’), 3.88 (H5), 3.94 (H5’’), 4.04 (H4’’), 4.05 (H4), 4.11 (H4’’’), 4.17 (H3’’, H3’’’), 4.26 (H3), 5.42 (1H, d, ^3J = 3.9 Hz, H1’)
^{13}C{^{1}H}-NMR (D_2O, 100 MHz) δ: 60.58 (C1, C1’’’), 60.99 (C6’), 61.41 (C1’’), 62.72 (C6), 63.10 (C6’’’), 63.16 (C6’’), 69.81 (C4’), 71.22 (C2’), 73.06 (C5’), 73.22 (C3’), 74.42 (C4), 75.28 (C4’’’), 75.79 (C4’’), 77.07 (C3, C3’’), 77.19 (C3’’’), 80.87 (C5’’), 81.80 (C5’’’), 81.86 (C5), 93.15 (C1’), 103.89 (C2), 104.45 (C2’’’), 104.65 (C2’’).

β-D-fructofuranosyl-(2→6)-β-D-fructofuranosyl-(2→6)-β-D-fructofuranosyl-(2→6)-β-D-fructofuranosyl-(2→5)-α-L-sorbopyranose (11).

^{1}H-NMR (D_2O, 500 MHz) δ 3.51 (H1$_A$), 3.54 (H3), 3.56 (H6’’$_A$, H6’’’$_A$), 3.62 (H6’$_A$), 3.64 (H6’’’’$_A$), 3.66 (H1’’’’$_A$, H6$_A$), 3.69 (H1$_B$), 3.74 (H4), 3.77 (H1’$_A$, H1’$_B$, H1’’$_A$, H1’’$_B$, H1’’’$_A$, H1’’’$_B$, H1’’’’$_B$), 3.79 (H5), 3.81 (H6’’’’$_B$), 3.86 (H5’’’’, H6$_B$), 3.91 (H5’, H6’$_B$), 3.93 (H6’’$_B$, H6’’’$_B$), 3.94 (H5’’, H5’’’), 4.02 (H4’), 4.10 (H4’’, H4’’’), 4.11 (H4’’’’), 4.18 (H3’, H3’’, H3’’’, H3’’’’).
^{13}C{^{1}H}-NMR (D_2O, 100 MHz) δ: 60.61 (C1’’’’), 61.23 (C1’, C1’’, C1’’’), 62.15 (C6), 63.09 (C6’’’’), 63.99 (C1, C6’, C6’’, C6’’’), 70.74 (C3), 71.62 (C5), 72.89 (C4), 75.22 (C4’’’’), 75.39 (C4’), 75.99 (C4’’, C4’’’), 76.96 (C3’’’’), 77.09 (C3’’, C3’’’), 77.42 (C3’), 80.56 (C5’), 80.83 (C5’’), 80.94 (C5’’’), 81.76 (C5’’’’), 98.10 (C2), 104.45 (C2’’’’), 104.54 (C2’), 104.84 (C2’’, C2’’’).

Table S1. ^{1}H (500 MHz) and ^{13}C (125 MHz) NMR chemical shifts (δ, ppm) determined by 1D and 2D NMR spectroscopy of disaccharides **1α** and **1β**.

		β-D -Fru*f* (2→1)–α -D-Tag*p* (**1α**)		β-D -Fru*f* (2→1)– β -D-Tag*p* (**1β**)	
		δ_C	δ_H	δ_C	δ_H
Tag	1a	64.63	3.66	60.78	3.57
	1b				4.17
	2	98.53		98.75	
	3	70.83	3.87	69.86	3.90
	4	71.36	3.83	71.45	3.99
	5	66.95	3.83	64.63	3.89
	6a	62.92	3.60	64.08	3.65
	6b		3.73		
Fru	1a	60.61	3.66	60.80	3.65
	1b		3.73		3.73
	2	104.19		104.25	
	3	77.61	4.18	77.61	4.18
	4	74.96	4.11	74.85	4.10
	5	81.82	3.87	81.80	3.86
	6a	62.92	3.69	62.68	3.67
	6b		3.79		3.80

Table S2. ^{1}H (500 MHz) and ^{13}C (125 MHz) NMR chemical shifts (δ, ppm) and coupling constants (*J* in Hz, in parentheses) determined by 1D and 2D NMR spectroscopy of disaccharides **2α** and **2β**.

		β-D -Fru*f* (2→6)–β-D-Glc*p* (**2β**)		β-D -Fru*f* (2→6)–α-D-Glc*p* (**2α**)	
		δ_C	δ_H (*J*, Hz)	δ_C	δ_H(*J*, Hz)
Glc	1	96.61	4.63 (8.0)	92.76	5.21 (3.9)
	2	74.70	3.24	72.08	3.52
	3	76.26	3.46	73.29	3.69
	4	70.29	3.42	70.35	3.42
	5	75.61	3.53	71.31	3.90
	6a	61.41	3.70	61.39	3.70
	6b		4.00		3.97
Fru	1a	60.75	3.67	60.78	3.67
	1b		3.74		3.74
	2	104.37		104.34	
	3	77.55	4.16	77.45	4.16
	4	75.24	4.10	75.09	4.10
	5	81.84	3.85	81.79	3.85
	6a	63.13	3.67	63.01	3.67
	6b		3.80		3.80

Table S3. ^{1}H (500 MHz) and ^{13}C (125 MHz) NMR chemical shifts (δ, ppm) and coupling constants (*J* in Hz, in parentheses) determined by 1D and 2D NMR spectroscopy of trisaccharides **3α**, **3β** and **4**.

		β-D -Fru*f* (2→6)– β-D -Fru*f* (2→1)-α -D-Tag*p* (**3α**)		β-D -Fru*f* (2→6)– β-D -Fru*f* (2→1)- β -D-Tag*p* (**3β**)		β-D -Fru*f* (2→6)–α-D-Glc*p*(1→2)– β-D –Fru*f* (**4**)	
		δ_C	δ_H (*J*, Hz)	δ_C	δ_H(*J*, Hz)	δ_C	δ_H(*J*, Hz)
Tag/Glc	1a	64.71	3.66	60.70	3.59	93.13	5.43 (3.9)
	1b				4.20		
	2	98.59		98.82		71.77	3.53
	3	70.73	3.89	69.92	3.92	73.22	3.74
	4	71.38	3.84	71.52	4.01	69.85	3.46
	5	66.96	3.85	64.47	3.92	73.06	3.83
	6a	62.94	3.62	63.98	3.65	60.70	3.92
	6b		3.77				4.02
Fru^I	1a	60.61	3.66	60.48		61.53	3.71
	1b		3.75				3.81
	2	104.51		104.53		103.89	
	3	77.43	4.17	77.32		77.23	4.27
	4	75.76	4.10	75.64	4.08	74.48	4.04
	5	80.79	3.94	80.86	3.94	81.85	3.86
	6a	63.58	3.69	63.53	3.69	62.82	3.80
	6b		3.92		3.92		
Fru^II	1a	60.76	3.66	60.76	3.66	61.04	3.66
	1b		3.78		3.78		3.75
	2	104.36		104.36		104.44	
	3	77.26	4.17	77.26	4.17	77.47	4.18
	4	75.21	4.11	75.21	4.11	75.10	4.08
	5	81.77	3.86	81.77	3.86	81.77	3.86
	6a	63.06	3.63	63.06	3.63	63.09	3.67
	6b						3.80

Table S4. ^{1}H (500 MHz) and ^{13}C (125 MHz) NMR chemical shifts (δ, ppm) determined by 1D and 2D NMR spectroscopy of compounds **6**, **7**, **9**, and **11**.

		β-D -Fru*f* -(2→5)- α -L-Sor*p* (**6**)		β-D -Fru*f* (2→6)- β-D -Fru*f* -(2→5)- α -L-Sor*p* (**7**)		[β-D -Fru*f* (2→6)]$_2$- β-D -Fru*f* -(2→5)- α -L-Sor*p* (**9**)		[β-D -Fru*f* (2→6)]$_3$- β-D -Fru*f* -(2→5)- α -L-Sor*p* (**11**)	
		δ_C	δ_H	δ_C	δ_H	δ_C	δ_H	δ_C	δ_H
Sor	1a	64.04	3.51	64.04	3.51	64.05	3.51	63.99	3.51
	1b		3.69		3.69		3.69		3.69
	2	98.10		98.11		98.10		98.10	
	3	70.77	3.54	70.73	3.54	70.75	3.54	70.74	3.54
	4	72.95	3.73	72.89	3.74	72.89	3.74	72.89	3.74
	5	71.41	3.82	71.64	3.79	71.63	3.79	71.62	3.79
	6a	62.18	3.92	62.20	3.66	62.22	3.66	62.15	3.66
	6b				3.88		3.86		3.86
Fru^{I}	1a	61.48	3.77	61.22	3.76	61.23	3.77	61.23	3.77
	1b								
	2	104.15		104.53		104.53		104.54	
	3	77.58	4.17	77.48	4.17	77.41	4.18	77.42	4.18
	4	74.97	4.00	75.49	4.02	75.44	4.02	75.39	4.02
	5	81.42	3.82	80.62	3.91	80.56	3.91	80.56	3.91
	6a	63.13	3.67	63.71	3.61	63.74	3.62	63.99	3.62
	6b				3.97		3.91		3.91
Fru^{II}	1a			60.79	3.67	61.23	3.77	61.23	3.77
	1b				3.79				
	2			104.47		104.84		104.84	
	3			77.14	4.17	77.07	4.18	77.09	4.18
	4			75.17	4.10	75.95	4.10	75.99	4.10
	5			81.73	3.86	80.86	3.94	80.83	3.94
	6a			63.02	3.66	63.95	3.56	63.99	3.56
	6b				3.82		3.93		3.93
Fru^{III}	1a					60.68	3.65	61.23	3.77
	1b						3.81		
	2					104.45		104.84	
	3					77.08	4.17	77.09	4.18
	4					75.21	4.11	75.99	4.10
	5					81.80	3.86	80.94	3.94
	6a					63.10	3.64	63.99	3.56
	6b						3.81		3.93
Fru^{IV}	1a							60.61	3.66
	1b								3.77
	2							104.45	
	3							76.96	4.18
	4							75.22	4.11
	5							81.76	3.86
	6a							63.09	3.64
	6b								3.81

Table S5. ^{1}H (500 MHz) and ^{13}C (125 MHz) NMR chemical shifts (δ, ppm) and coupling constants (*J* in Hz, in parentheses) determined by 1D and 2D NMR spectroscopy of trisaccharide **8**.

		β-D -Fru*f* (2→3)–α-D-Glc*p*(1→2)– β-D –Fru*f* (**8**)	
		δ_C	δ_H (*J*, Hz)
Glc	1a	92.64	5.40(3.9)
	1b		
	2	71.64	3.55
	3	72.17	3.94
	4	69.81	3.47
	5	73.06	3.83
	6a	60.72	n.i.
	6b		n.i.
Fru^{I}	1a	60.96	3.96
	1b		
	2	104.28-104.36	
	3	n.i.	n.i.
	4	n.i.	n.i.
	5	81.77	3.86
	6a	n.i.	n.i.
	6b		
Fru^{II}	1a	n.i.	n.i.
	1b		
	2	104.28-104.36	
	3	n.i.	4.19
	4	n.i.	4.04
	5	81.98	3.88
	6a	n.i.	3.80
	6b		

Table S6. ^{1}H (500 MHz) and ^{13}C (125 MHz) NMR chemical shifts (δ, ppm) and coupling constants (*J* in Hz, in parentheses) determined by 1D and 2D NMR spectroscopy of trisaccharide **4** and tetrasaccharide **10**.

		β-D -Fru*f* (2→6)–α-D-Glc*p*(1→2)– β-D –Fru*f* (**4**)		β-D -Fru*f* (2→6)–β-D -Fru*f* (2→6)–α-D-Glc*p*(1→2)– β-D –Fru*f* (**10**)	
		δ_C	δ_H(*J*, Hz)	δ_C	δ_H(*J*, Hz)
Glc	1a	93.13	5.43 (3.9)	93.15	5.42(3.9)
	1b				
	2	71.77	3.53	71.22	3.54
	3	73.22	3.74	73.22	3.74
	4	69.85	3.46	69.81	3.47
	5	73.06	3.83	73.06	3.83
	6a	60.70	3.92	60.99	3.67
	6b		4.02		3.70
Fru^{I}	1a	61.53	3.71	60.58	3.67
	1b		3.81		3.77
	2	103.89		103.89	
	3	77.23	4.27	77.07	4.26
	4	74.48	4.04	74.42	4.05
	5	81.85	3.86	81.86	3.88
	6a	62.82	3.80	62.72	3.79
	6b				3.83
Fru^{II}	1a	61.04	3.66	61.41	3.66
	1b		3.75		3.76
	2	104.44		104.65	
	3	77.47	4.18	77.07	4.17
	4	75.10	4.08	75.79	4.04
	5	81.77	3.86	80.87	3.94
	6a	63.09	3.67	63.16	3.64
	6b		3.80		3.81
Fru^{III}	1a			60.58	3.67
	1b				3.77
	2			104.45	
	3			77.19	4.17
	4			75.28	4.11
	5			81.80	3.86
	6a			63.10	3.64
	6b				3.81

Table S7. Comprehensive list of structures elucidated within this study.

Donor:Acceptor	STRUCTURES
Sucrose:Tagatose	β-D-fructofuranosyl-(2→1)-α-D-tagatopyranose
	β-D-fructofuranosyl-(2→1)-β-D-tagatopyranose
	β-D-fructofuranosyl-(2→6)-β-D-glucopyranose
	β-D-fructofuranosyl-(2→6)-α-D-glucopyranose
	β-D-fructofuranosyl-(2→6)- β-D-fructofuranosyl-(2→1)-α-D-tagatopyranose
	β-D-fructofuranosyl-(2→6)-β-D-fructofuranosyl-(2→1)-β-D-tagatopyranose
	β-D-fructofuranosyl-(2→6)-α-D-glucopyranosyl-(1→2)-β-D-fructofuranoside
	β-D-fructofuranosyl-(2→6)-β-D-fructofuranosyl-(2→6)-β-D-glucopyranose
	β-D-fructofuranosyl-(2→6)-β-D-fructofuranosyl-(2→6)-α-D-glucopyranose
**Donor:Acceptor**	**STRUCTURES**
Sucrose:Sorbose	β-D-fructofuranosyl-(2→5)-α-L-sorbopyranose
	β-D-fructofuranosyl-(2→6)-β-D-fructofuranosyl-(2→5)-α-L-sorbopyranose.
	β-D-fructofuranosyl-(2→3)-α-D-glucopyranosyl-(1→2)-β-D-fructofuranoside.
	β-D-fructofuranosyl-(2→6)-β-D-fructofuranosyl-(2→6)-β-D-fructofuranosyl-(2→5)-α-L-sorbopyranose
	β-D-fructofuranosyl-(2→6)-β--D-fructofuranosyl-(2→6)-α-D-glucopyranosyl-(1→2)-β-D-fructofuranoside
	β-D-fructofuranosyl-(2→6)-β-D-fructofuranosyl-(2→6)-β-D-fructofuranosyl-(2→6)-β-D-fructofuranosyl-(2→5)-α-L-sorbopyranose

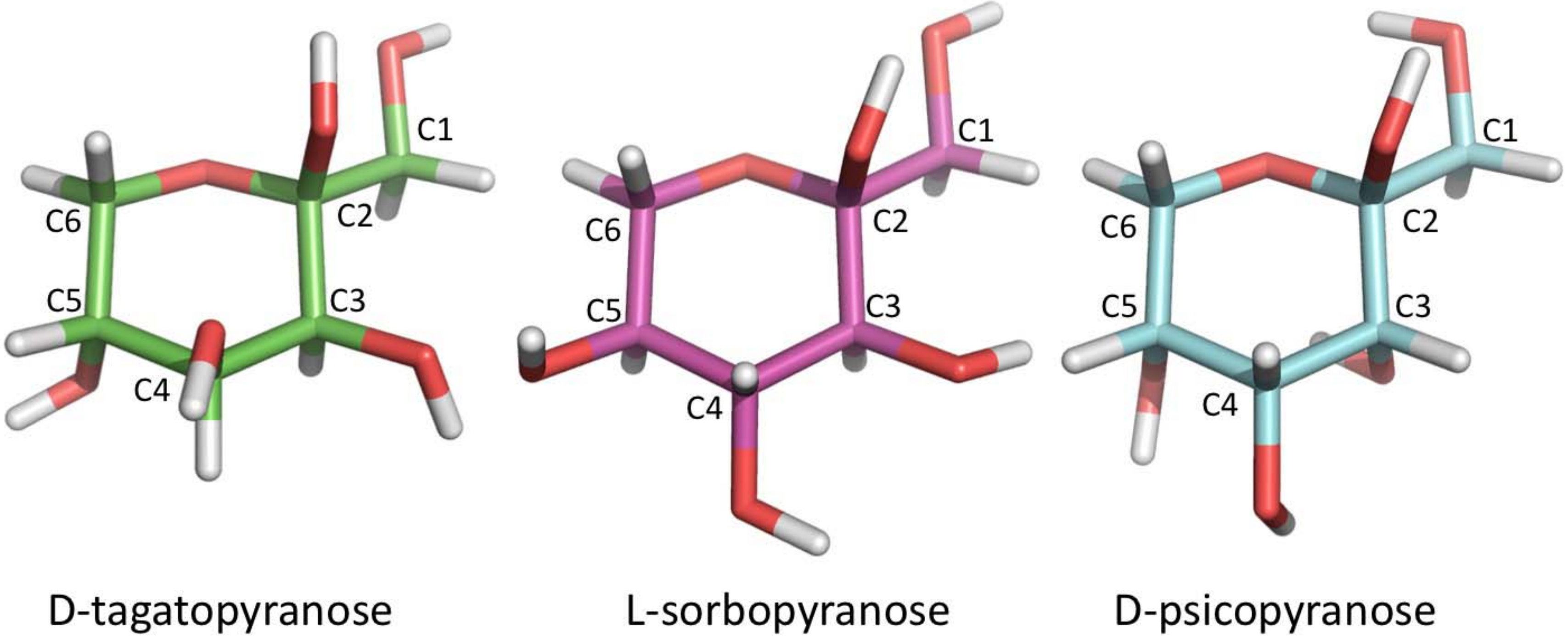

Figure S1. Structure of the ketohexoses used in the study.

NMR spectra:

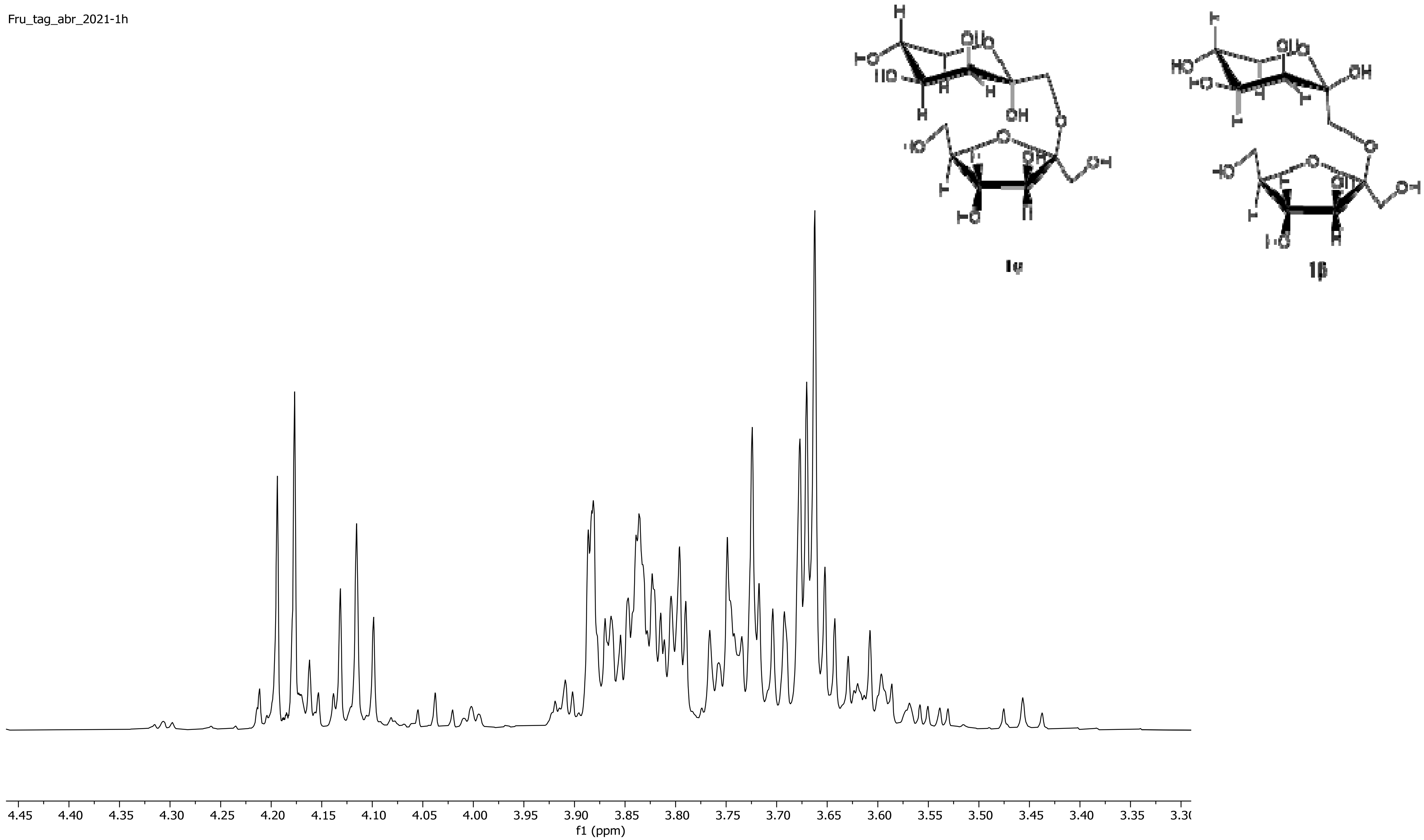

Figure S2. ^{1}H NMR (500 MHz, D_2O) of disaccharides **1α** and **1β**.

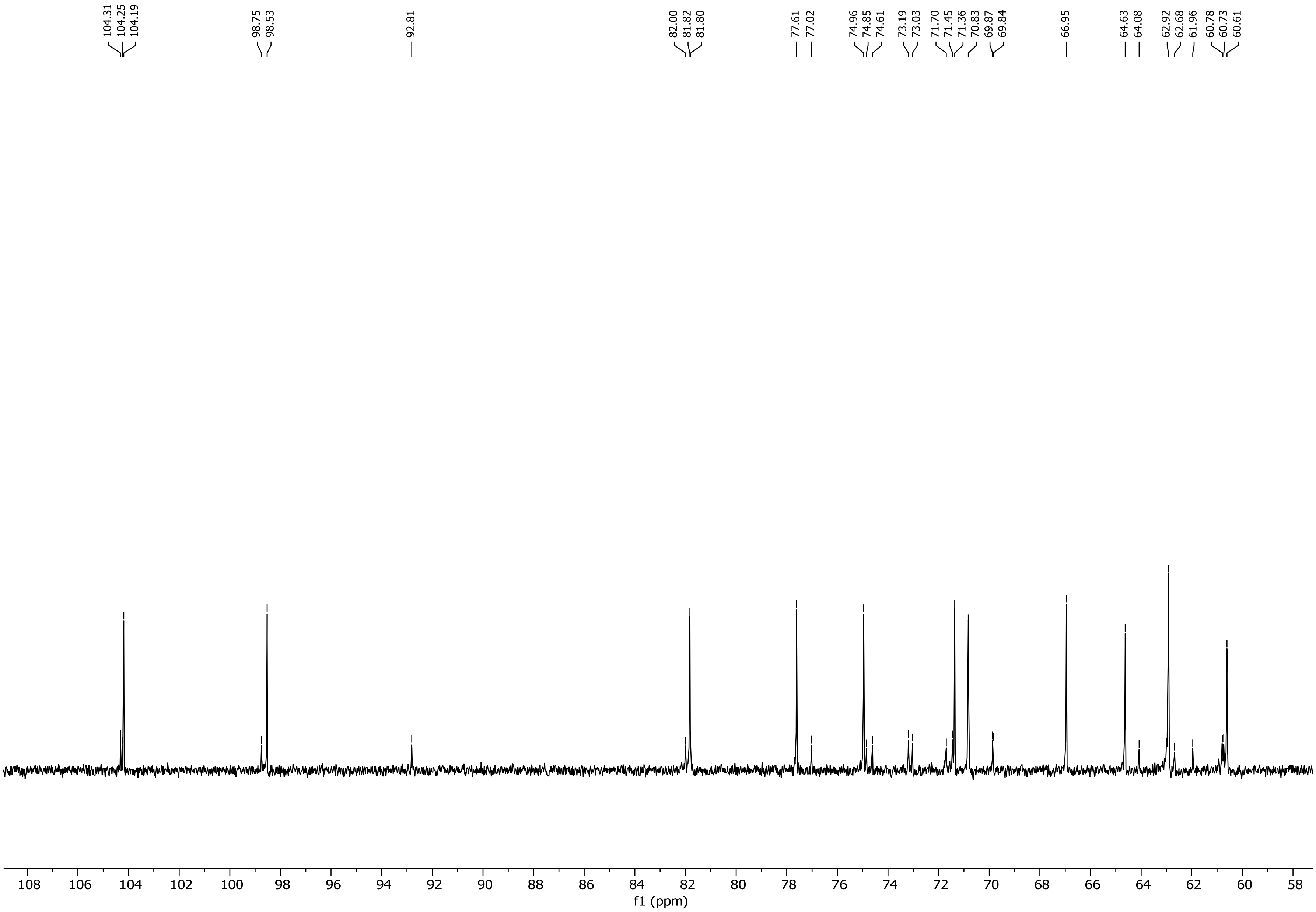

Figure S3. ^{13}C NMR (125 MHz, D_2O) of disaccharides **1α** and **1β**.

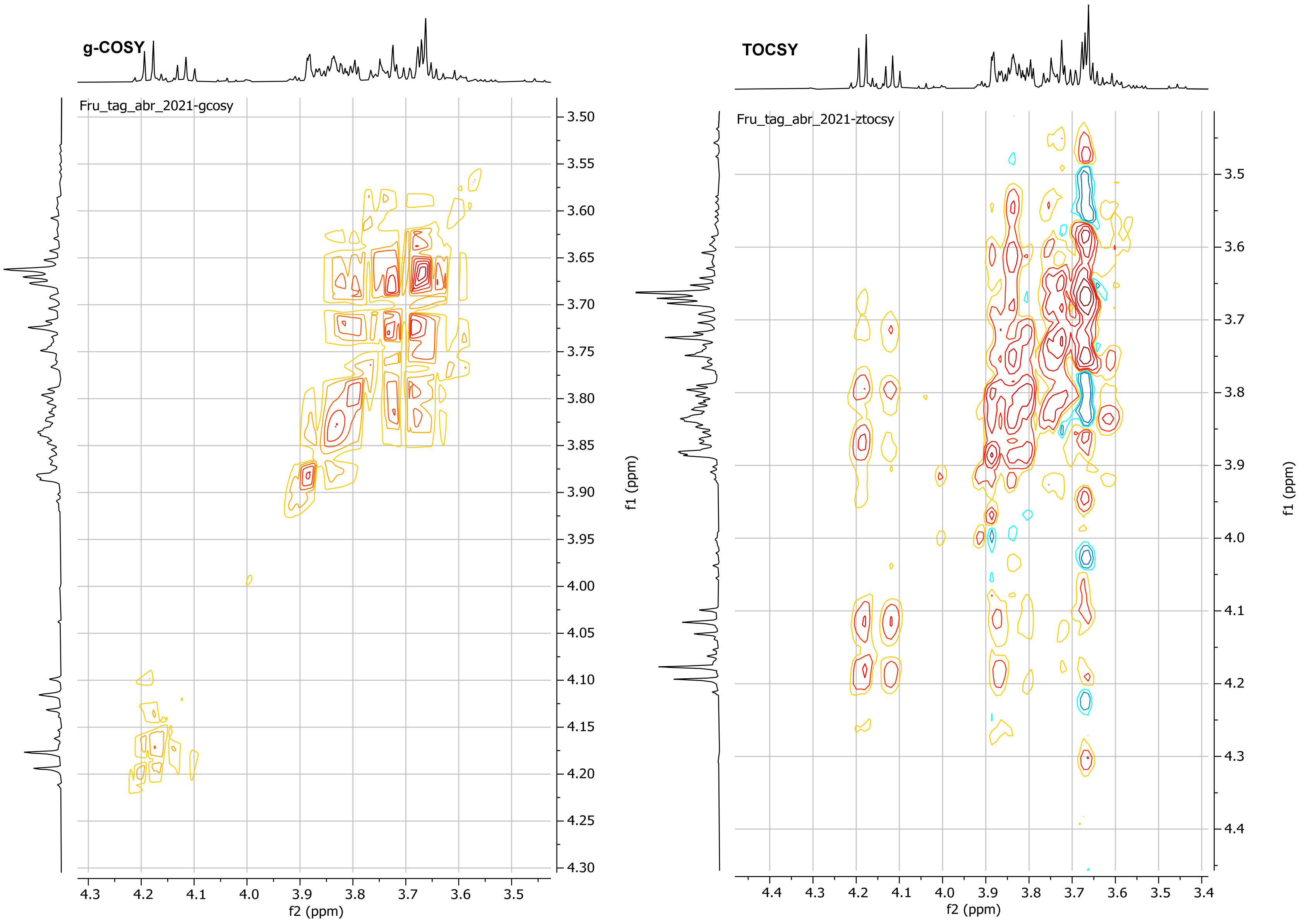

Figure S4. gCOSY and TOCSY (500 MHz, D_2O) of disaccharides **1α** and **1β**.

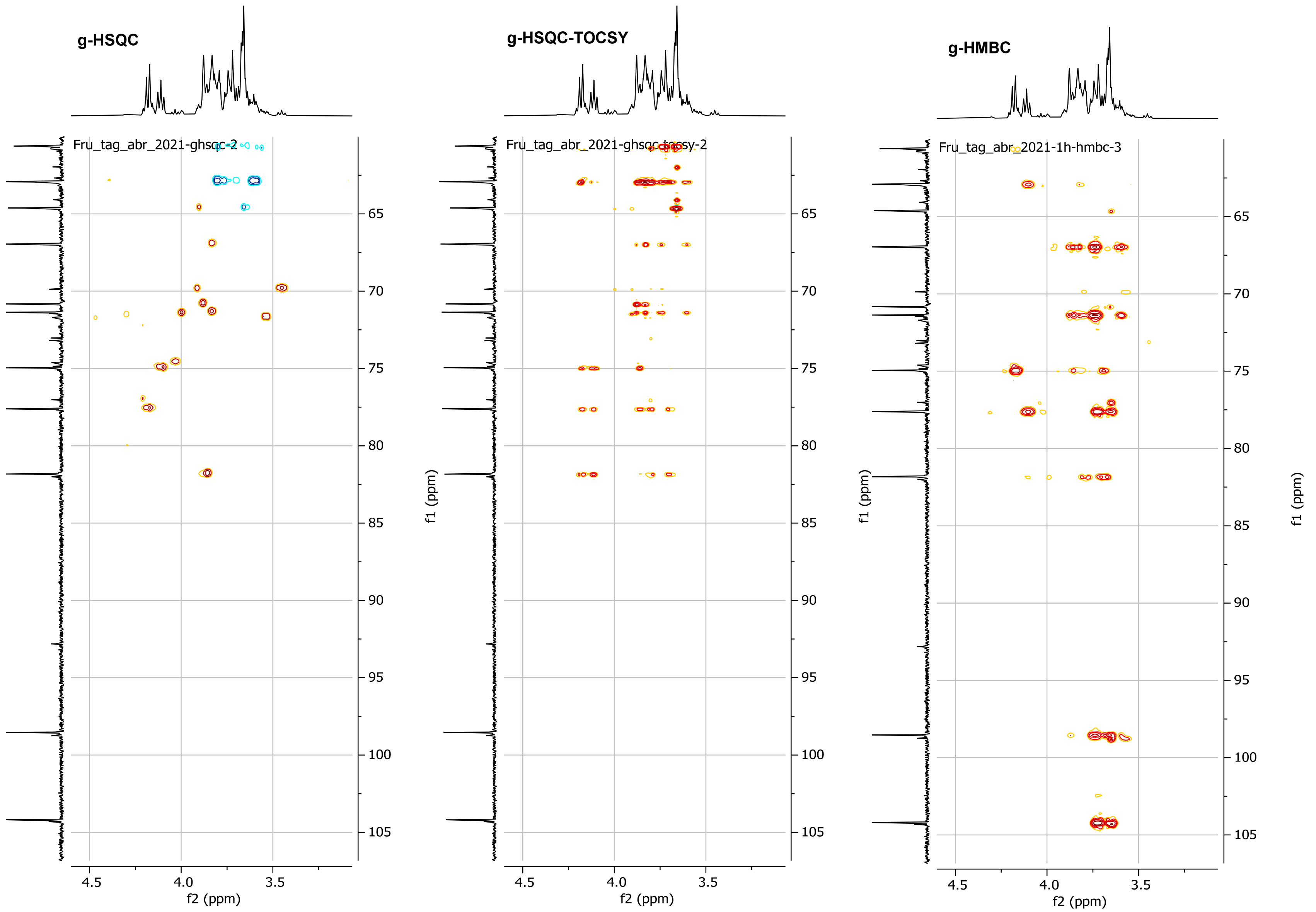

Figure S5. Multiplicity-edited gHSQC (methylene**:** blue cross peaks; methine**:** red cross peaks), gHSQC-TOCSY and gHMBC (500 MHz, D_2O) of disaccharides **1α** and **1β**.

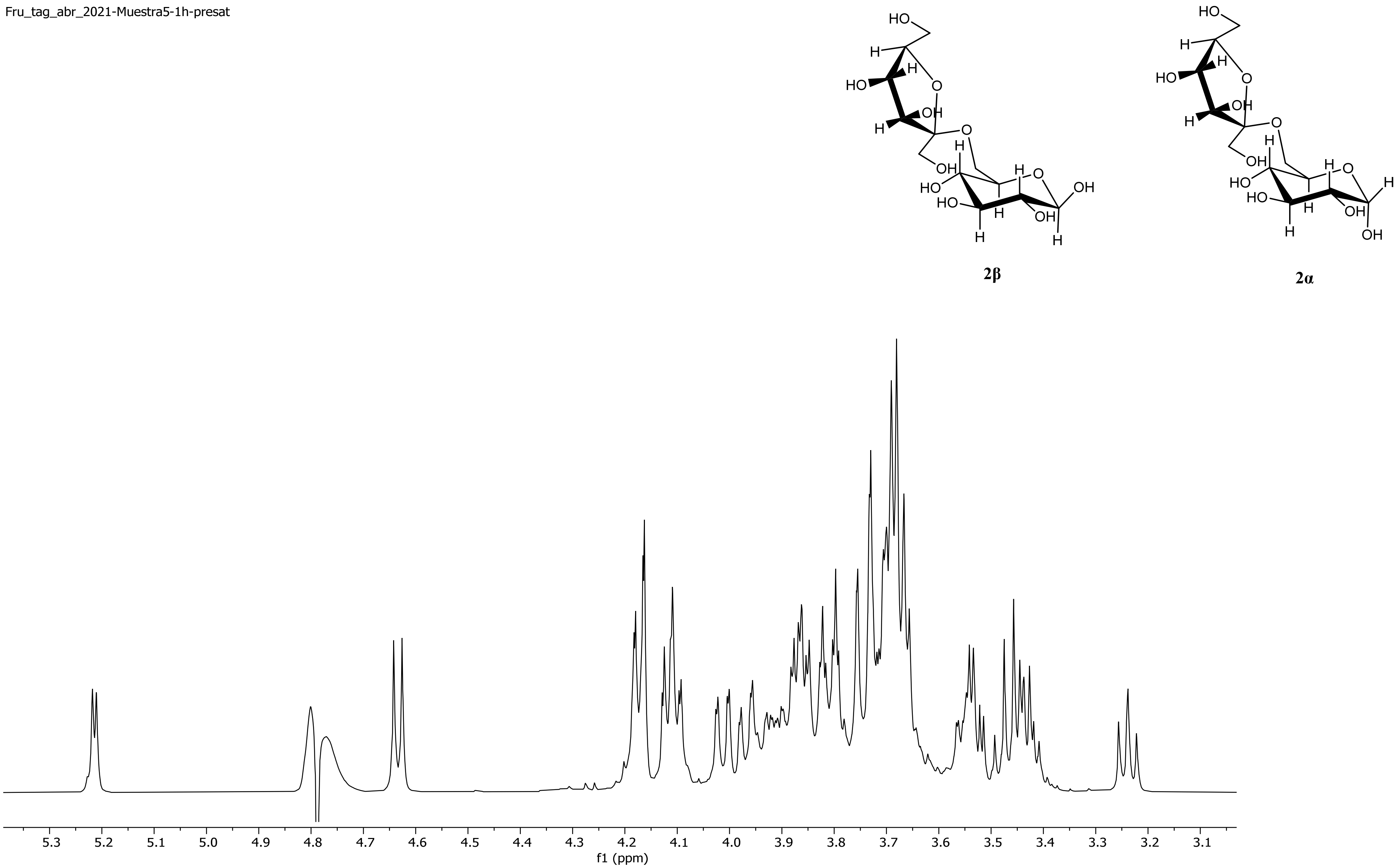

Figure S6. ^{1}H NMR (500 MHz, D_2O) of disaccharides **2α** and **2β**.

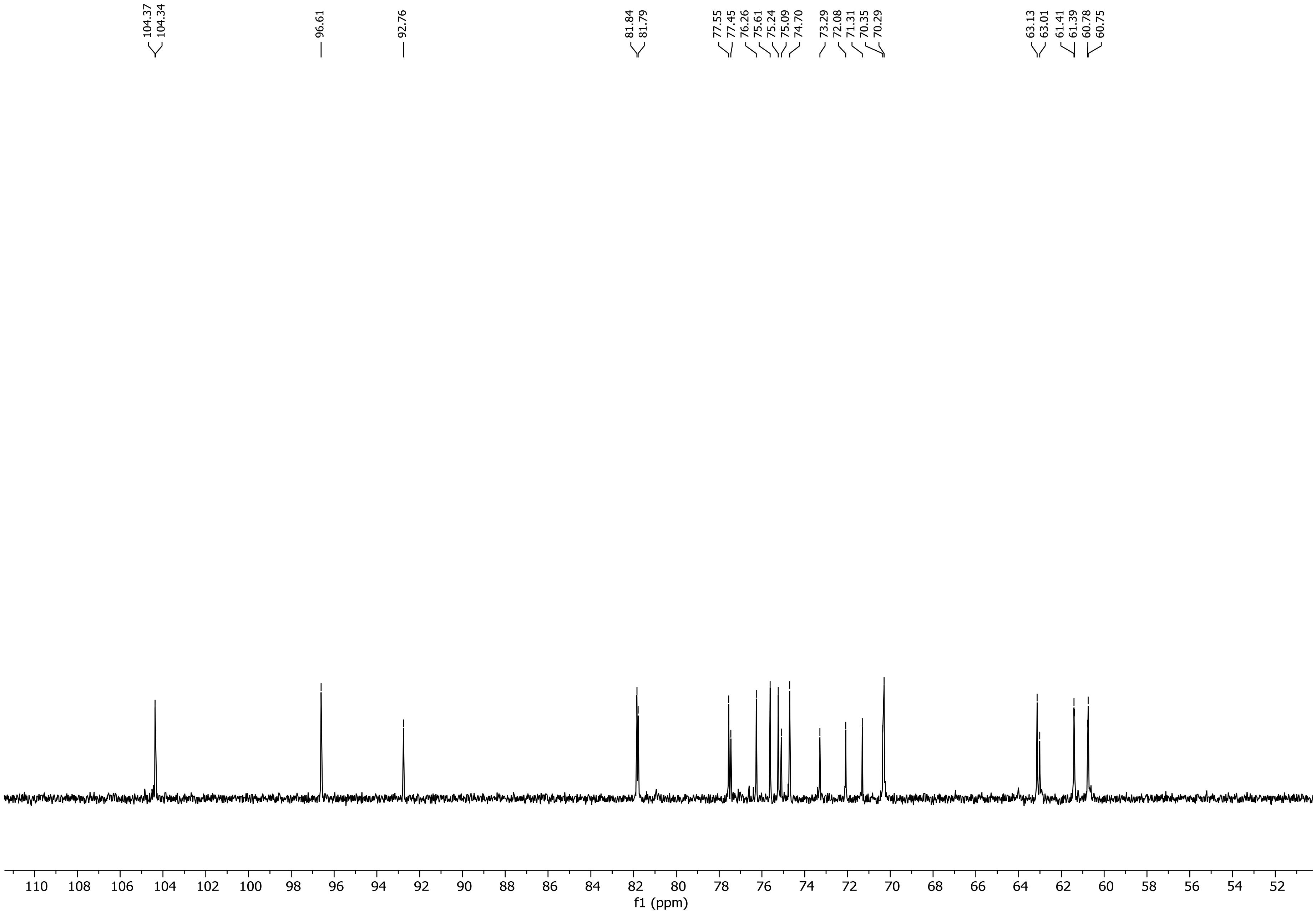

Figure S7. ^{13}C NMR (125 MHz, D_2O) of disaccharides **2α** and **2β**.

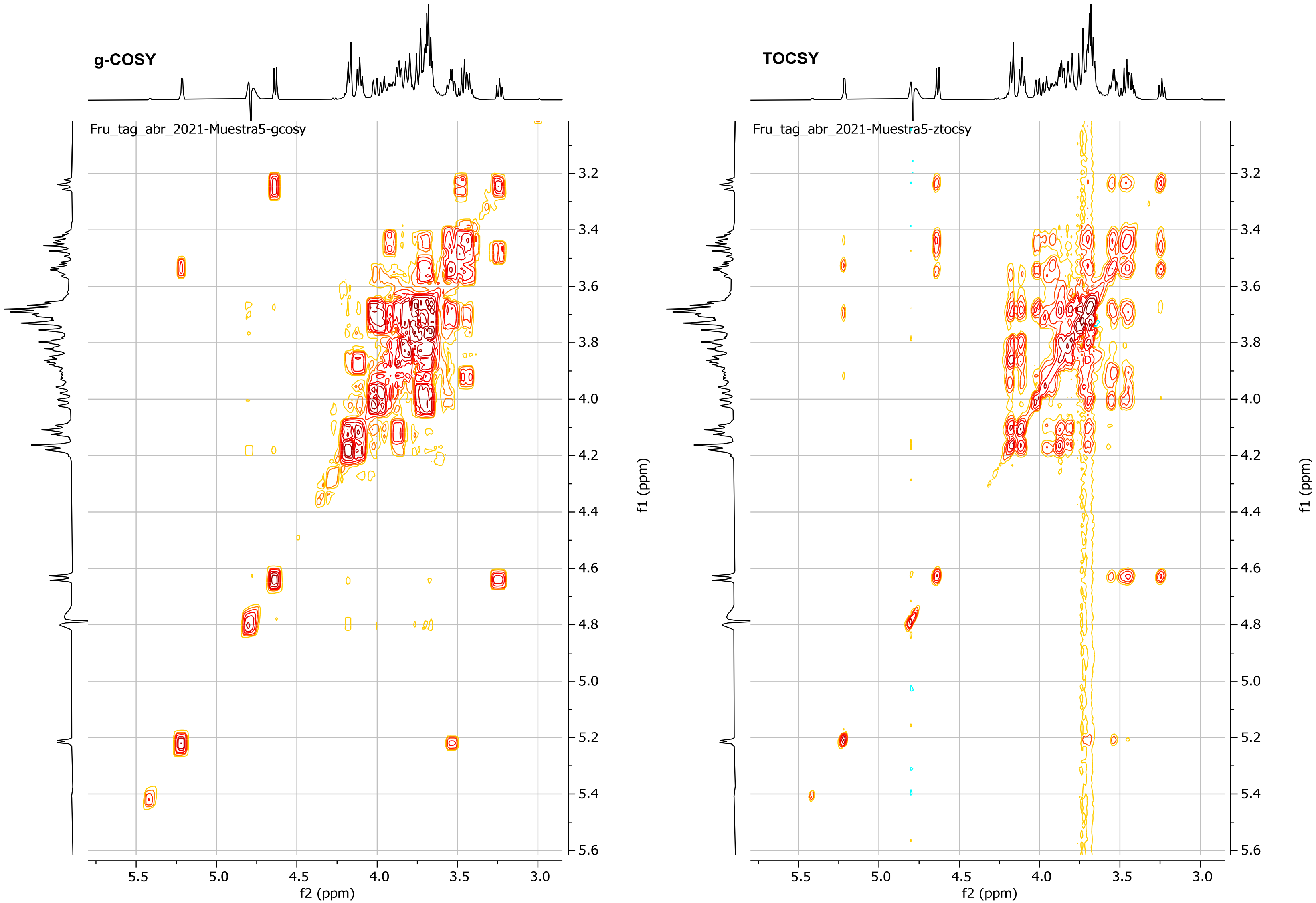

Figure S8. gCOSY and TOCSY (500 MHz, D_2O) of disaccharides **2α** and **2β**.

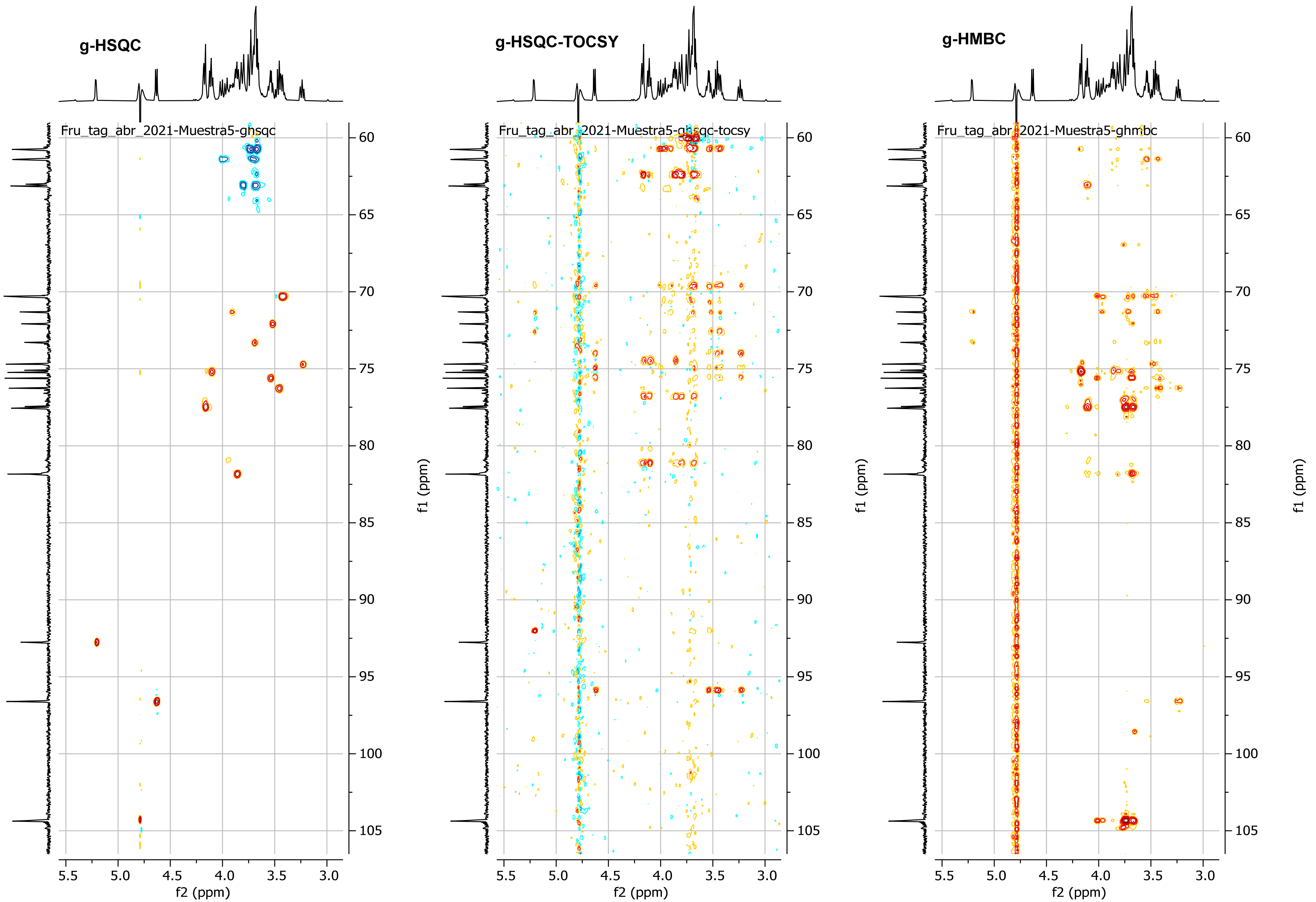

Figure S9. Multiplicity-edited gHSQC (methylene: blue cross peaks; methine: red cross peaks), gHSQC-TOCSY and gHMBC (500 MHz, D_2O) of disaccharides **2α** and **2β**.

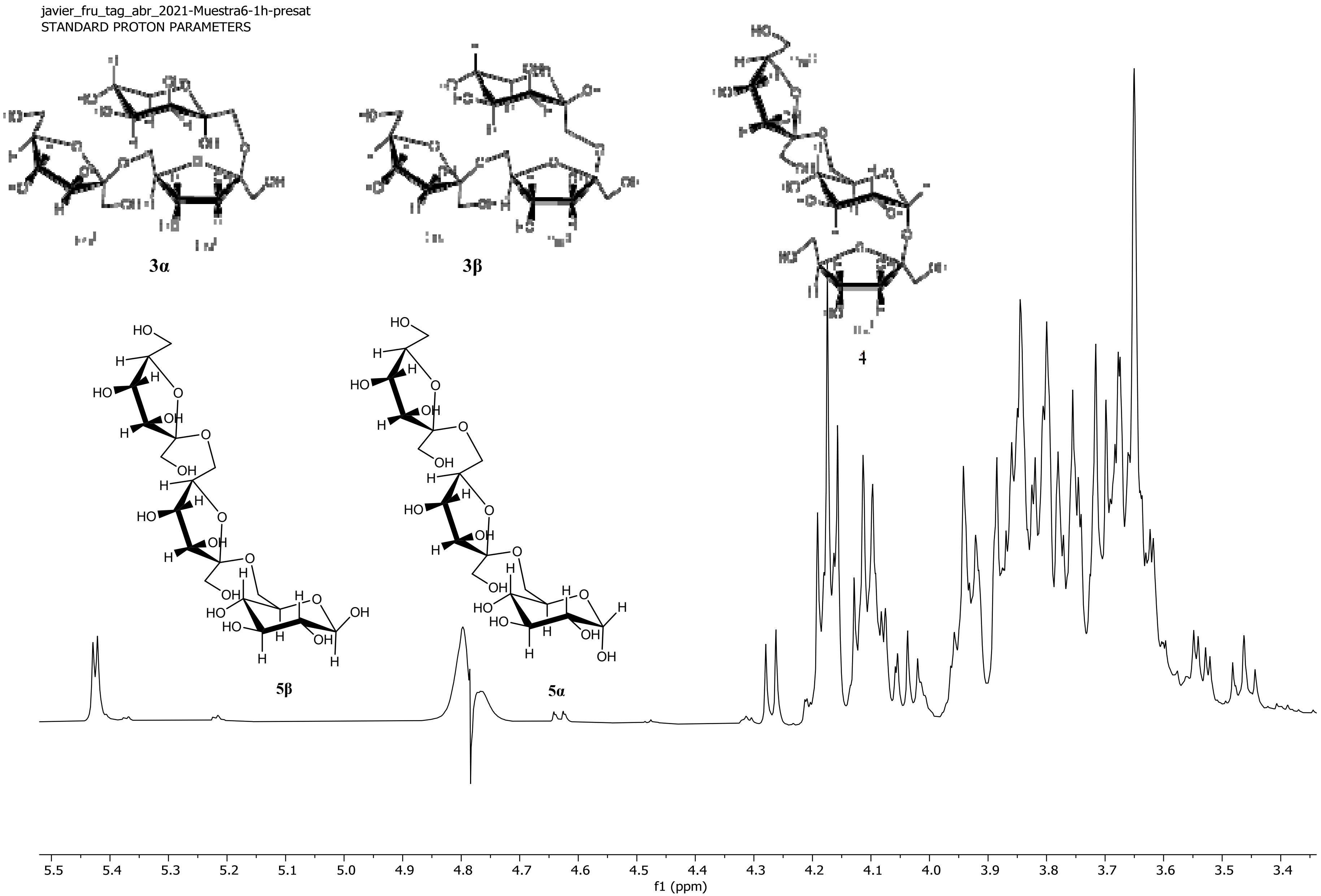

Figure S10. ^{1}H NMR (500 MHz, D_2O) for the mixture of trisaccharides **3α**, **3β**, **4**, **5α** and **5β**.

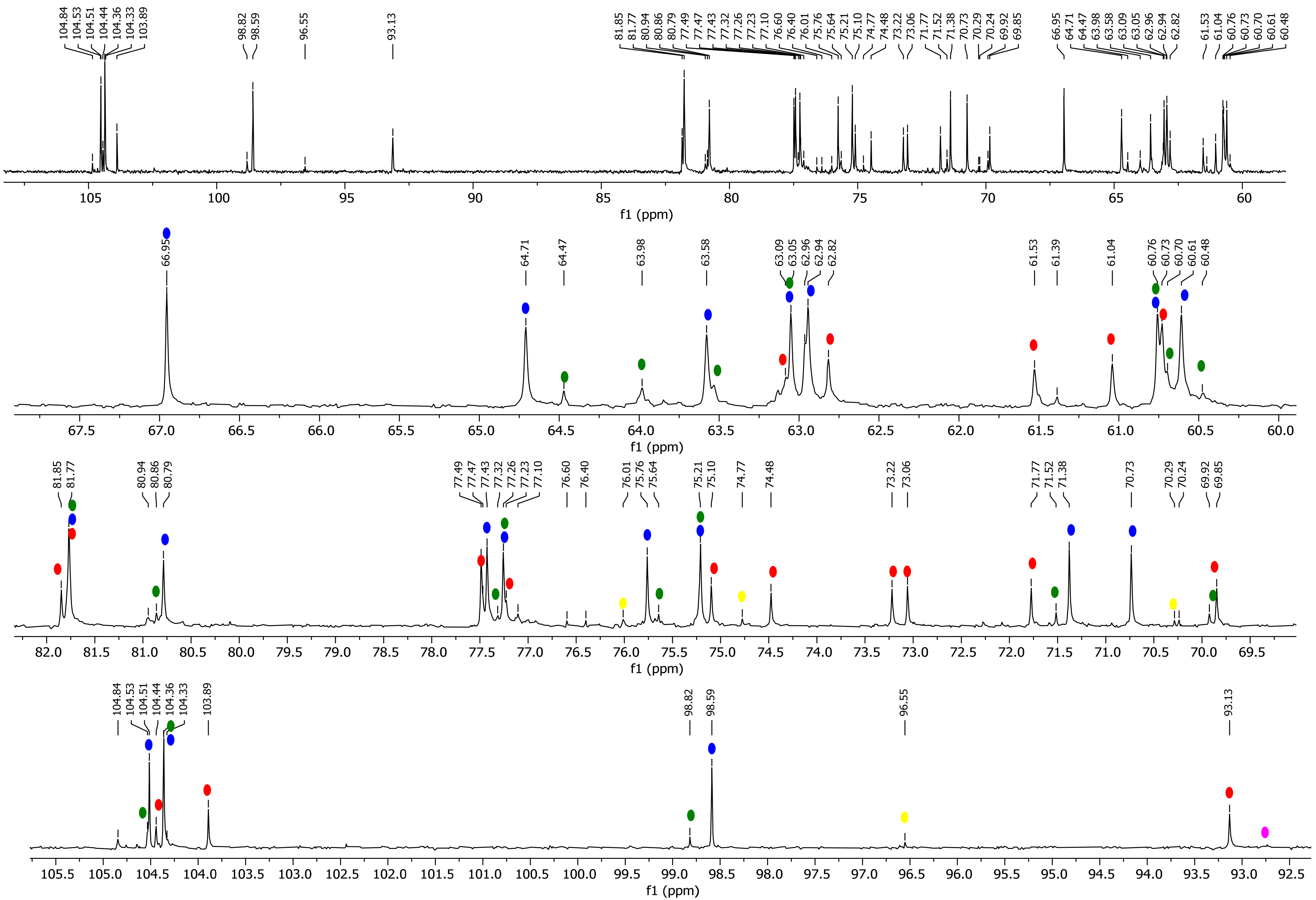

Figure S11. ^{13}C NMR (125 MHz, D_2O, full spectrum and enlargements with assignations) for the mixture of trisaccharides **3α** (blue), **3β** (green), **4** (red), **5α** (purple) and **5β** (yellow).

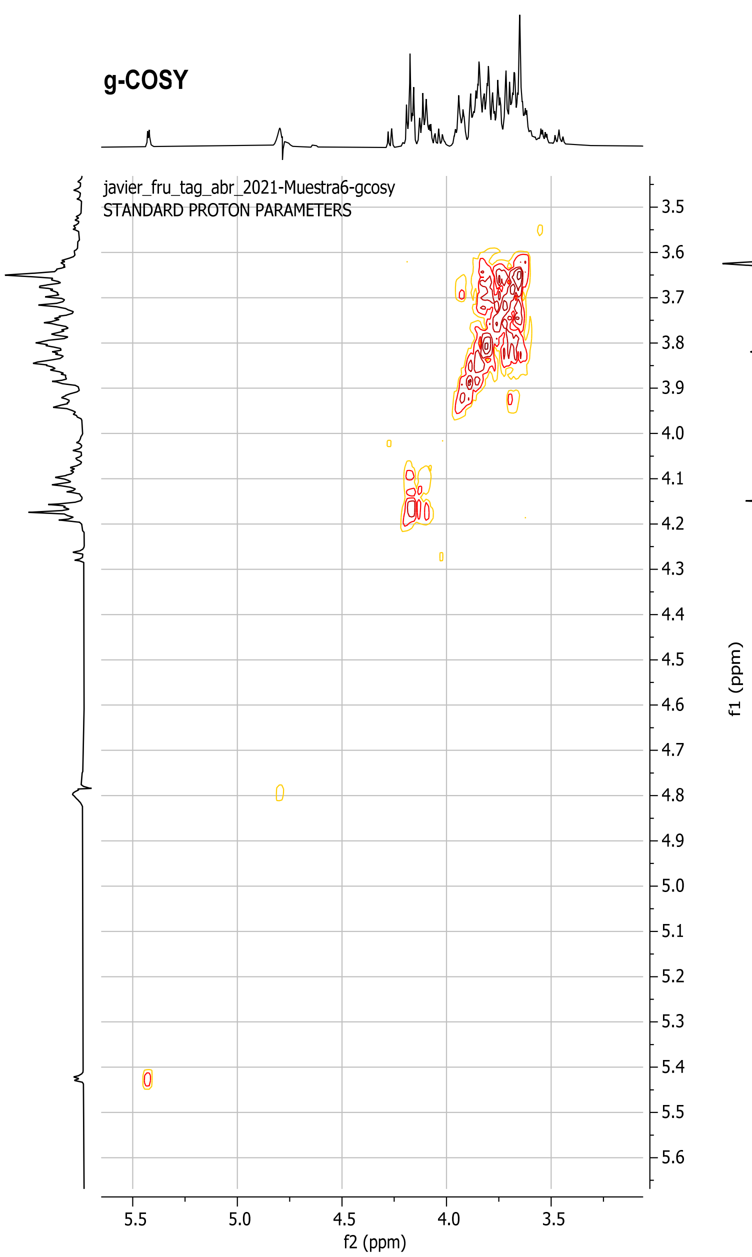

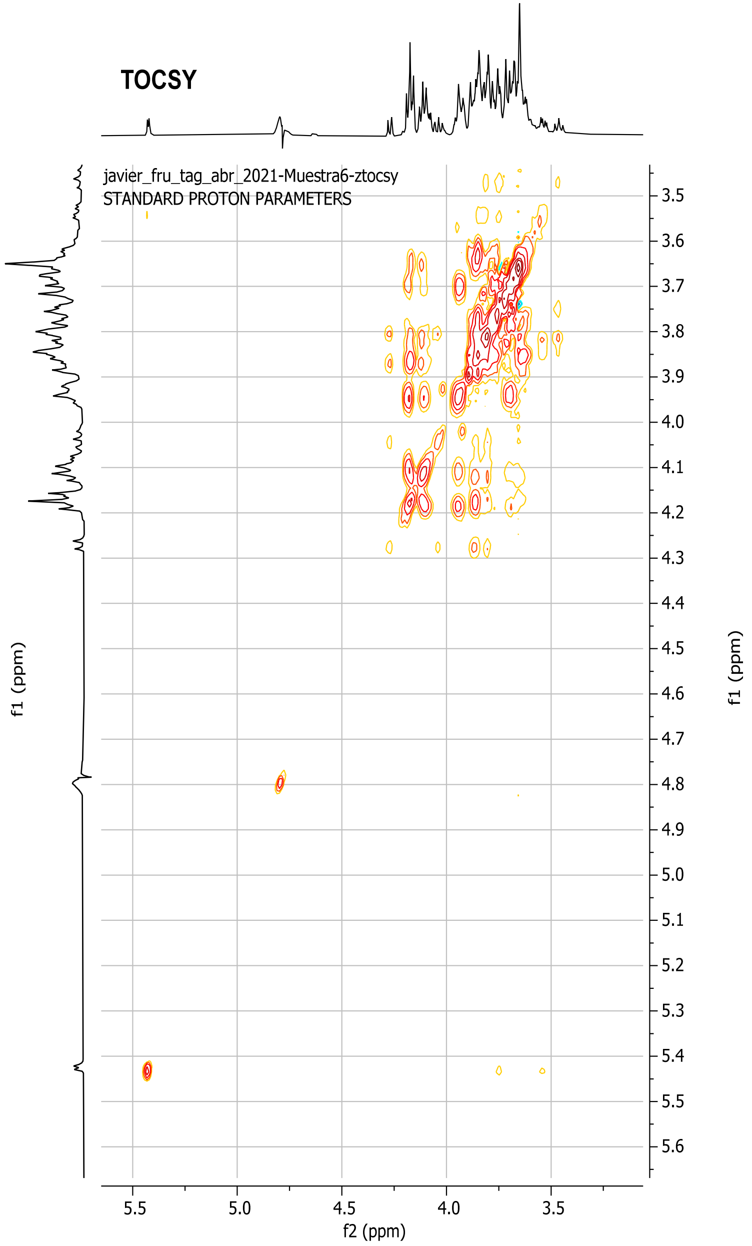

Figure S12. gCOSY and TOCSY (500 MHz, D_2O) for the mixture of trisaccharides **3α**, **3β**, **4**, **5α** and **5β**.

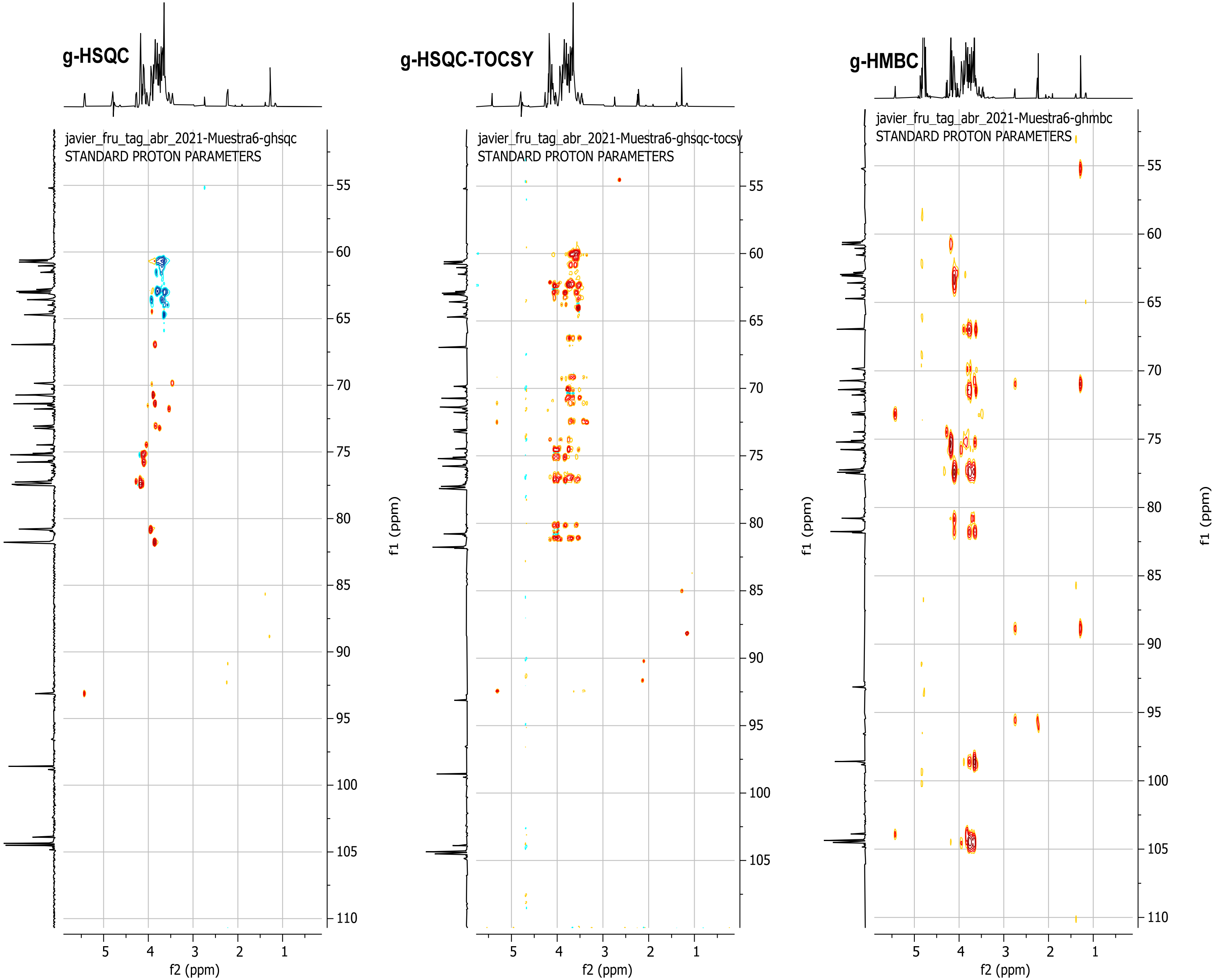

Figure S13. Multiplicity-edited gHSQC (methylene**:** blue cross peaks; methine**:** red cross peaks), gHSQC-TOCSY and gHMBC (500 MHz, D_2O) for the mixture of trisaccharides **3α**, **3β**, **4**, **5α** and **5β**.

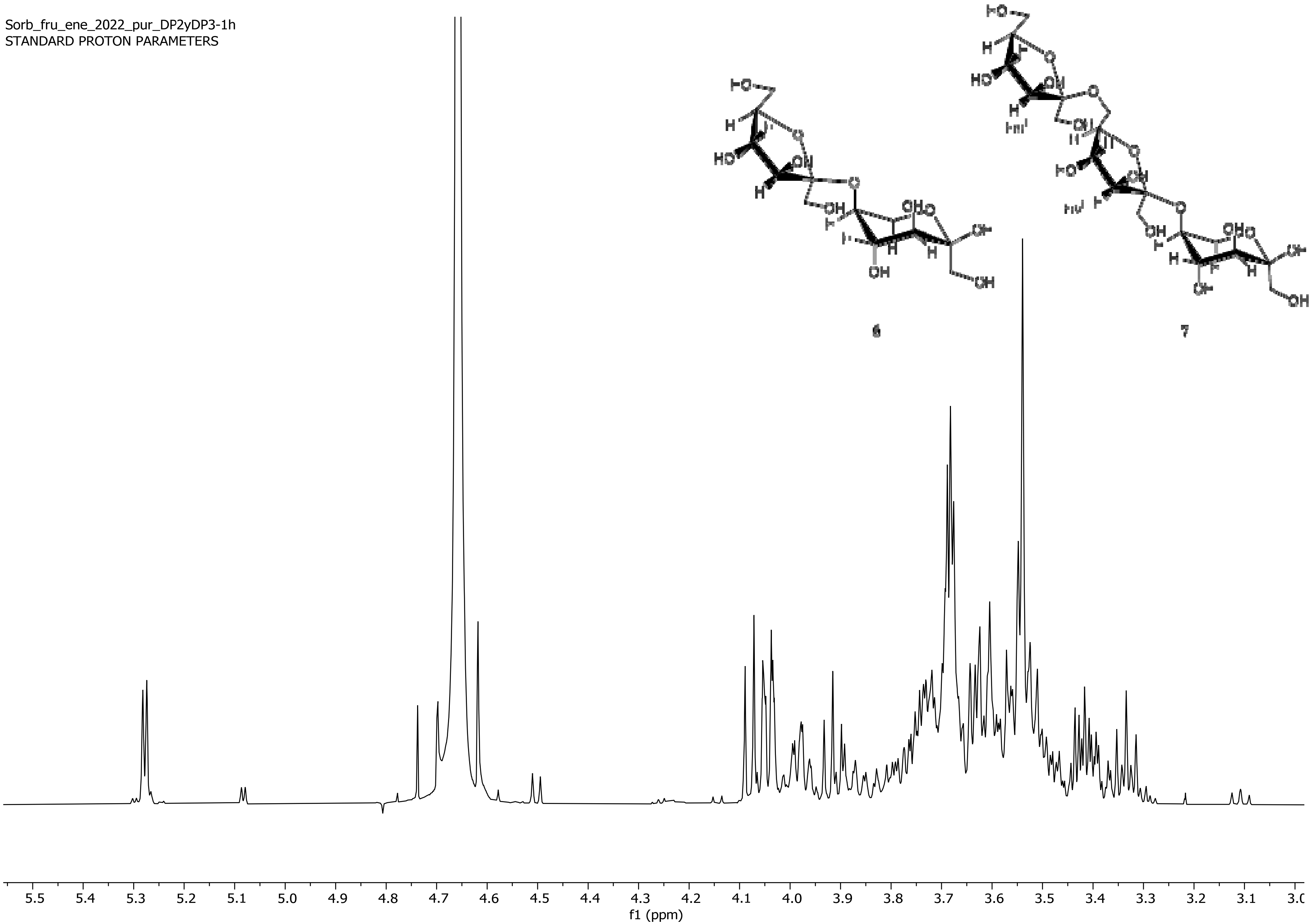

Figure S14. ^{1}H NMR (500 MHz, D_2O) for the mixture of disaccharide **6** and trisaccharide **7**.

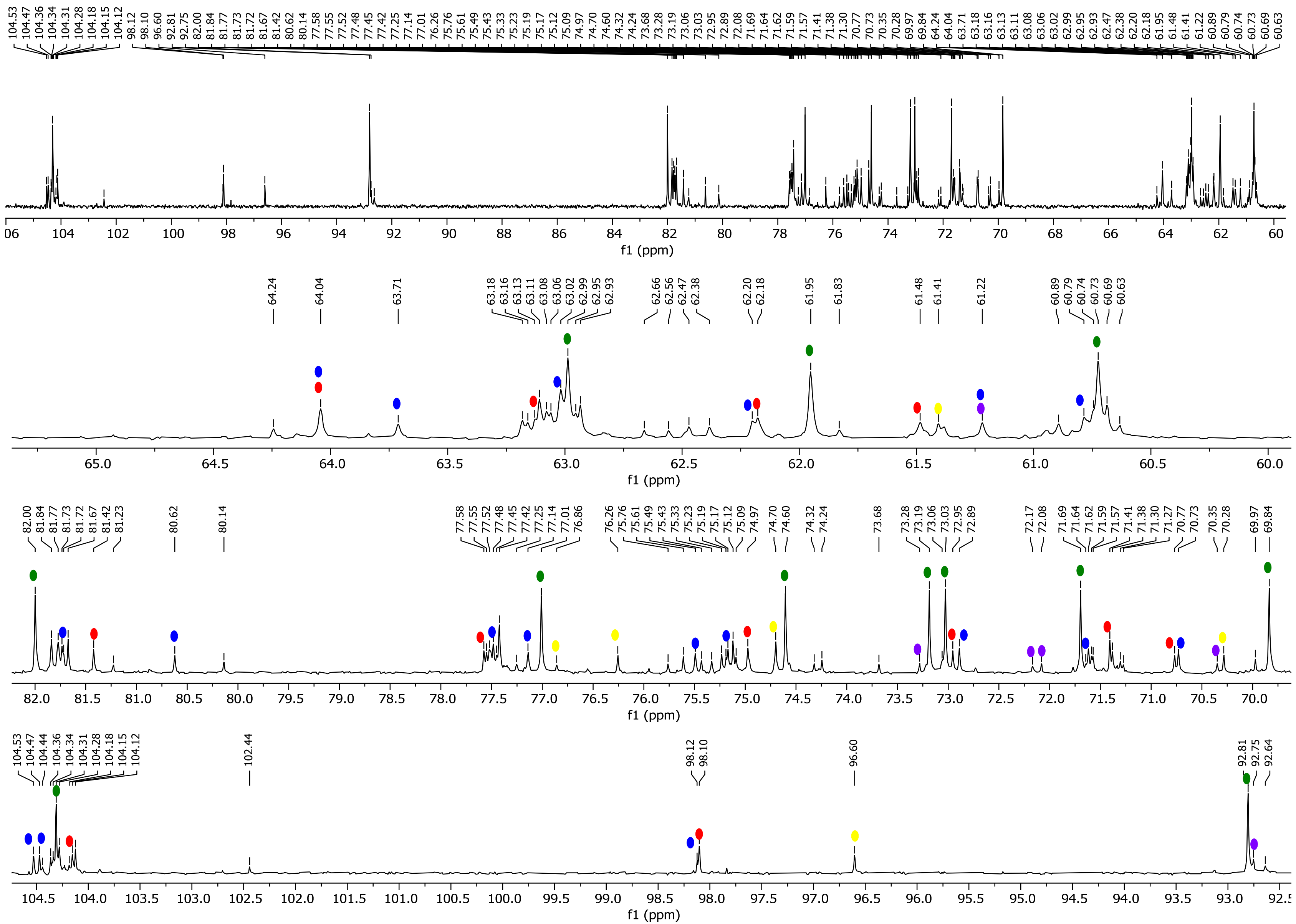

Figure S15. ^{13}C NMR (125 MHz, D_2O, full spectrum and enlargements with assignations) for the mixture of disaccharide **6** (red) and trisaccharide **7** (blue). [Sucrose (green) and α- and β-glucopyranose (purple and yellow, respectively) have been also identified].

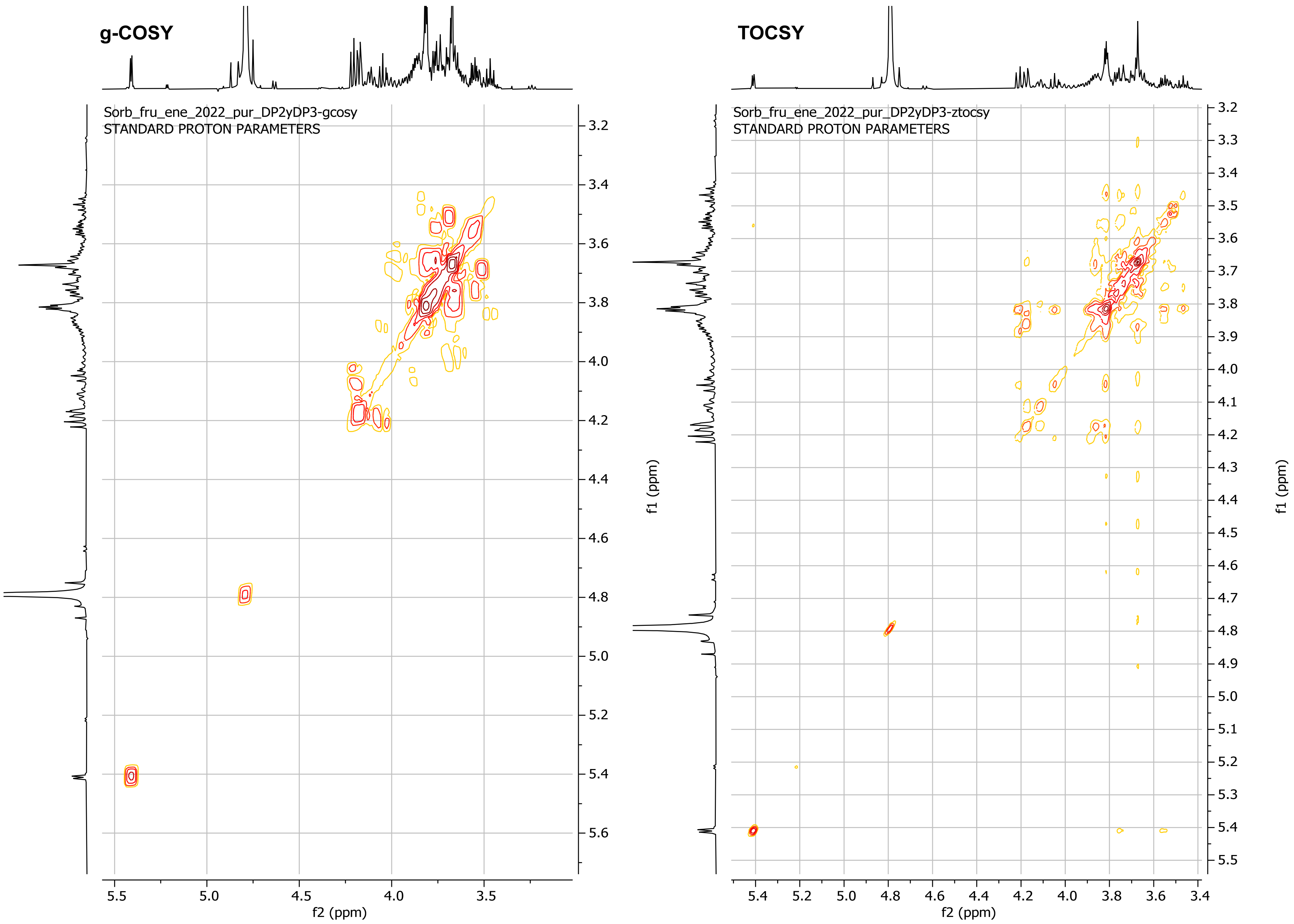

Figure S16. gCOSY and TOCSY (500 MHz, D_2O) for the mixture of disaccharide **6** and trisaccharide **7**.

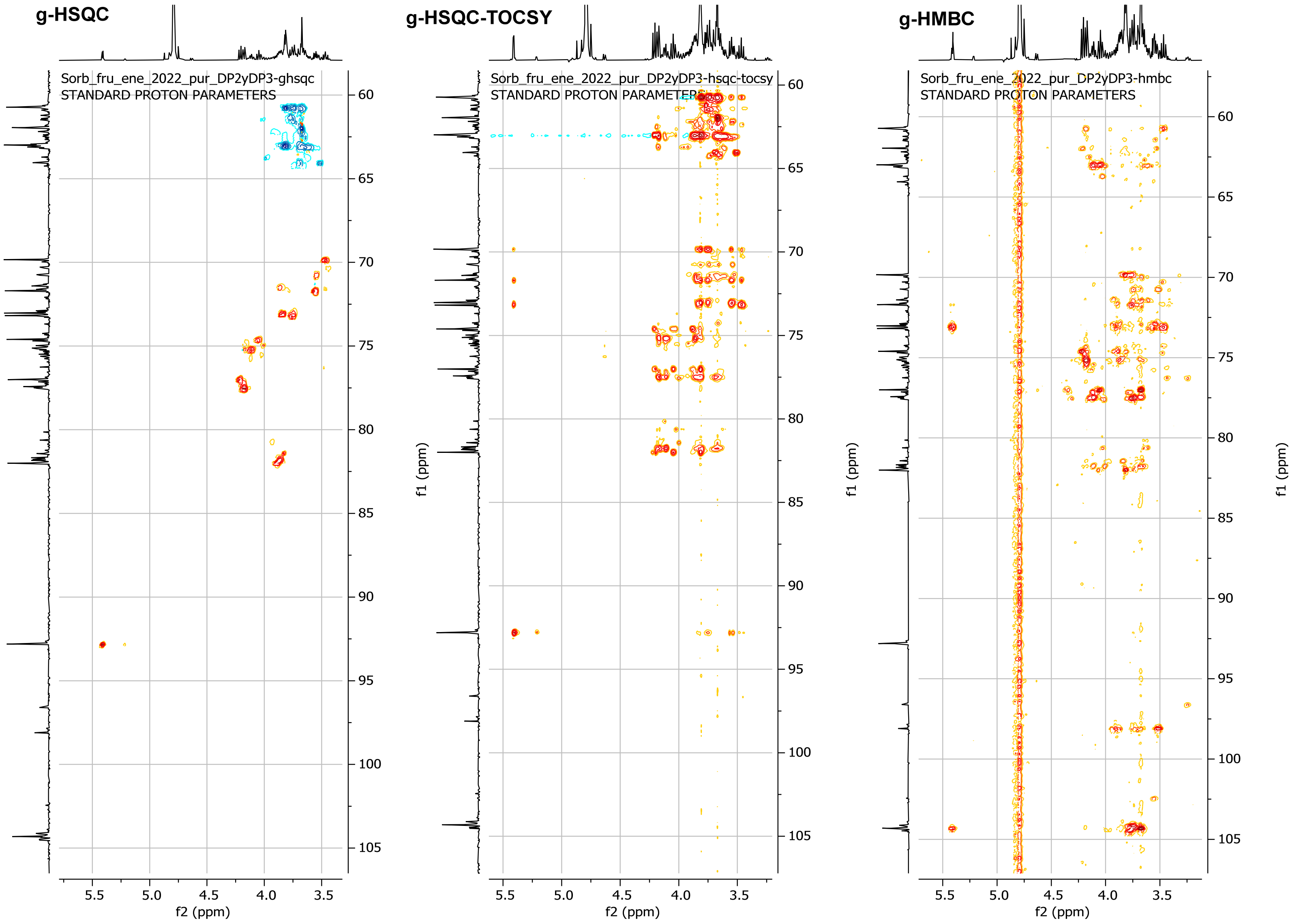

Figure S17. Multiplicity-edited gHSQC (methylene**:** blue cross peaks; methine**:** red cross peaks), gHSQC-TOCSY and gHMBC (500 MHz, D_2O) for the mixture of disaccharide **6** and trisaccharide **7**.

Sorb_fru_ene_2022_pur_DP3-1h-presat
STANDARD PROTON PARAMETERS

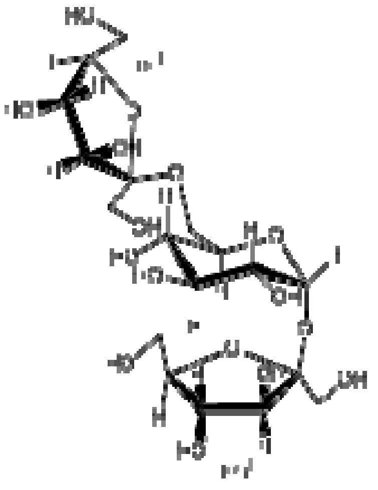

4

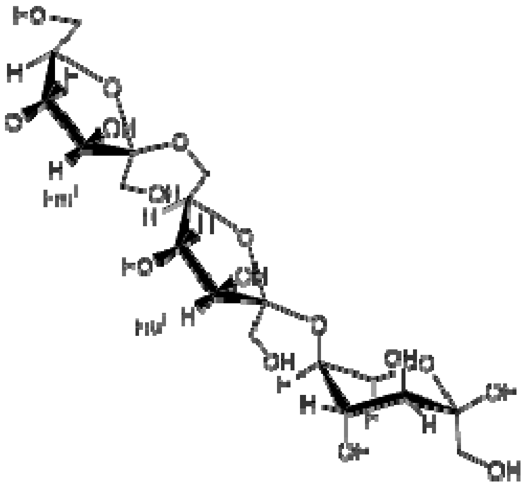

7

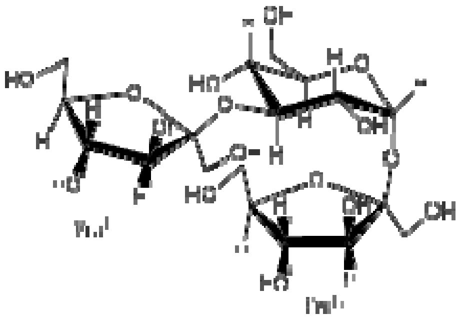

8

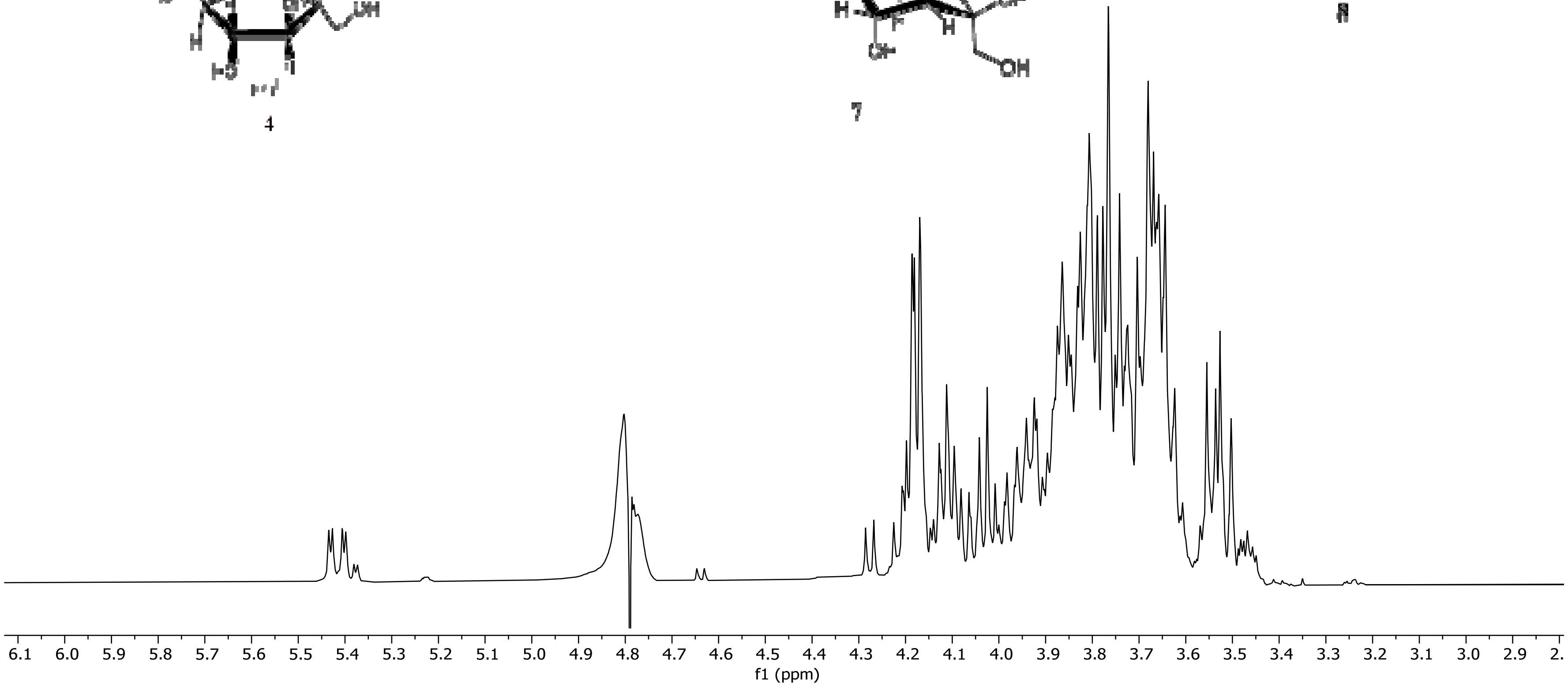

Figure S18. ^{1}H NMR (500 MHz, D_2O) for the mixture of trisaccharides **4** ,**7** and **8**.

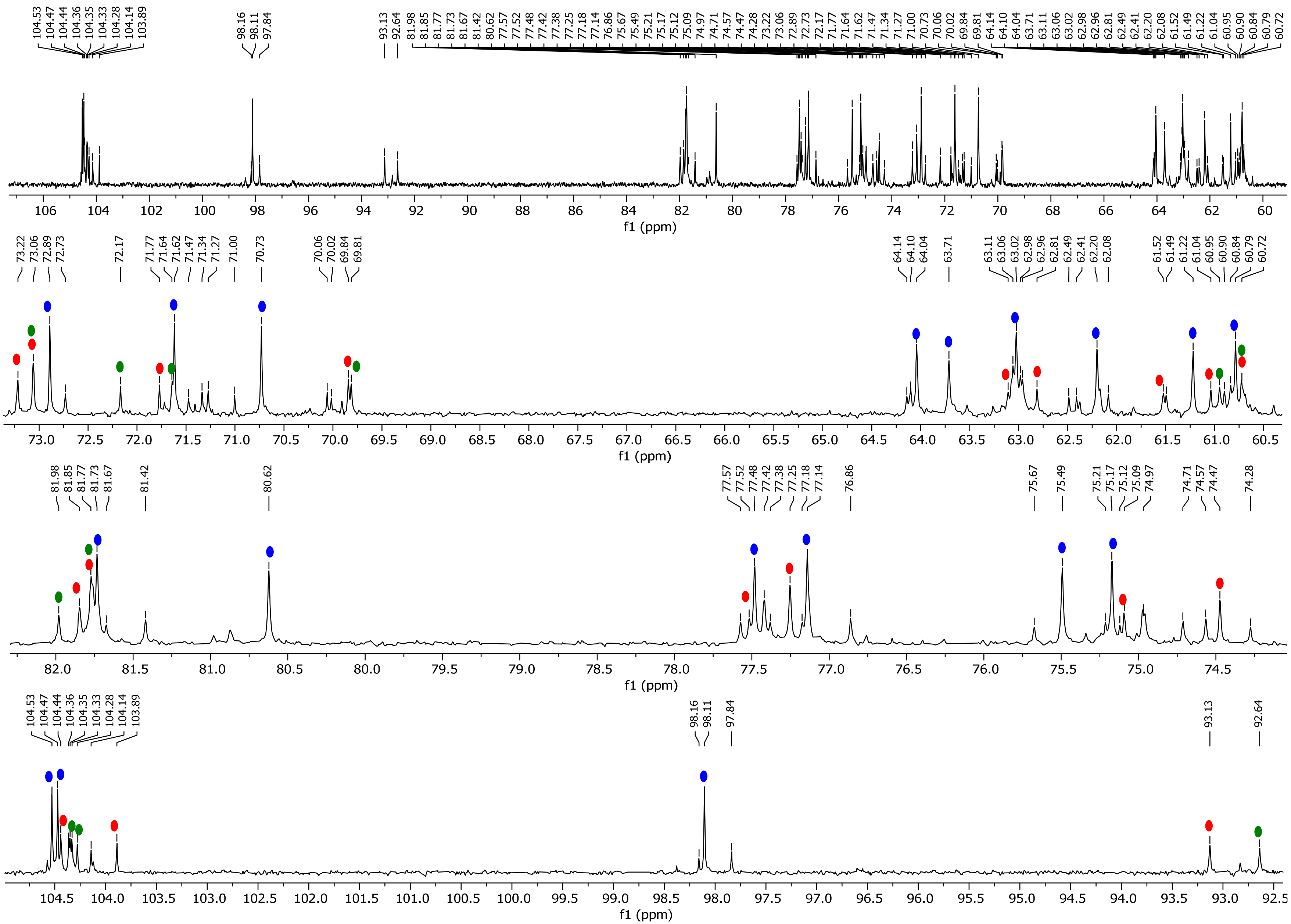

Figure S19.^{13}C NMR (125 MHz, D_2O, full spectrum and enlargements with assignations) for the mixture of trisaccharides **4** (red), **7** (blue) and **8** (green).

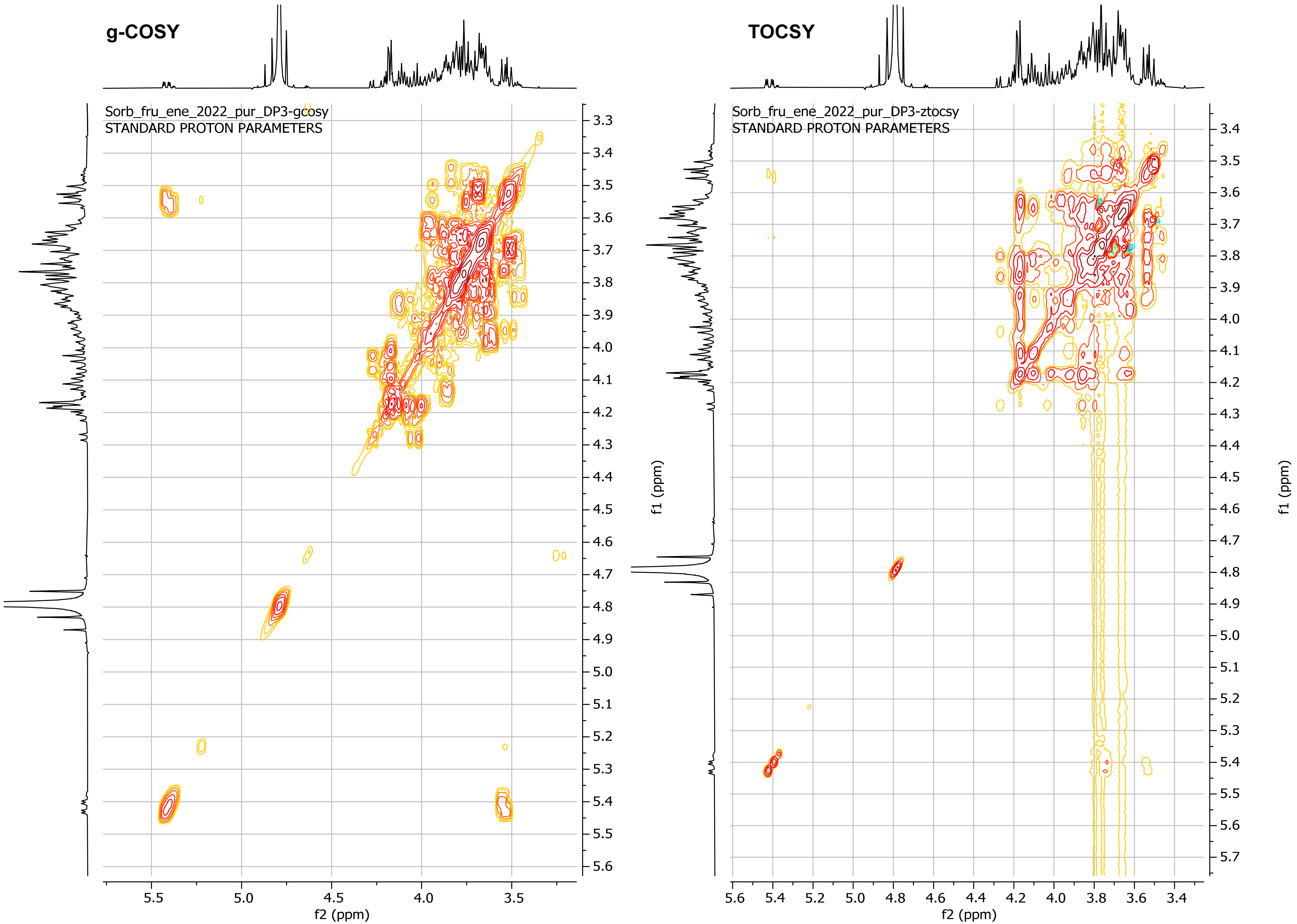

Figure S20. gCOSY and TOCSY (500 MHz, D_2O) for the mixture of trisaccharides **4** ,**7** and **8**.

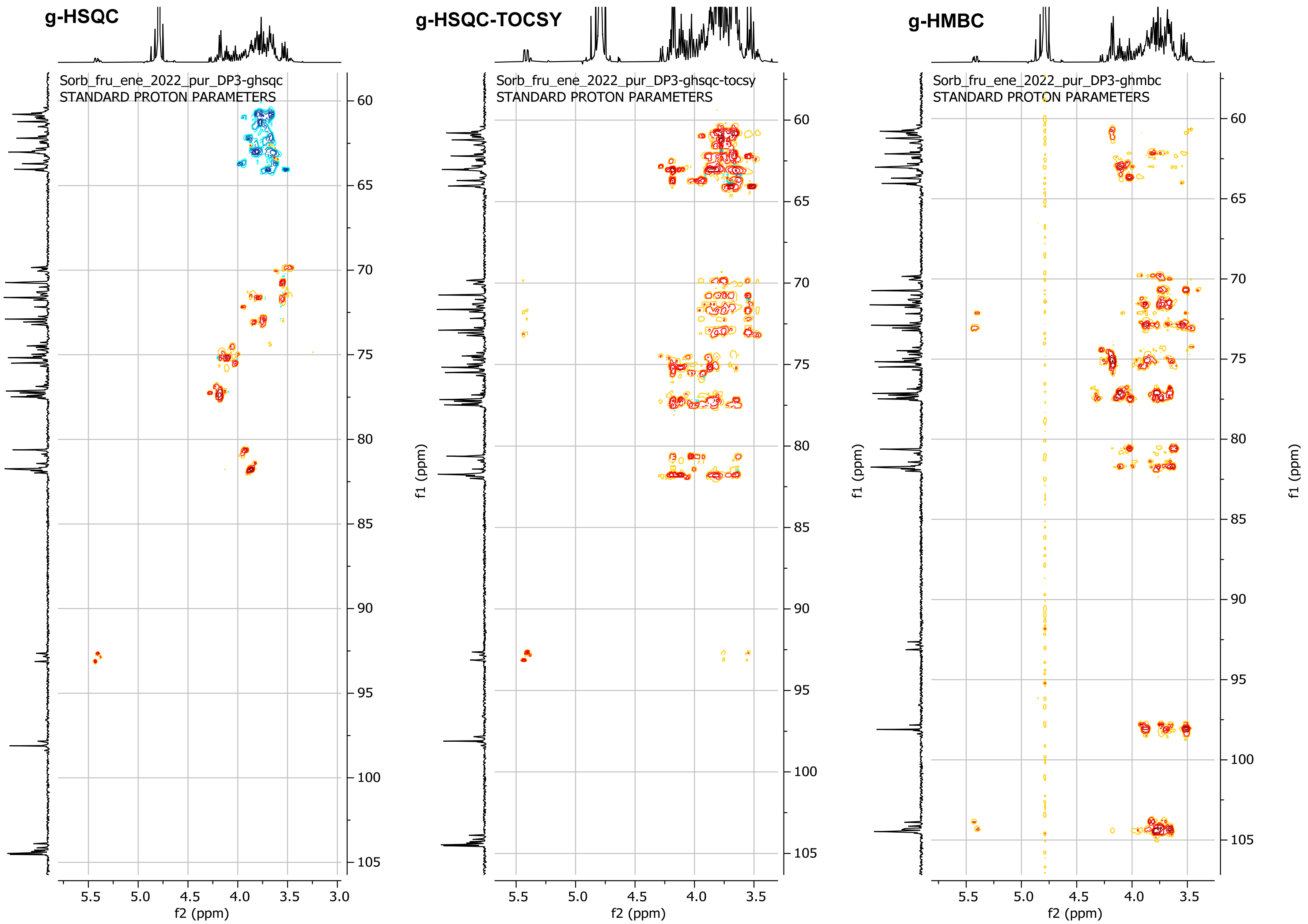

Figure S21. Multiplicity-edited gHSQC (methylene**:** blue cross peaks; methine**:** red cross peaks), gHSQC-TOCSY and gHMBC (500 MHz, D_2O) for the mixture of trisaccharides **4** ,**7** and **8**.

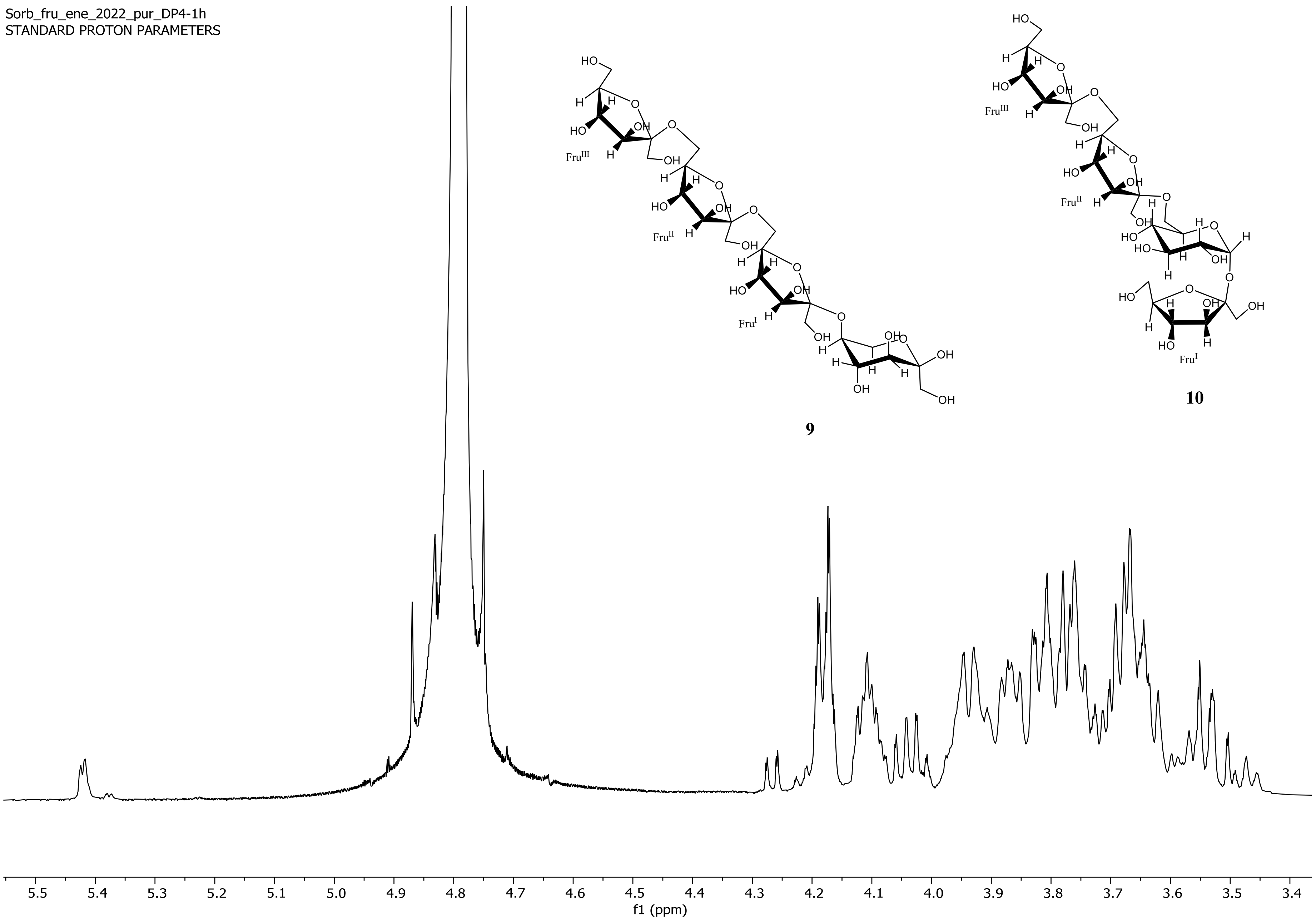

Figure S22. ^{1}H NMR (500 MHz, D_2O) for the mixture of tetrasaccharides **9** and **10**.

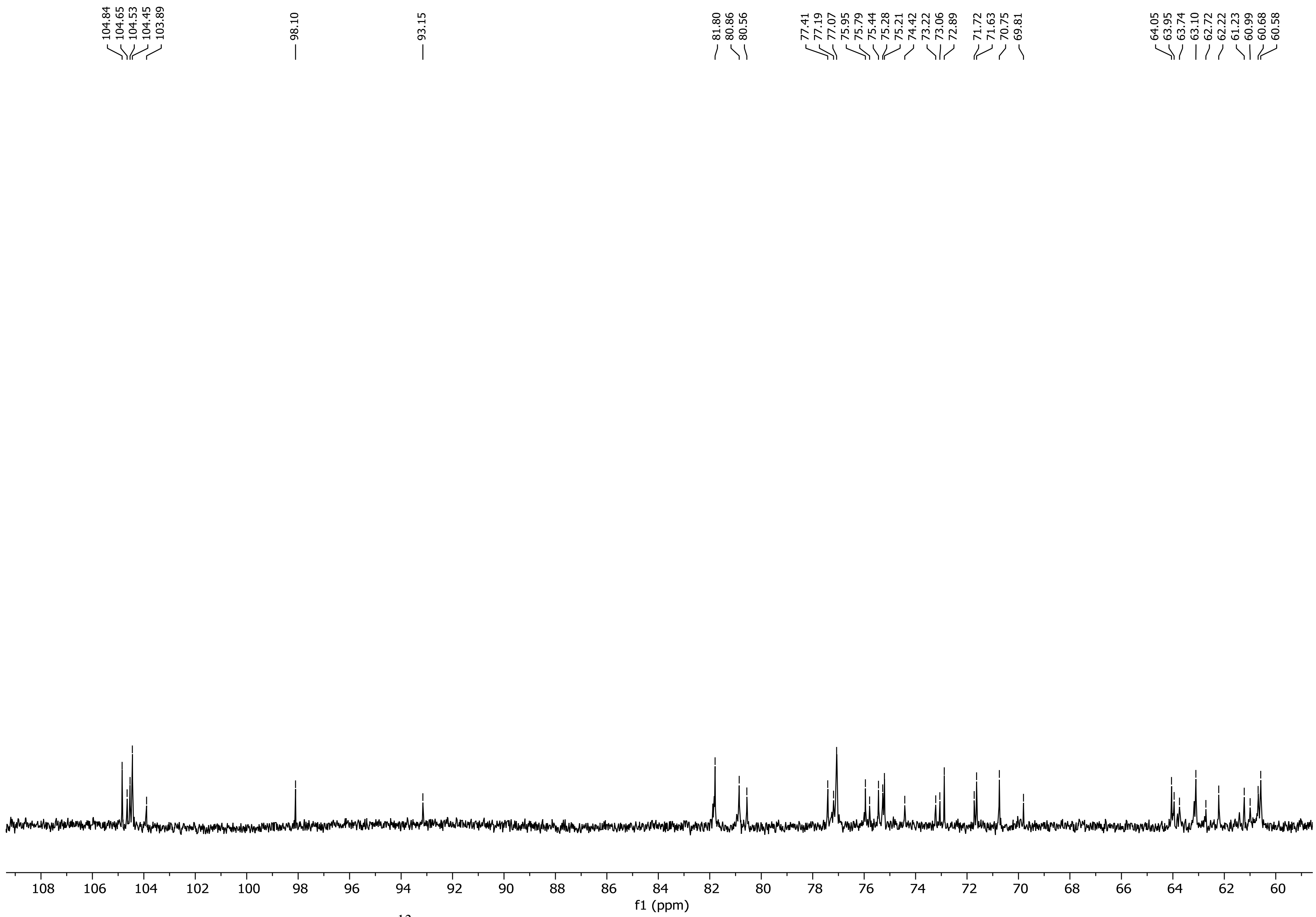

Figure S23.^{13}C NMR (125 MHz, D_2O) for the mixture of tetrasaccharides **9** and **10**.

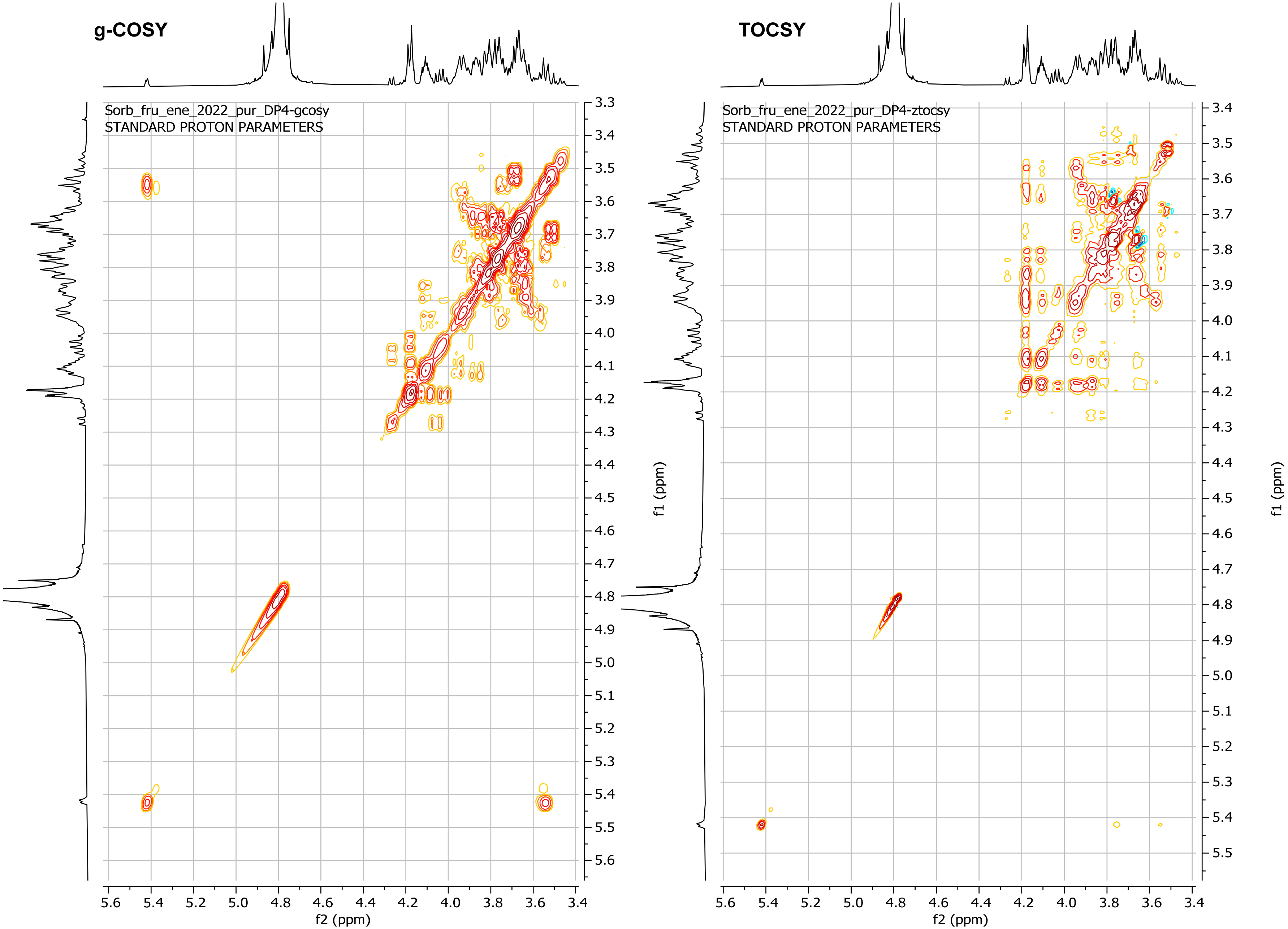

Figure S24. gCOSY and TOCSY (500 MHz, D_2O) for the mixture of tetrasaccharides **9** and **10**.

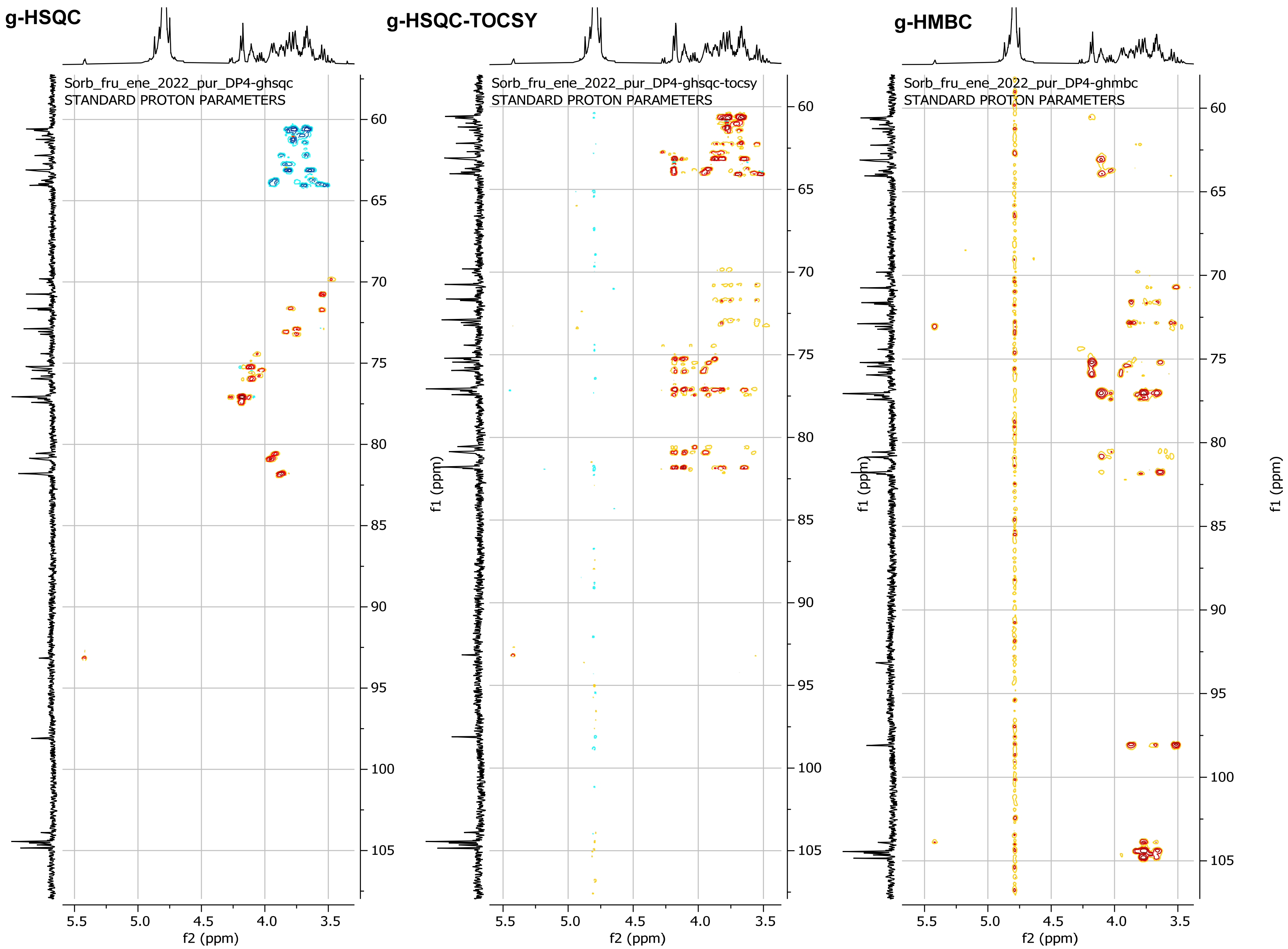

Figure S25. Multiplicity-edited gHSQC (methylene**:** blue cross peaks; methine**:** red cross peaks), gHSQC-TOCSY and gHMBC (500 MHz, D_2O) for the mixture of tetrasaccharides **9** and **10**.

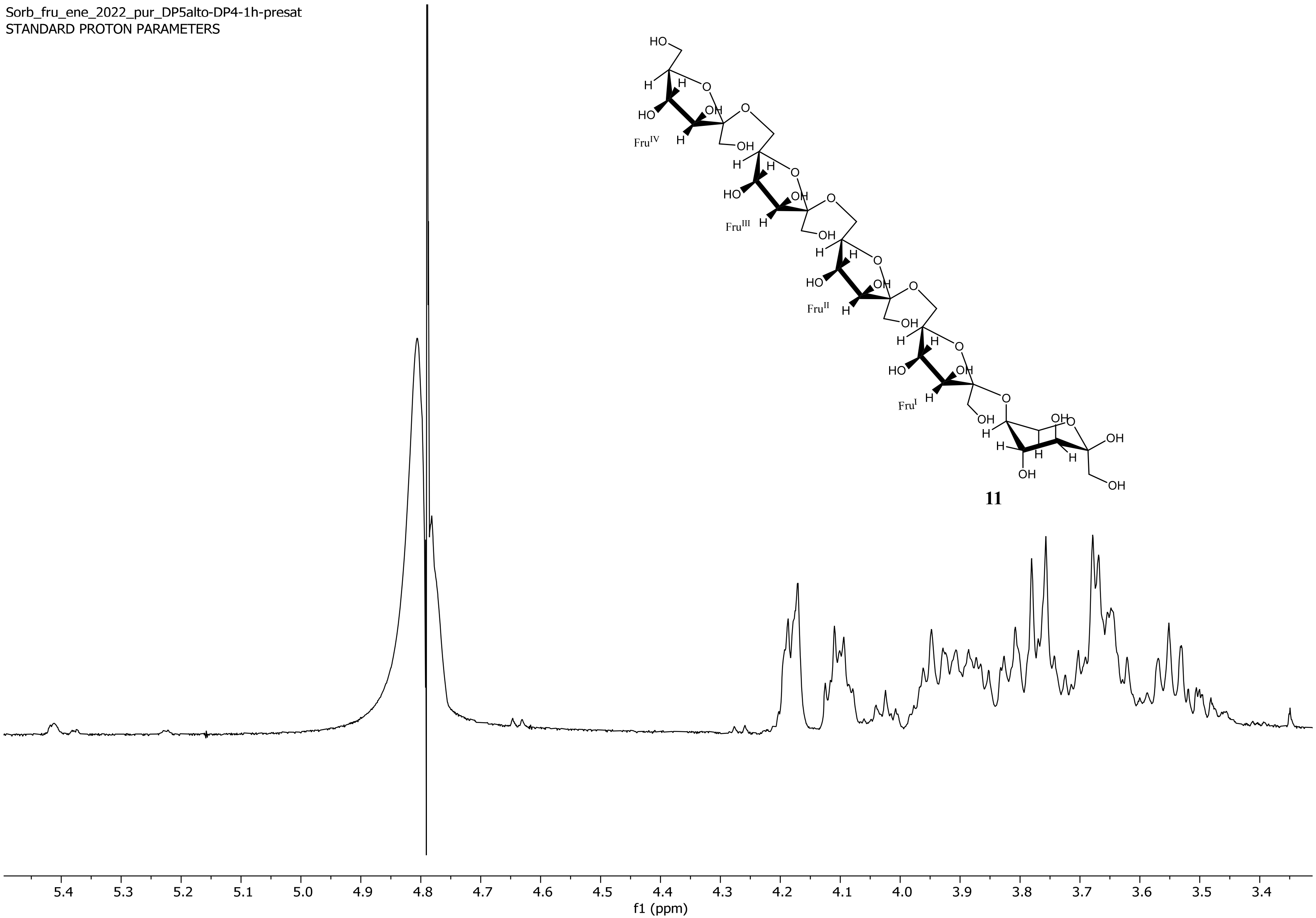

Figure S26. ^{1}H NMR (500 MHz, D_2O) for pentasaccharide **11**.

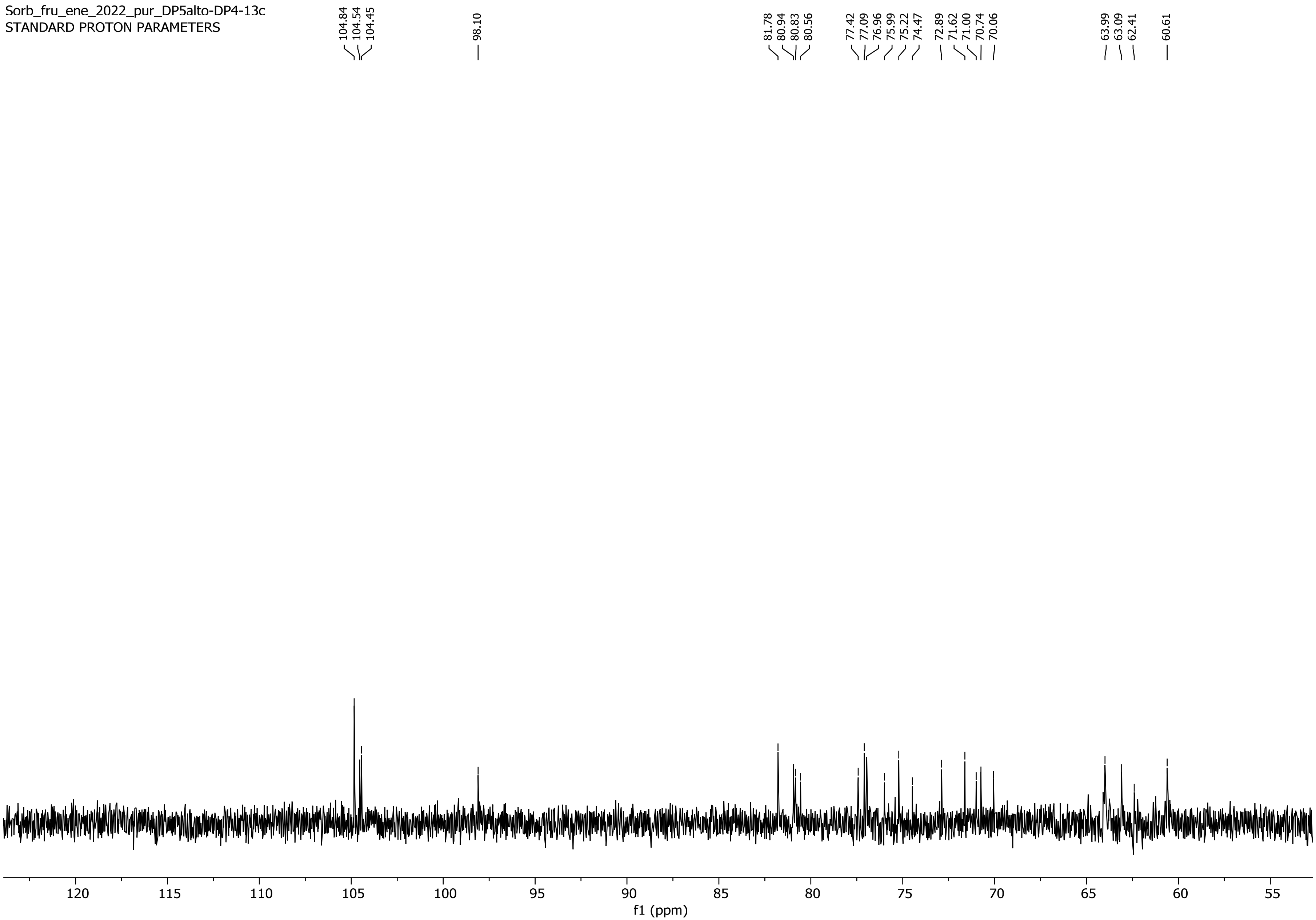

Figure S27. ^{13}C NMR (125 MHz, D_2O) for pentasaccharide **11**.

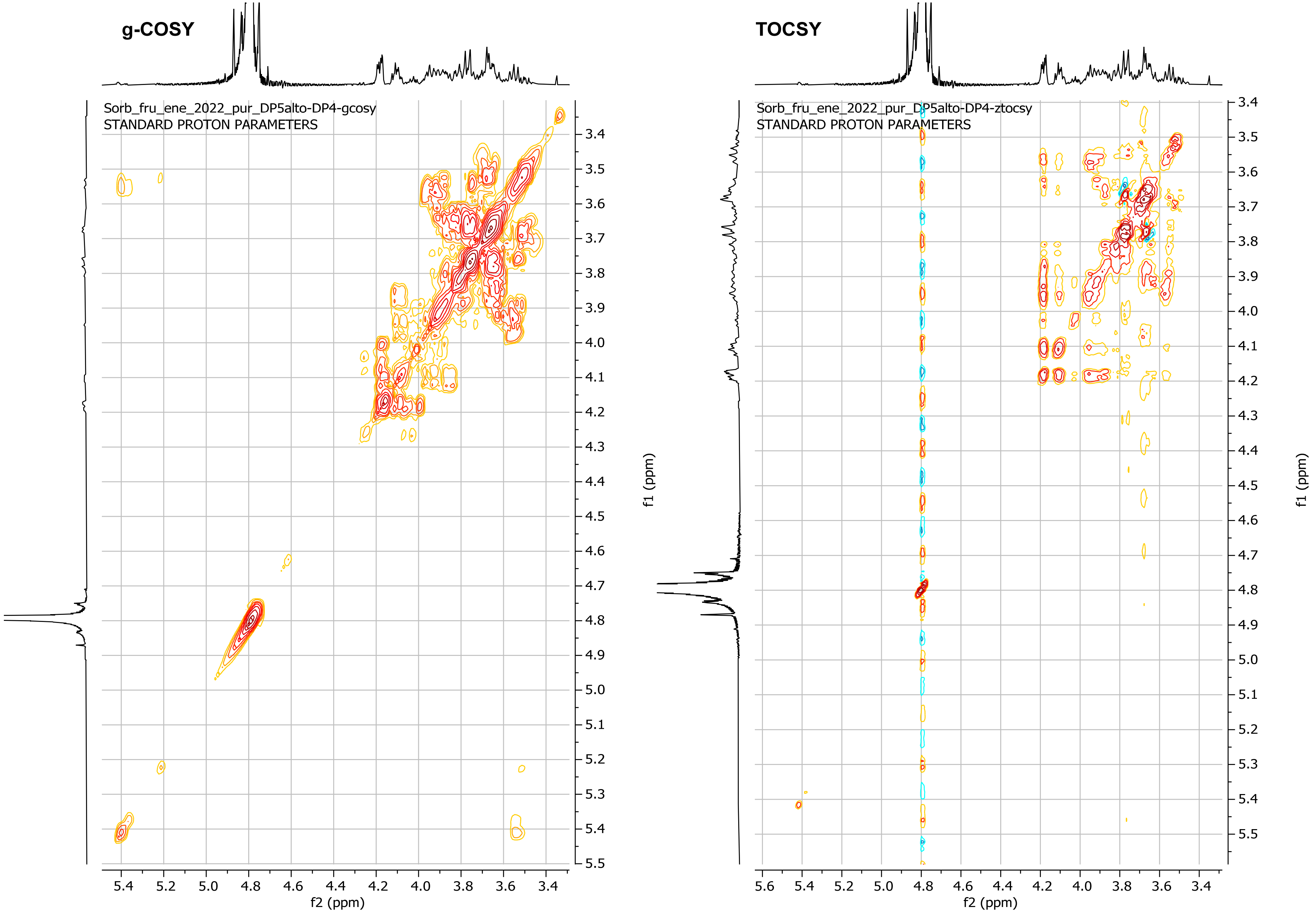

Figure S28. gCOSY and TOCSY (500 MHz, D_2O) for pentasaccharide **11**.

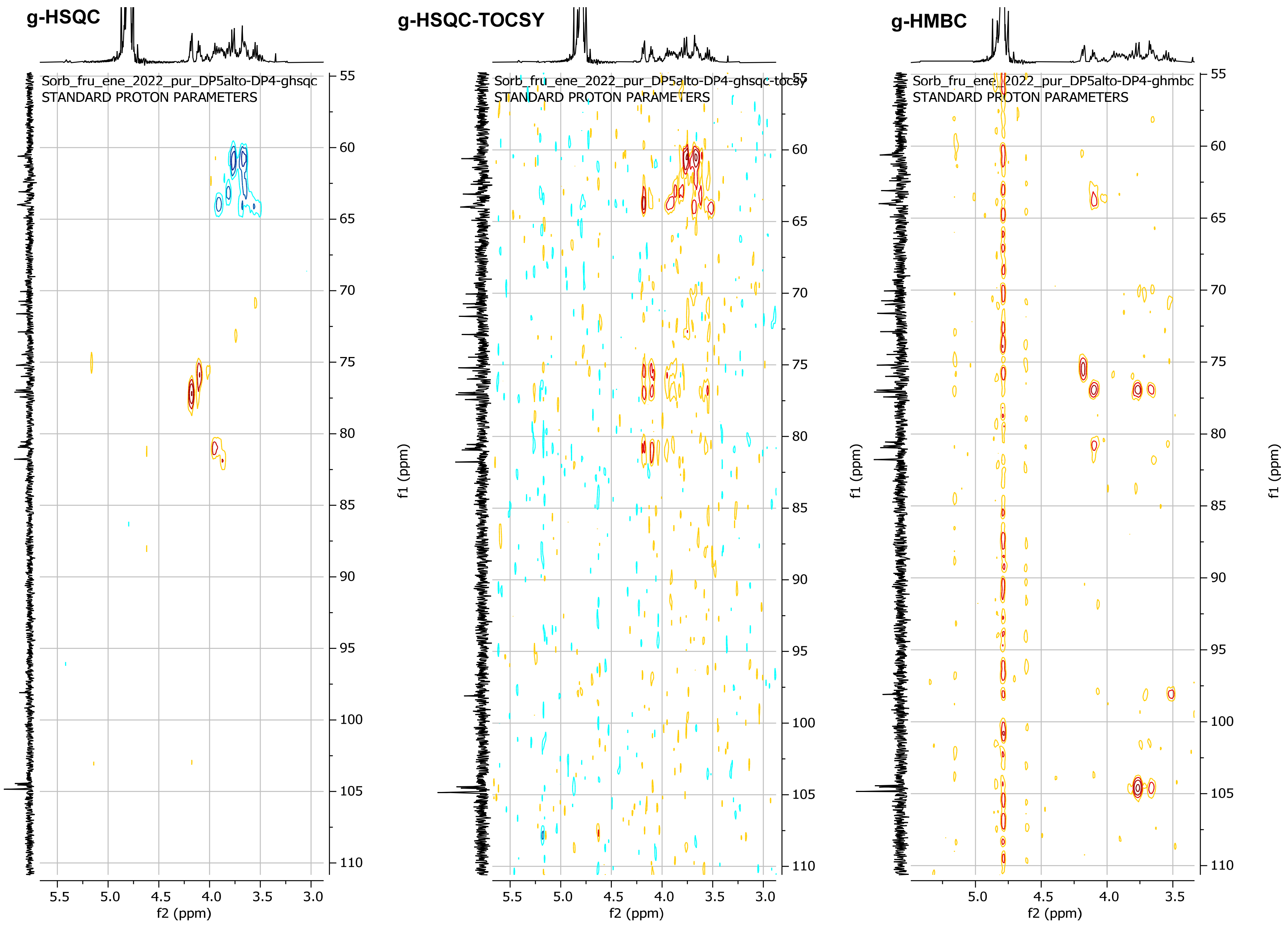

Figure S29. Multiplicity-edited gHSQC (methylene: blue cross peaks; methine: red cross peaks), gHSQC-TOCSY and gHMBC (500 MHz, D_2O) for pentasaccharide **11**.

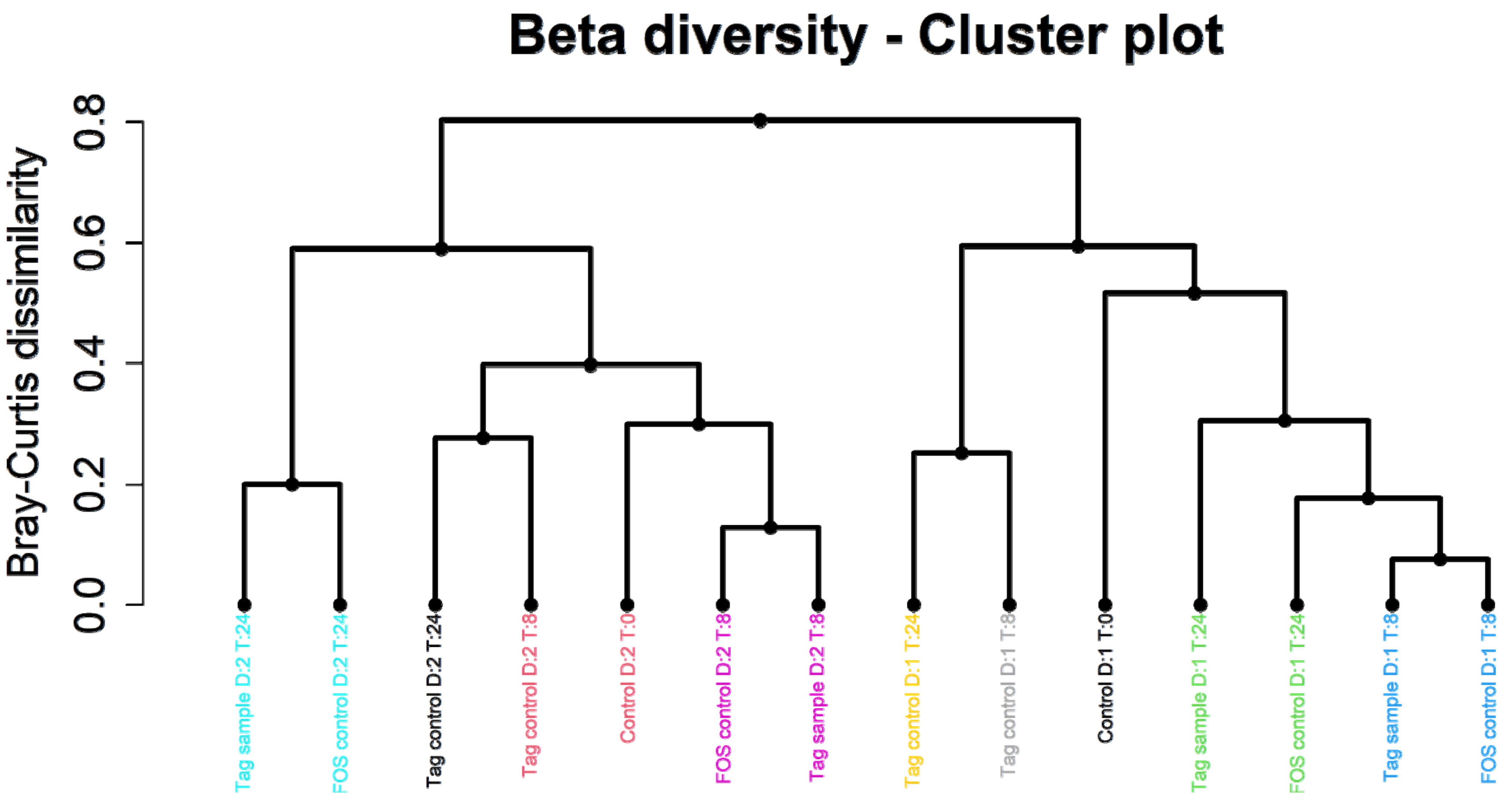

Figure S30. Beta-diversity analysis of fecal fermentations of different substrates: fructo-oligosaccharides, FOS, and unmodified tagatose controls, and modified tagatose sample. Control group corresponds to fecal pool inoculums (initial fermentation time, 0h). Bray-Curtis dissimilarity method was selected for the calculation. D: donor. T: fermentation time (h). FOS: fructo-oligosaccharides. Tag: tagatose. Tag sample: β-D-Fru-(2→1)-D-Tag.

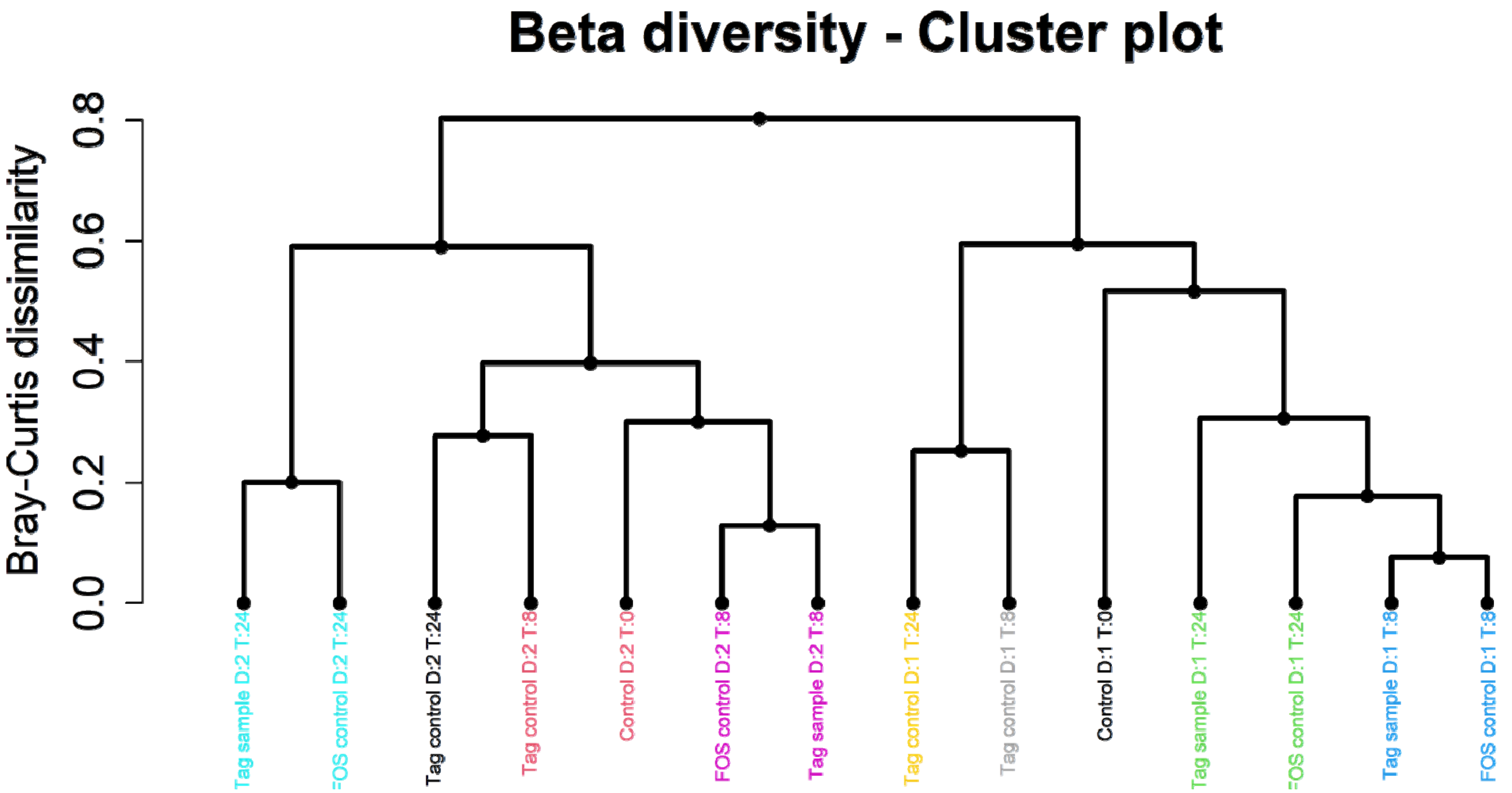

Figure S31. Cluster analysis of fecal fermentations of different substrates: fructo-oligosaccharides, FOS, and unmodified tagatose controls, and modified tagatose sample. Control group corresponds to fecal pool inoculums (initial fermentation time, 0h). Bray-Curtis dissimilarity method was selected for the calculation. D: donor. T: fermentation time (h). FOS: fructo-oligosaccharides. Tag: tagatose. Tag sample: β-D-Fru-(2→1)-D-Tag.

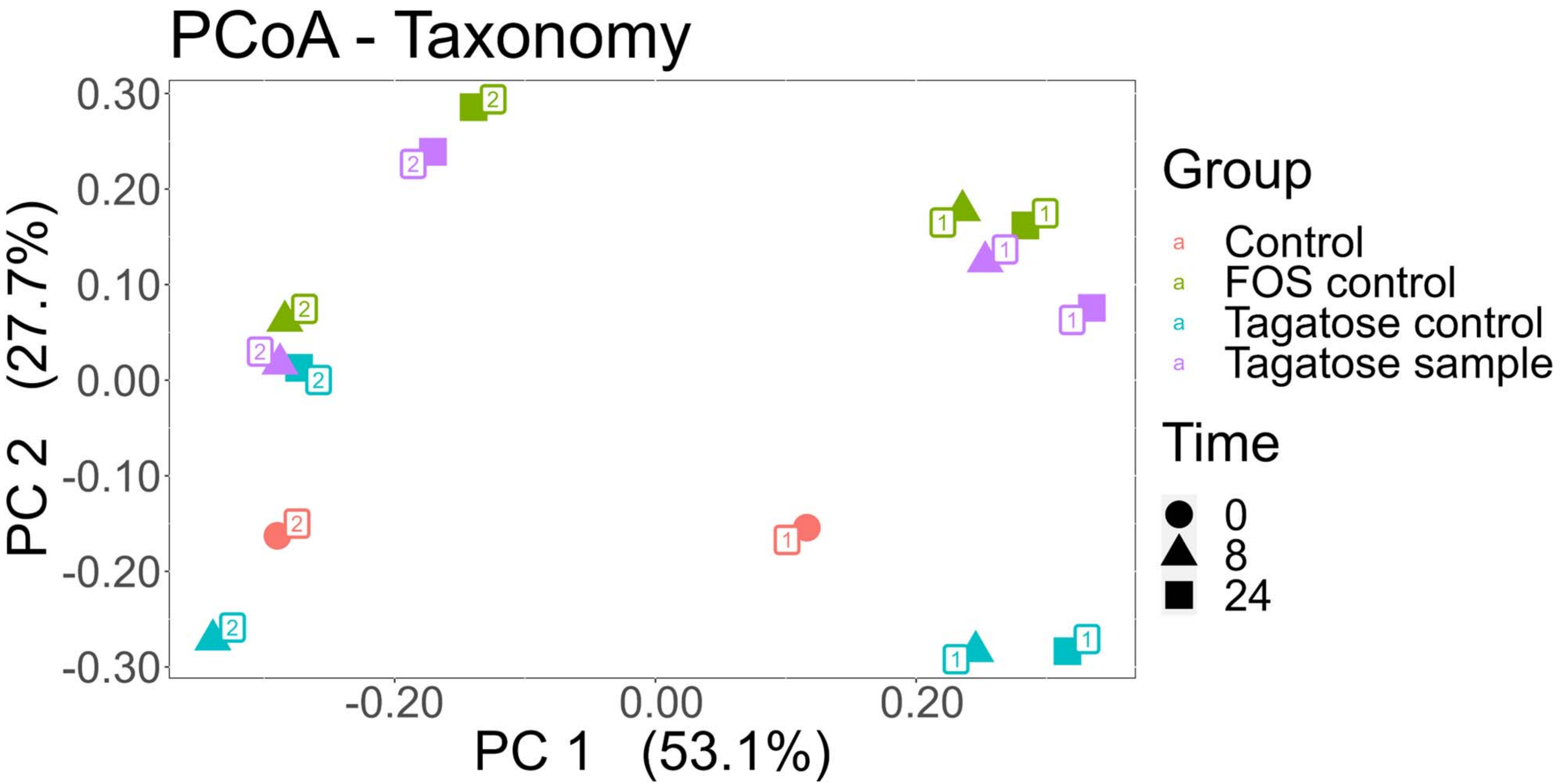

Figure S32. Principal coordinates analysis (PCoA) of fecal fermentations of different substrates: fructo-oligosaccharides, FOS, and unmodified tagatose controls, and modified tagatose sample. Control group corresponds to fecal pool inoculums (initial fermentation time, 0h). Samples corresponding to different fermentation times (0, 8, and 24h) are represented. PCoA: principal coordinate. The percentage of variance explained by each PCoA is indicated in the axis. Donor numbers corresponding to each fecal fermentation experiment are indicated in parentheses. FOS: fructo-oligosaccharides. Tag: tagatose. Tag sample: β-D-Fru-(2→1)-D-Tag.

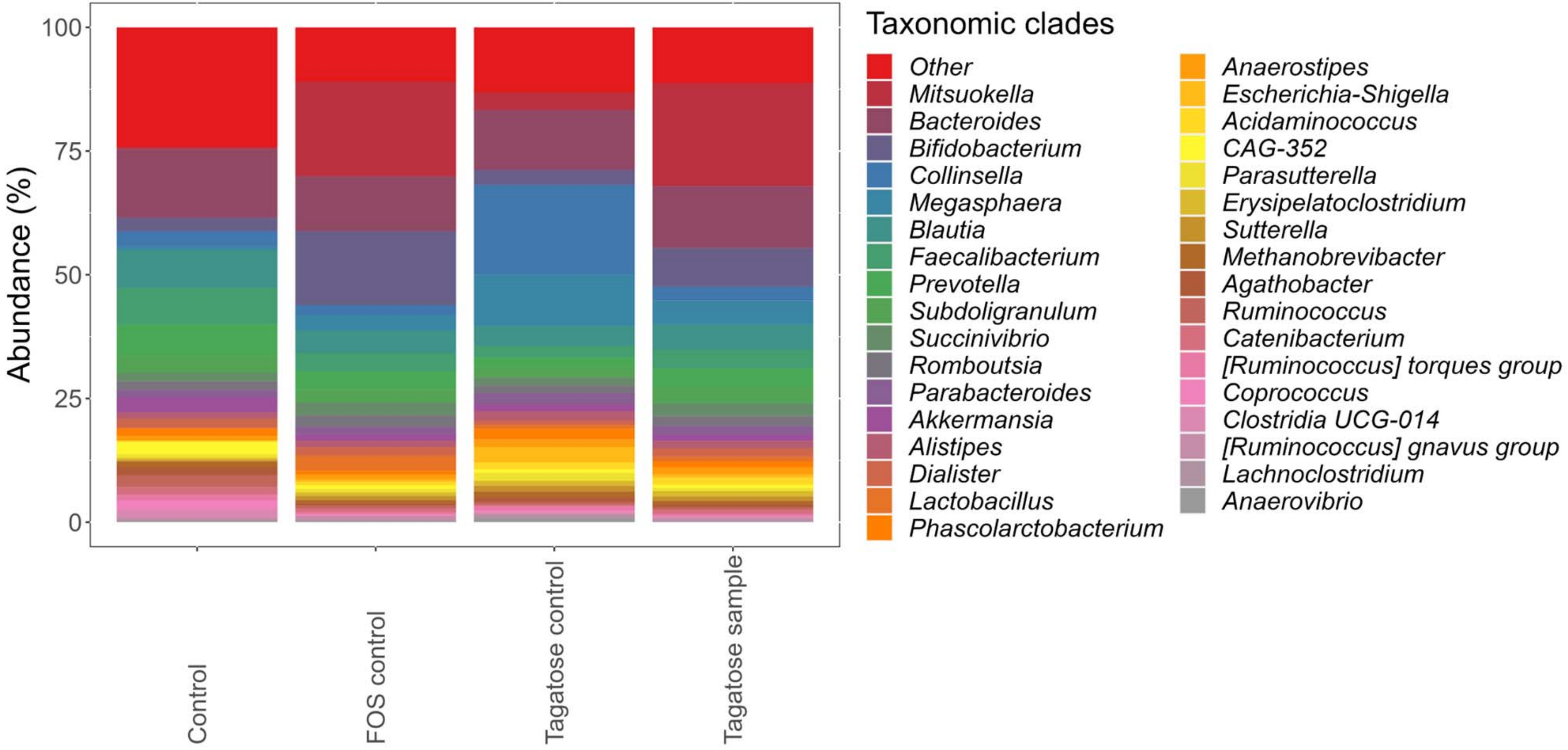

Figure S33. Most abundant genera found in fecal fermentations of different substrates: fructo-oligosaccharides, FOS, and unmodified tagatose controls, and modified tagatose sample. Control group corresponds to fecal pool inoculums (initial fermentation time, 0h). Data are expressed as abundance percentages (%). FOS: fructo-oligosaccharides. Tag: tagatose. Tag sample: β-D-Fru-(2→1)-D-Tag.

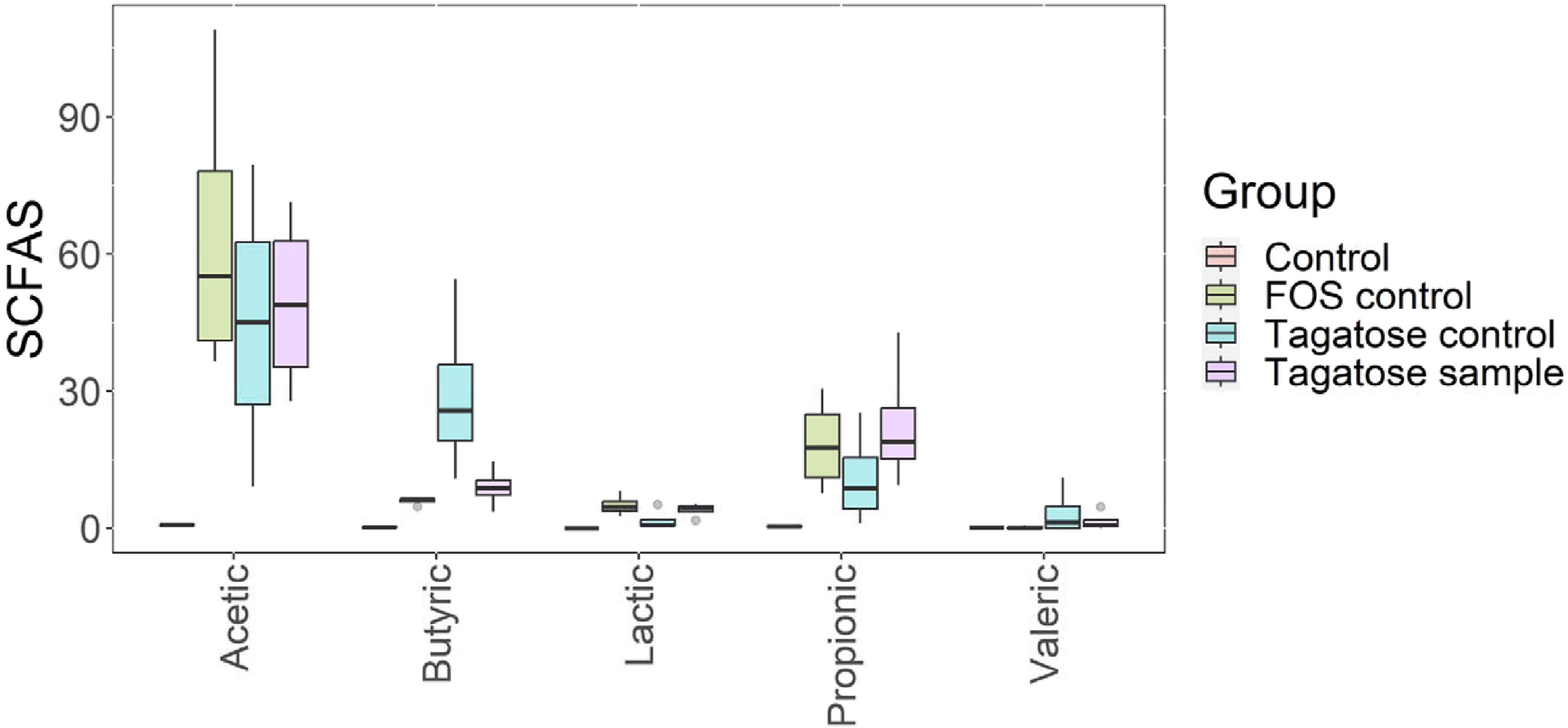

Figure S34. Short-chain fatty acids (SFCAs) concentrations (mM) determined after fecal fermentations of different substrates: fructo-oligosaccharides, FOS, and unmodified tagatose controls, and modified tagatose samples. Control group corresponds to fecal pool inoculums (initial fermentation time, 0h). SCFAs determined include acetic, butyric, formic, isovaleric, lactic, propanoic, and valeric acids. FOS: fructo-oligosaccharides. Tag: tagatose. Tag sample: β-D-Fru-(2→1)-D-Tag.
